# Lanreotide versus placebo for tumour reduction in patients with a ^68^Ga-DOTATATE PET-positive, clinically non-functioning pituitary macroadenoma (GALANT study): a randomised, multicentre, phase 3 trial with blinded outcome assessment

**DOI:** 10.1016/j.lanepe.2024.100923

**Published:** 2024-05-13

**Authors:** Tessel M. Boertien, Madeleine L. Drent, Jan Booij, Charles B.L.M. Majoie, Marcel P.M. Stokkel, Jantien Hoogmoed, Alberto M. Pereira, Nienke R. Biermasz, Suat Simsek, Ronald Groote Veldman, Annick J. Weterings, Juan M. Vink, Michael W.T. Tanck, Eric Fliers, Peter H. Bisschop

**Affiliations:** a Amsterdam UMC Location University of Amsterdam, Department of Endocrinology and Metabolism, Meibergdreef 9, Amsterdam, the Netherlands; b Amsterdam UMC Location VU University, Department of Endocrinology and Metabolism, De Boelelaan 1117, Amsterdam, the Netherlands; c Amsterdam Gastroenterology Endocrinology Metabolism, Research Programme Endocrinology, Metabolism and Nutrition, Amsterdam, the Netherlands; d Pituitary Centre Amsterdam, Amsterdam, the Netherlands; e Amsterdam UMC Location University of Amsterdam, Department of Radiology and Nuclear Medicine, Meibergdreef 9, Amsterdam, the Netherlands; f Netherlands Cancer Institute, Department of Nuclear Medicine, Amsterdam, the Netherlands; g Amsterdam UMC Location University of Amsterdam, Department of Neurosurgery, Neurosurgical Centre Amsterdam, Meibergdreef 9, Amsterdam, the Netherlands; h Leiden University Medical Centre, Division of Endocrinology, Centre for Endocrine Tumours Leiden (CETL), Leiden, the Netherlands; i Northwest Clinics, Department of Internal Medicine, Alkmaar, the Netherlands; j Medical Spectrum Twente, Department of Internal Medicine, Enschede, the Netherlands; k Amsterdam UMC Location University of Amsterdam, Department of Epidemiology and Data Science, Meibergdreef 9, Amsterdam, the Netherlands

**Keywords:** Non-functioning pituitary adenoma, Somatostatin analogue, Lanreotide, ^68^Ga-DOTATATE PET imaging, Randomised placebo-controlled trial

## Abstract

**Background:**

No established medical treatment options currently exist for patients with non-functioning pituitary macroadenoma (NFPMA). Somatostatin analogues may prevent tumour growth, but randomised controlled trials are lacking. *In vivo* somatostatin receptor assessment with ^68^Ga-DOTATATE PET could help in selecting patients for treatment. We aimed to determine the effect of the somatostatin analogue lanreotide on tumour size in patients with a ^68^Ga-DOTATATE PET-positive NFPMA.

**Methods:**

The GALANT study was an investigator-initiated, multicentre, randomised, double-blind, placebo-controlled, parallel-group, phase 3 trial with recruitment at three academic hospitals in the Netherlands. Adult patients with a suprasellar extending NFPMA, either surgery-naïve or postoperative remnant ≥10 mm, were eligible for inclusion. Important exclusion criteria were previous sellar radiotherapy and use of dopamine receptor agonists. Somatostatin receptor expression in the NFPMA was determined through ^68^Ga-DOTATATE PET/CT, co-registered with MRI. A predefined sample of 44 patients with PET-positive NFPMA were randomly assigned (1:1) to lanreotide acetate 120 mg or placebo, both administered as deep subcutaneous injections every 28 days for 72 weeks. Primary outcome was the change in cranio-caudal tumour diameter measured on pituitary MRI from baseline to end-of-treatment in the intention-to-treat population. Participants, investigators and outcome assessors were masked to treatment allocation. The trial is registered with the Netherlands Trial Registry, NL5136, and EudraCT, 2015-001234-22.

**Findings:**

Between Nov 3, 2015, and Dec 10, 2019, 49 patients were included in the study. Forty-four patients with a ^68^Ga-DOTATATE PET-positive NFPMA were randomly assigned to lanreotide (22 [50%]) or placebo (22 [50%]). Study treatment was completed in 13 (59%) lanreotide and 19 (86%) placebo participants. The mean (SD) change from baseline in cranio-caudal tumour diameter after treatment was +1·2 (2·5) mm with lanreotide and +1·3 (1·5) mm with placebo; adjusted mean difference versus placebo −0·1 mm (95% CI −1·3 to 1·2, p = 0·93). Adverse events occurred in 22 (100%, 147 events) lanreotide and 21 (95%, 94 events) placebo participants. Gastrointestinal complaints were most common, reported by 18 (82%) lanreotide and 8 (36%) placebo participants. There were no treatment-related serious adverse events.

**Interpretation:**

Compared with placebo, lanreotide treatment did not reduce tumour size or growth in patients with ^68^Ga-DOTATATE PET-positive NFPMA.

**Funding:**

Ipsen Farmaceutica BV.

**Research in context**

**Evidence before this study**

For patients with non-functioning pituitary macroadenomas (NFPMA), current treatment options apart from transsphenoidal surgery are limited. Remission rates after surgery remain modest and long-term follow-up is required due to the high propensity for tumour regrowth. Interest in medical treatment options is therefore ongoing. We systematically searched MEDLINE (PubMed), Embase, and CENTRAL for clinical studies evaluating the effect of somatostatin analogues (SSA) and dopamine receptor agonists (DA) in patients with NFPMA from inception to June 2, 2020, without language restrictions. Search terms included “non-functioning pituitary adenoma”, “non-secreting pituitary adenoma”, “silent pituitary adenoma”, “gonadotroph adenoma”, “chromophobe adenoma”, “medical treatment”, “somatostatin analogue”, “octreotide”, “lanreotide”, “pasireotide”, “dopamine agonist”, “bromocriptine”, and “cabergoline” (the complete search strategy is included in the Appendix pp 2–5). The yield of this search was low as results of well-controlled trials were lacking. A previous review of literature summarised the results of small case series and studies on SSA treatment in a total of 100 patients and on DA treatment in a total of 199 patients, showing tumour shrinkage in 12% and 28% of patients, respectively. However, interpretation of these results is severely limited by low methodological quality due to small number of patients (less than 10 in most studies), patient selection bias, and short treatment duration (average of 6–12 months). A further prospective study on the SSA octreotide with longer follow-up showed stable adenoma size in 81% of treated patients versus stability in 47% of untreated controls. A retrospective study and an open-label trial on DA treatment showed tumour shrinkage in 38% and 29% of patients, respectively. A trial registry review, repeated before submission, revealed several trials that are ongoing or have unpublished results. These include a single-arm trial with the second-generation SSA pasireotide (NCT01283542), a randomised, single-blind (outcome assessor) trial with the dopamine receptor agonist cabergoline versus observation (NCT02288962), a randomised open-label trial with pasireotide versus cabergoline (NCT01620138), and a randomised, double-blind, placebo-controlled trial with the novel chimeric dopamine-somatostatin receptor agonist TBR-760 (NCT04335357).

**Added value of this study**

The GALANT study is the first successfully completed double-blind and placebo-controlled intervention trial in patients with NFPMA. We used ^68^Ga-DOTATATE PET/CT to randomise only those patients with evidence for a somatostatin receptor-expressing NFPMA between the SSA lanreotide and placebo. There was no statistically significant difference in change in cranio-caudal tumour diameter between treatment groups. There were also no statistically significant differences in secondary outcomes, including change in tumour volume, time to tumour progression, and change in quality of life. Treatment-related adverse events were more frequent in the lanreotide group and led to study discontinuation in three participants. No treatment-related serious adverse events were reported.

**Implications of all the available evidence**

The results of the GALANT trial do not support the use of SSAs in patients with NFPMA. Results from the above-mentioned trials may provide further evidence on the topic of medical treatment for patients with NFPMA.

## Introduction

Clinically non-functioning pituitary adenomas (NFPA) are characterised by absence of clinical and biochemical signs of hormonal hypersecretion, and constitute roughly one third of all pituitary adenomas with an estimated prevalence of 25 per 100.000 people.^1^ Due to their silent nature, NFPA can go undetected for a long time and usually are macroadenomas (NFPMA), ie diameter ≥10 mm, at time of diagnosis. Presenting symptoms are related to mass effects and include pituitary insufficiency, headache, and visual disturbances.^1^ The 2022 edition of the WHO classification of endocrine and neuroendocrine tumours recommends a change in nomenclature from pituitary adenoma to pituitary neuroendocrine tumours (PitNET), but this has not yet gained widespread consensus.^2^^,^^3^

Transsphenoidal adenoma resection is the main therapeutic approach, especially in case of optic chiasm compression.^4^ However, complete resection is attained in only 30–40% of patients, and residual tumour regrowth occurs in up to 50% of patients within five years after surgery.^5^^,^^6^ Consequently, many patients require additional intervention during follow-up. Adjuvant radiotherapy can effectively control tumour (re)growth, but its use is restricted due to the high risk of hypopituitarism and rare but important complications such as optic nerve damage and secondary brain tumours.^4^^,^^7^^,^^8^

These challenges explain the ongoing interest in medical therapy as alternative treatment option for NFPMA.^7^^,^^8^ Dopamine receptor agonists have recently gained interest as possible adjuvant treatment to obtain tumour stability.^9^^,^^10^ The expression of somatostatin receptors (SSTR) in the majority of NFPA provides a further potential target for receptor-mediated therapy with somatostatin analogues (SSA).^11^, ^12^, ^13^, ^14^, ^15^, ^16^ SSTR subtypes found most commonly in NFPA samples are SSTR2, SSTR3, and SSTR5, all three of which are implicated in antiproliferative effects.^11^, ^12^, ^13^^,^^15^^,^^17^ *In vitro* studies have demonstrated inhibition of cell viability and proliferation in response to octreotide and lanreotide, two first-generation SSAs with highest affinity for SSTR2.^18^^,^^19^ Octreotide treatment may indeed prevent tumour progression in selected patients with NFPMA, but results have been inconclusive due to methodological limitations.^11^^,^^20^

SSAs could thus play a role in the management of NFPMA, but outcomes of well-designed placebo-controlled studies are lacking. A promising approach may be *in vivo* SSTR imaging to select patients potentially responsive to treatment.^11^^,^^16^ Previously, ^111^In-DTPA-octreotide planar scintigraphy or single photon emission computed tomography (SPECT) has been used to assess SSTR expression.^11^^,^^14^ However, interpretation of pituitary adenoma uptake using these techniques is limited by low spatial resolution.^21^ The introduction of the positron emission tomography (PET) radiotracer ^68^Ga-DOTATATE (Gallium-68-labeled dodecanetetraacetic acid-tyrosine-3-octreotate) has enabled high-resolution PET/CT imaging with additional superb SSTR2 affinity.^22^ The aim of this study was to determine the effect of the long-acting SSA lanreotide on tumour size in patients with a ^68^Ga-DOTATATE PET-positive NFPMA.

## Methods

### Study design and participants

The GALANT trial was an investigator-initiated, multicentre, randomised, double-blind (participants, investigators and outcome assessors), placebo-controlled, parallel-group, phase 3 trial. Recruitment took place at three tertiary, academic hospitals in the Netherlands: Amsterdam University Medical Centre (Amsterdam UMC) locations AMC and VUmc, and Leiden University Medical Centre. Treating endocrinologists at academic and non-academic hospitals throughout the Netherlands could refer eligible patients for inclusion at one of these participating centres. The Netherlands Cancer Institute in Amsterdam was a fourth participating centre to facilitate ^68^Ga-DOTATATE PET/CT scans after trial initiation, with no role in patient recruitment.

Adult patients (≥18 years) diagnosed with an NFPMA, either surgery-naïve or postoperative remnant, with current suprasellar extension and largest diameter ≥10 mm, were eligible for inclusion. Eligible patients were approached by their treating endocrinologist and, if permission was granted, informed in full by a trial staff member (TMB). NFPMA diagnosis was based on evidence for a pituitary macroadenoma on dedicated pituitary magnetic resonance imaging (MRI) with absence of clinical and biochemical signs of hormonal hypersecretion. As upward growth towards the optic nerves and chiasm presents the most important indication for treatment in NFPMA, extension above the sellar diaphragm was considered a relevant additional inclusion criterion. In case of current optic nerve or chiasm compression, visual field defects or other visual disturbances had to be ruled out before inclusion. Other important exclusion criteria were previous or planned radiotherapy of the pituitary region, any previous use of SSAs, or use of dopamine receptor agonists in the past six months, as these treatments could influence the primary outcome. Premenopausal female patients were excluded if they were pregnant, lactating, or not using adequate methods of contraception. Further exclusion criteria were hypersensitivity to somatostatin or similar peptides, known diagnosis of symptomatic cholelithiasis or an obstructive neuroendocrine gut tumour, any contraindication to undergo MRI with a gadolinium-based contrast agent, or inability to provide informed consent.

All participants provided written informed consent. The study protocol and subsequent amendments were approved centrally by the ethics committee of Amsterdam UMC location AMC (METC 2015_103) and the Central Committee on Research Involving Human Subjects (NL52821.018.15), and locally by the boards of directors of the participating centres (see Appendix p 5 for an overview of substantial protocol amendments). The study was conducted in accordance with the International Conference on Harmonisation Good Clinical Practice guidelines and the principles of the Declaration of Helsinki. Data were collected through Castor Electronic Data Capture (Castor EDC, Amsterdam, The Netherlands; www.castoredc.com). The study was monitored by an independent monitor of the Clinical Research Unit of the Amsterdam UMC location AMC. The trial was registered before start of recruitment with the Netherlands Trial Registry (NL5136) and with EudraCT (2015-001234-22). The study protocol has been published previously.^23^

### Randomisation and masking

Forty-four participants with a ^68^Ga-DOTATATE PET-positive NFPMA were randomly assigned to lanreotide 120 mg (lanreotide group) or placebo (placebo group) injections. Randomisation was performed centrally by the Trial Pharmacy of the Amsterdam UMC location AMC, using a computer-generated list through Sealed Envelope Ltd. with an allocation ratio of 1:1 and block size of four. The randomisation list was stored in a secure trial file at the Pharmacy and was disclosed after database lock via a written request and confirmation by the principal investigator. Participants, investigators, and outcome assessors thus remained blinded throughout the trial. Each participant was assigned a randomisation code used to order study medication at the Trial Pharmacy. As the prefilled lanreotide syringes differed in appearance from the placebo, injections were administered by trained, independent nurses who were unmasked to treatment allocation, a method also used in the CLARINET trial.^24^ To maintain blinding during transport, prepared study medication was placed in an opaque bag within a sealed cardboard box. Furthermore, injections were administered out of view of the participant in the superior, external quadrant of the buttock. Participants had no earlier experience with lanreotide treatment to have expectations regarding the administration.

### Procedures

At the screening visit, participants underwent ^68^Ga-DOTATATE PET/CT of the head. This was performed at the Netherlands Cancer Institute or at the Amsterdam UMC location AMC. Imaging technique and analysis are described in more detail in previous publications regarding this study.^23^^,^^25^ In short, after coregistration of PET/CT and pituitary MR images using Hybrid Viewer (Hermes Medical Solutions, version 2·8·2 and above; Stockholm, Sweden), a circular region of interest was manually drawn within the NFPMA to determine the mean standard uptake value (SUV_mean_). Positive ^68^Ga-DOTATATE uptake was defined as an NFPMA SUV_mean_ of >2. In the absence of literature on ^68^Ga-DOTATATE uptake in pituitary adenomas, this value was chosen to reflect an uptake level at least similar to that of the normal pituitary as reported in a ^68^Ga-DOTATATE biodistribution study.^26^ For characterisation purposes, maximum standard uptake value (SUV_max_) in the NFPMA was assessed as well. Image analysis was done by the same trial staff member for all participants (TMB, under supervision of JB). Only patients with a PET-positive NFPMA were randomised for treatment.

Participants in the lanreotide group were treated with the extended-release formulation of lanreotide acetate 120 mg (Somatuline AutoSolution, Ipsen Farmaceutica BV; known outside the Netherlands as Somatuline Autogel), without dose titration. Placebo treatment consisted of 0·4 mL saline 0·9%. Both were administered every 28 days as a deep subcutaneous injection. Administration was performed either at the endocrine unit of the Amsterdam UMC location AMC, or at home via a homecare service (Eurocept Homecare). All administrations were recorded in a blinded manner by the independent nurses. Total treatment consisted of 18 injections, from week 0 to week 68.

Study visits after randomisation took place at the centre of inclusion and were planned at baseline, week 24, week 48, and week 72 (ie, before start of treatment, and four weeks after the 6th, 12th, and 18th injection, respectively). All visits included a fasting blood sample, measurement of weight, pulse rate and blood pressure, quality of life questionnaire, and a semi-structured interview focused on adverse events and changes in medication use. The laboratory assessments were part of standard care and included an endocrine evaluation, fasting glucose, HbA_1c_, kidney function, liver enzymes, and bilirubin. The 36-item Short Form Health Survey (SF-36) was used to assess quality of life.^27^ The SF-36 consists of 36 items that generate eight component scores: physical functioning, role limitations due to physical health problems, bodily pain, general health perceptions, vitality, social functioning, role limitations due to emotional problems, and general mental health. Scores are converted to a 0–100 scale, with higher scores indicating better quality of life for that component. Pituitary MRI was performed at baseline, week 24 and week 72. As regular pituitary MRIs were part of standard care, study participation was timed to have visits coincide with regular MRI planning where possible. If the time between the most recent MRI and planned start of study treatment exceeded three months, efforts were made to repeat the MRI for baseline measurement. MRI scans were performed on a 1·5 or 3 T scanner, following a pituitary-specific protocol with T1-weighted coronal and sagittal acquisitions (slice thickness 3 mm) before and after gadolinium administration, a T2-weighted coronal sequence, and preferably included a gadolinium-enhanced volumetric 3DT1-weighted sequence (slice thickness 1 mm).

Standard patient care was continued throughout the study. Additional pituitary MRI and visual field examination were planned if visual disturbances occurred. If (surgical) intervention was deemed necessary due to tumour progression, study treatment was discontinued. Participants could also be withdrawn on their own request or based on the investigator's or treating specialist's judgement (eg, following an adverse event that could jeopardise the participant's safety). In case of treatment discontinuation, a premature end-of-study visit was planned in the month after the last received injection to obtain outcome data, unless assessments were performed within the previous four weeks.

Some flexibility in the schedule was permitted to allow for convenient (regular care) planning and holidays, aiming for a maximum of seven days before or after an originally planned injection or visit. When restrictions following the COVID-19 pandemic intensified, injections were administered at home for all participants for the remainder of the study and onsite visits were limited or postponed, if necessary. Protocol deviations are described in the Appendix p 6.

### Outcomes

Primary outcome was the change in cranio-caudal tumour diameter from baseline to week-72 or treatment discontinuation. Secondary outcomes were change in tumour volume, time to tumour progression, and change in quality of life based on the SF-36 component scores.

Cranio-caudal diameter and tumour volume were centrally assessed on pituitary MRIs by two independent assessors who were additionally blinded to scan chronology (AJW and JMV). Measurements were performed using ITK-SNAP (version 3·8·0; Philadelphia, PA, USA; www.itksnap.org).^28^ Cranio-caudal diameter was defined as the maximum height of the tumour in between the left and right cavernous internal carotid arteries, measured in the sagittal plane on the gadolinium-enhanced T1-weighted sequence. Tumour volume was measured on the gadolinium-enhanced 3DT1-weighted sequence using a region-based, semi-automatic segmentation tool within ITK-SNAP, with post-segmentation manual multiplane slice-by slice adjustment. If a volumetric sequence was missing for one of the scans, all volume measurements for that participant were performed on the regular gadolinium-enhanced T1-weighted images via multiplane slice-by-slice manual tumour segmentation. For both cranio-caudal diameter and tumour volume, measurements of both assessors were averaged for analysis. In case of a between-reader difference of ≥10%, and/or ≥2 mm for cranio-caudal diameter, both assessors performed a second review. In case of persistent discrepancies, a third reader would adjudicate. Clinically significant change in tumour size was defined as a decrease or increase of ≥2 mm in cranio-caudal diameter or ≥20% in tumour volume.^29^^,^^30^ Time to progression was defined as the interval between start of study treatment and the first subsequent MRI scan showing a clinically significant increase in tumour volume.

Harms were assessed on the basis of the number, type and severity of adverse events (AEs), coded using the Medical Dictionary for Regulatory Activities (version 24·0). AEs were assessed systematically at each study visit (see Procedures) and non-systematically through self-reporting at any time during study participation. Any undesirable event, finding, or change from baseline (eg, worsening of known dyspepsia) occurring between enrolment and up to 30 days after treatment completion or discontinuation was considered an AE. Recurrent events in the same participant were counted as separate AEs. Prespecified AEs of special interest included injection site reactions, gastrointestinal disorders, cholelithiasis, and hyperglycaemia or hypoglycaemia.

### Statistical analysis

The trial was powered on a between-group difference of ≥2 mm in primary outcome (ie, change from baseline in cranio-caudal tumour diameter). This value was chosen based on its clinical relevance in preventing complications related to compression of the optic nerves or chiasm following upwards tumour growth.^4^^,^^7^ Furthermore, it reflects a reliable detection limit on consecutive pituitary MRIs.^29^ To detect a 2 mm target difference with an estimated standard deviation (SD) of 1·9 mm (following MRI resolution limitations and the use of 3 mm slice thickness sequences with 1 mm in-plane resolution), yielding a standardised effect size of 1·1, we needed to randomise 16 patients with a ^68^Ga-DOTATATE PET-positive NFPMA per group based on a two-sided independent t-test with 80% power and 5% type I error risk. During the study, the sample size was amended to 22 patients per group following an observed overall dropout rate of 25%, in order to maintain enough power. We did not use ANCOVA for the power calculation, as an inaccurate estimate of rho (ie, correlation between covariate and dependent variable) would have resulted in an underpowered study due to underestimation of the required sample size.^31^

Statistical analyses were prespecified in the study protocol^23^ and statistical analysis plan, or reported as ‘post-hoc’. Data of continuous variables were summarised as either mean (SD) or median (IQR), according to distribution. Data of categorical variables were presented as incidence rates (number and percentage). Baseline characteristics and ^68^Ga-DOTATATE PET/CT results of all included patients were reported. Given the small number of PET-negative NFPMA, no statistical tests were performed on differences between patients with positive and negative uptake. The intention-to-treat population was the basis for primary analyses and included all randomised participants who received at least one study injection. The safety population for the assessment of harms comprised the same set of participants.

For the main analyses of the primary and secondary outcomes, all data up to treatment discontinuation was included (ie, ‘while-on-treatment’ strategy^32^). The primary outcome change in cranio-caudal diameter and secondary outcome change in tumour volume were analysed using an analysis of covariance (ANCOVA) model to control for any chance imbalance in baseline size between the groups, with end-of-treatment measurement as dependent variable, baseline measurement as continuous covariate, and treatment as categorical covariate.^33^

Additional analyses explored the impact of incomplete week-72 data due to treatment discontinuation (further details available in the Appendix pp 8–10). A per-protocol analysis using the same ANCOVA model assessed potential treatment efficacy, including only those participants who completed study treatment and underwent week-72 MRI (deviations in visit time windows were allowed). A supplementary efficacy analysis applied univariate multiple imputation to impute missing week-72 MRI data, assuming missingness at random (MAR). Data were imputed separately in each treatment group using a regression model, and 27 imputed datasets were generated (corresponding to 27% of missing week-72 data). Each dataset was assessed with the earlier specified ANCOVA model and results were pooled using Rubin's rules. A sensitivity analysis was performed for the primary outcome via a pattern-mixture model to explore departures from the MAR assumption and address potential bias due to differential dropout between treatment groups. Herein, the imputed MAR data was shifted by a range of offsets to assess alternative post-dropout scenarios of tumour behaviour. Results under MAR were considered robust if an observed treatment effect was qualitatively maintained for a range of plausible offsets.

In addition to the multiple imputation approach, efficacy for the primary outcome was assessed via a linear mixed effects model to account for repeated MRI measurements obtained at varying time-points. Post-baseline cranio-caudal diameter was modelled with treatment group, measurement time (as the number of injections after which the measurement was obtained), group-by-time interaction, baseline cranio-caudal diameter, and baseline diameter-by-time interaction as fixed effects, a by-subject random intercept, and a first-order autoregressive residual autocorrelation structure (Appendix pp 9–10). Treatment effect was estimated as the contrast between adjusted group means at the final measurement time (corresponding to week-72).

Between-group difference in secondary outcome time to tumour progression (based on significant increase in tumour volume) was analysed using the stratified log-rank test, with stratification for presence or absence of documented tumour growth at baseline (defined as growth in any direction on pituitary MRIs performed up to three years before inclusion). The hazard ratio and confidence interval were derived from a Cox proportional-hazards model with terms for treatment group and tumour growth at baseline. As a post-hoc analysis, time to progression based on significant increase in either tumour volume or cranio-caudal diameter was performed using the same method. For secondary outcome change in quality of life, baseline and end-of-treatment SF-36 component scores were presented per group as spider charts, together with age-adjusted Dutch population reference values.^27^ The mean imputation method was used to replace missing component score values up to treatment discontinuation. Change from baseline in component scores was compared between groups using ANCOVA with end-of-treatment score as dependent variable, baseline score as continuous covariate, and treatment as categorical covariate. Regarding harms, the total number of AEs and the proportion of participants experiencing a specific event were summarised. Following the CONSORT Harms 2022 statement, between-group absolute risk difference estimates with Wilson 95% confidence intervals were provided (post-hoc analysis). Due to events being rare or null in one or both treatment arms, relative risk estimates were not calculated.^34^

There were no interim or subgroup analyses. Data were analysed with SPSS (version 28), and the linear mixed model was run in R (version 4·3·0). The statistical significance level for analyses was set at p = 0·05 (two-sided). There was no need for multiplicity adjustments.

### Role of the funding source

The study was investigator-initiated with the Amsterdam UMC location AMC as sponsor. Funding was provided by Ipsen Farmaceutica BV. Ipsen had no role in final study design, data collection, analysis and interpretation, manuscript preparation, or the decision to submit for publication.

## Results

**Fig. 1:**
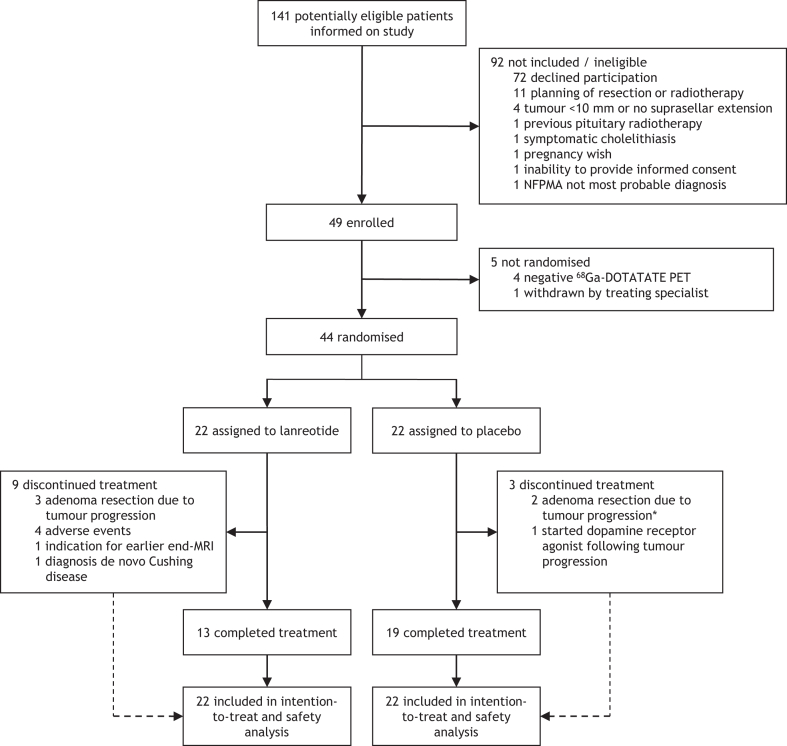
Trial profile. For all participants who discontinued treatment, outcomes were assessed at time of treatment discontinuation. NFPMA, non-functioning pituitary macroadenoma. PET, positron emission tomography. ∗In one of these participants the pathology results revealed a pituicytoma.

Between Nov 3, 2015, and Dec 10, 2019, 141 potentially eligible patients were informed on the study, of whom 49 were included (Fig. 1). Positive ^68^Ga-DOTATATE uptake within the NFPMA was found in 45 (92%) participants, with SUV_mean_ ranging from 2·1 to 14·4 (Appendix p 12). Forty-four participants with a PET-positive NFPMA were randomly assigned to the lanreotide group (22 [50%]) or placebo group (22 [50%]). One patient was withdrawn before randomisation by his treating endocrinologist following tumour progression on a repeated MRI. All randomised participants received at least one study injection and were included in the intention-to-treat and safety population. Treatment was completed in 13 (59%) participants in the lanreotide group and in 19 (86%) participants in the placebo group (per-protocol population). Treatment discontinuation due to tumour progression occurred in three lanreotide and three placebo participants. Four participants in the lanreotide group discontinued treatment due to AEs and two due to other reasons (Fig. 1). Detailed reasons for and timing of treatment discontinuation are summarised in the Appendix (p 7). In two patients the diagnosis of NFPMA was revoked at study discontinuation: one participant in the lanreotide group was diagnosed with Cushing disease after previous resection of a silent corticotroph adenoma. The other patient, in the placebo group, underwent resection following tumour progression on week-24 MRI with pathology results revealing a rare pituicytoma.

Baseline characteristics of the intention-to-treat population are reported (Table 1). Post-baseline MRI outcome data were available for all participants. Pituitary MRIs showed no signs of newly formed cysts or apoplexy during treatment. For 32/44 (73%) participants, all scans included a 3DT1-weighted volumetric sequence. Out of 124 MRI scans acquired in the 44 participants, second reviews were necessary for six cranio-caudal and 27 volume measurements. There were no persisting between-reader differences requiring additional review.

**Table 1:** Baseline characteristics of the intention-to-treat population.

	Lanreotide (n = 22)	Placebo (n = 22)
Age, years	58·8 (8·2)	63·5 (8·5)
Female sex	10 (46%)	6 (27%)
History of diabetes mellitus	3 (14%)	2 (9%)
Any pituitary hormone deficiency	15 (68%)	11 (50%)
**NFPMA characteristics**		
Time since diagnosis, months^a^	56·5 (7·5–101·4)	28·6 (8·9–84·4)
Previous resection	15 (68%)	9 (41%)
Re-resections	1/15	3/9
**Baseline NFPMA size**		
Cranio-caudal diameter, mm	16·2 (13·4–20·6)	16·3 (14·8–19·3)
Tumour volume, mm^3^	2785 (1868–4067)	2722 (1937–3967)
Documented tumour growth in last 3 years^b^	8 (36%)	6 (27%)
^**68**^**Ga-DOTATATE PET results**		
NFPMA SUV_mean_	6·1 (3·2–8·1)	5·0 (2·7–6·7)
NFPMA SUV_max_	7·9 (5·0–11·0)	6·4 (3·5–9·1)
**Centre of inclusion**		
Amsterdam UMC location AMC	15 (68%)	16 (73%)
Amsterdam UMC location VUmc	1 (5%)	3 (14%)
Leiden University Medical Centre (LUMC)	6 (27%)	3 (14%)

Data are mean (SD), median (IQR), n (%), or n/N. NFPMA = non-functioning pituitary macroadenoma. PET = positron emission tomography. SUV = standard uptake value.

a Calculated as time between first imaging with evidence for an NFPMA and the first study injection.†Visual field defects were excluded prior to enrolment.

b Tumour growth in any direction based on available MRI reports up to three years previous to and including study baseline MRI; in case of previous tumour resection within these three years, only MRIs since resection were evaluated for tumour growth.

On average, NFPMA size had increased after study treatment in both groups (Table 2). There was no statistically or clinically significant difference between treatment groups in the change in cranio-caudal tumour diameter from baseline to end-of-treatment (adjusted mean difference versus placebo −0·1 mm [95% CI −1·3 to 1·2]; p = 0·93). Change in tumour volume did not differ significantly either (p = 0·94). The additional efficacy analyses and sensitivity analyses for missing data supported the main results (Appendix p 13).

**Table 2:** Primary and secondary tumour size outcomes in the intention-to-treat population.

	Lanreotide (n = 22)	Placebo (n = 22)	Adjusted mean difference in change versus placebo (95% CI)^a^	p value
**Primary outcome**				
End cranio-caudal diameter, mm	17·3 (12·7–22·6)	17·5 (15·7–20·9)	–	–
Change in cranio-caudal diameter, mm	1·2 (2·5)	1·3 (1·5)	−0·1 (−1·3 to 1·2)	0·93
**Secondary outcome**				
End tumour volume, mm^3^	3484 (1844–4496)	3018 (2434–4277)	–	–
Change in tumour volume, mm^3^	424 (61–811)	181 (19–738)	19 (−422 to 486)^b^	0·94
Tumour volume percentage change, %	17·2 (1·5–29·7)	7·8 (0·7–16·2)	–	–

Data are mean (SD), median (IQR), or mean difference (95% confidence interval). The main analysis included all data up to treatment discontinuation.

a Adjusted for baseline tumour size using ANCOVA.

b Tumour volume values were natural log-transformed before analysis due to non-normal distribution with moderate positive skewness, the back-transformed estimated mean difference and 95% CI are reported.

**Fig. 2:**
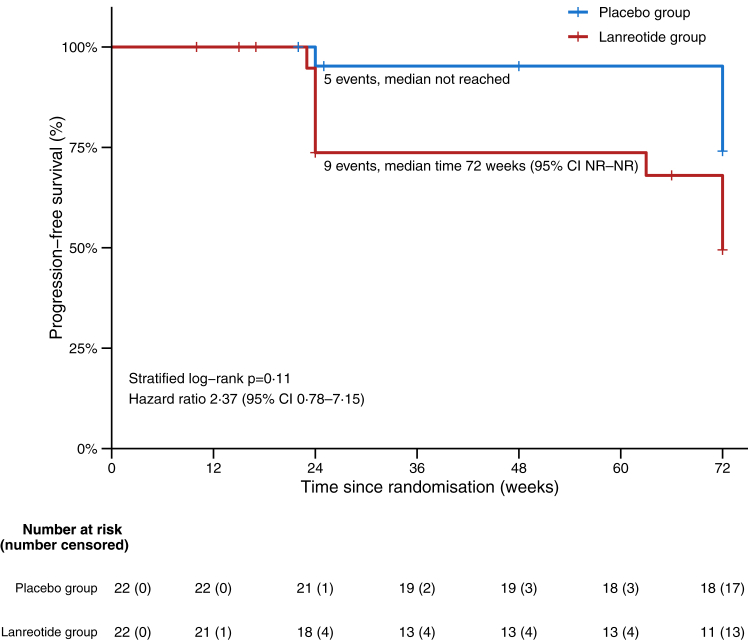
Kaplan–Meier estimates of progression-free survival for the secondary outcome time to tumour progression based on significant increase in tumour volume (ie, ≥20%). Tick marks indicate censored data. Outcome data at time of censoring was available for all participants. Time to progression was compared between groups using the stratified log-rank test, with stratification for presence or absence of documented tumour growth at baseline. The hazard ratio was derived from a Cox proportional-hazards model with terms for study treatment and tumour growth at baseline; there was no statistically significant interaction between these terms. NR, not reached.

Clinically significant decrease in either cranio-caudal diameter or tumour volume occurred in three (14%) participants in the lanreotide group and in none in the placebo group, while significant increase was seen in eleven (50%) lanreotide- and eight (36%) placebo-treated participants (Appendix p 17). Time to tumour progression, based on significant volume increase, did not differ between groups (stratified log-rank test p = 0·11; hazard ratio for progression with lanreotide versus placebo, 2·37 [95% CI 0·78–7·15]) (Fig. 2). Results were similar when tumour progression was based on significant increase in either tumour volume or cranio-caudal diameter (post-hoc analysis; Appendix p 18).

On average, quality of life component scores decreased slightly in both groups during the course of the study, without statistically significant between-group differences in change from baseline (Appendix p 14 and p 19).

A total of 241 AEs were reported during the study: 147 in 22 (100%) participants in the lanreotide group and 94 in 21 (95%) participants in the placebo group (Table 3). An overview of all AEs recorded during the study is provided in the Appendix (pp 15–16). Of all AEs, 91 were deemed possibly or probably treatment-related AEs while the study was blinded: 76/147 (52%) in 22 (100%) lanreotide participants and 15/94 (16%) in 10 (45%) placebo participants. The most common AEs were gastrointestinal disorders, occurring more frequently in the lanreotide group than in the placebo group (18 versus 8 participants). The majority of these symptoms were mild and transient. However, three participants in the lanreotide group discontinued treatment due to gastrointestinal intolerance. An additional lanreotide participant withdrew because of complaints of light-headedness and parosmia, which were deemed not treatment-related. Regarding other prespecified and systematically assessed AEs, a higher proportion of participants in the lanreotide group experienced impaired fasting glucose, injection site reactions, and alopecia. The three participants with hyperglycaemia all had previously diagnosed diabetes mellitus; none of the participants received a new diagnosis of diabetes mellitus during the study. All AEs were followed-up until abated or stabilised.

**Table 3:** Summary of adverse events in the safety population.

	Lanreotide (n = 22)	Placebo (n = 22)	Risk difference (95% CI)
Any adverse event	22 (100%)	21 (95%)	0·05 (−0·11 to 0·22)
Any serious adverse event	6 (27%)	3 (14%)	0·14 (−0·12 to 0·36)
Hospital admission for planned adenoma resection	2 (9%)	2 (9%)	–
Hospital admission for analysis of chest pain and/or dyspnoea^a^	3 (14%)	0	–
Hospital admission for observation after bike accident	0	1 (5%)	–
Hospital admission for planned ileocecal resection due to complicated Crohn's disease	1 (5%)	0	–
Adverse event leading to treatment discontinuation	4 (18%)	0	0·18 (−0·002 to 0·39)
Deaths	0	0	0 (−0·15 to 0·15)
Adverse event deemed related to study treatment	22 (100%)	10 (45%)	0·55 (0·30 to 0·73)
**Adverse events of special interest**			
Cholelithiasis	0	0	0 (−0·15 to 0·15)
Gastrointestinal disorders	18 (82%)	8 (36%)	0·45 (0·16 to 0·65)
Abdominal pain or discomfort	10 (45%)	2 (9%)	0·36 (0·10 to 0·57)
Increased stool frequency or diarrhoea	16 (73%)	4 (18%)	0·55 (0·25 to 0·72)
Nausea or dyspepsia	8 (36%)	1 (5%)	0·32 (0·08 to 0·53)
Hyperglycaemia (glucose ≥7 mmol/L)	1 (5%)	2 (9%)	−0·05 (−0·24 to 0·14)
Hypoglycaemia	0	0	0 (−0·15 to 0·15)
Impaired fasting glucose (5·7–6·9 mmol/L)	10 (45%)	3 (14%)	0·32 (0·05 to 0·54)
Injection site reaction	12 (55%)	0	0·55 (0·30 to 0·73)
**Other systematically assessed adverse events**			
Age-adjusted IGF-1 SDS below −2·0	4 (18%)	0	0·18 (−0·002 to 0·39)
Alopecia	5 (23%)	0	0·23 (0·03 to 0·43)
Bradycardia	4 (18%)	0	0·18 (−0·002 to 0·39)
Decreased appetite	2 (9%)	1 (5%)	0·05 (−0·14 to 0·24)
Dizziness or light-headedness	2 (9%)	3 (14%)	−0·09 (−0·29 to 0·10)
Fatigue	5 (23%)	3 (14%)	0·09 (−0·14 to 0·32)
Flatulence	3 (14%)	1 (5%)	0·09 (−0·10 to 0·29)
Free thyroxine below lower limit of normal	6 (27%)	1 (5%)	0·23 (0·004 to 0·44)
Headache	7 (32%)	4 (18%)	0·14 (−0·12 to 0·37)
Hot flushes	0	2 (9%)	−0·09 (−0·28 to 0·07)
Insomnia	0	0	0 (−0·15 to 0·15)
Liver function test elevated^b^	4 (18%)	2 (9%)	0·09 (−0·13 to 0·31)
Musculoskeletal pain	1 (5%)	2 (9%)	−0·05 (−0·24 to 0·14)
Unintentional weight loss	2 (9%)	0	0·09 (−0·07 to 0·28)
Visual disturbances	3 (14%)	3 (14%)	0 (−0·22 to 0·22)

Data are n (%) or risk difference (95% confidence interval). An adverse event is defined as any undesirable finding or experience occurring to a participant during the study (between signing of informed consent and up to 30 days after study completion or treatment discontinuation), whether or not considered related to the study or study treatment. A serious adverse event is any untoward medical occurrence that results in death, is life-threatening, requires hospitalisation or prolongation of hospitalisation, results in persistent or significant disability or incapacity, or is a congenital anomaly or birth defect. An overview of all adverse events recorded during the study is provided in the Supplementary Appendix (pp 15–16). IGF-1 SDS, insulin-like growth factor-1 standard deviation score.

a Critical conditions such as pulmonary embolism or myocardial ischemia were ruled out.

b Comprising alanine aminotransferase or gamma-glutamyltransferase >2 times the upper limit of normal or alkaline phosphatase >20 U/L above the upper limit of normal.

There were nine serious adverse events (SAEs), occurring in six (27%) lanreotide group and in three (14%) placebo group participants. None of these were considered treatment-related. Four SAEs concerned planned hospital admissions within one month after withdrawal from the study for transsphenoidal adenoma resection following tumour progression (tumour progression itself was not considered an AE). The other five SAEs concerned hospital admissions for other reasons (Table 3). There were no deaths during treatment.

One event of interest was reported after the predefined observation period, namely symptomatic cholecystolithiasis in a participant treated with lanreotide, which was diagnosed four months after the last injection.^35^

## Discussion

In this 72-week, double-blind and placebo-controlled randomised trial in patients with a ^68^Ga-DOTATATE PET-positive NFPMA, we show no statistically significant difference in change in cranio-caudal tumour diameter after treatment with lanreotide versus placebo. There were also no differences in change in tumour volume or time to tumour progression, nor in change in quality of life. AEs were more frequent in lanreotide-treated participants.

Our study contributes valuable evidence to the ongoing discussion regarding NFPMA management, in which there is a notable lack of randomised controlled trials. While visual disturbances due to optic chiasm compression present an undisputed indication for tumour resection, follow-up strategies for postoperative residual tumours or incidentally found NFPMA without immediate need for decompression are based exclusively on observational and heterogeneous studies.^7^ Moreover, there is a clear unmet need for medical treatment options in patients with NFPMA.^8^

Before initiation of the GALANT trial, a number of open-label and retrospective studies had addressed SSA treatment in NFPMA. Colao et al. summarised the results of 11 case series and uncontrolled studies with a total number of 100 patients.^20^ Tumour shrinkage occurred in 12% of these patients after treatment with octreotide. However, the quality of this evidence is extremely low due to methodological heterogeneity, small number of patients, and short duration of treatment and follow-up. One subsequent prospective study with a mean follow-up of 37 months showed stable residual adenoma size after treatment with octreotide in 81% of 26 patients who were pre-selected through positive SSTR scintigraphy, compared with stability in 47% of 13 untreated patients with negative uptake.^11^ No tumour shrinkage was observed.

In our trial, the mean tumour growth rate in the placebo group (per-protocol analysis, 1·2 mm in 18 months) corresponds to the previously reported average NFPMA growth rate of 0·6–1 mm/year.^6^^,^^29^ We observed significant tumour shrinkage in three (14%) participants in the lanreotide group, which concurs with the data reviewed by Colao et al.^20^ Tumour stability was found in eight (36%) and tumour increase in 11 (50%) of lanreotide-treated participants. Although these results may seem favourable at first sight, at a group level the comparison with placebo-treated participants yielded no statistically significant differences. Our data regarding the percentage of stable disease are in disagreement with the study by Fusco et al., who reported tumour stability in 81% of patients.^11^ We cannot explain this discrepancy on the basis of treatment duration, as this was on average shorter in our study. However, from the methods as presented in their publication it is unclear how tumour size was measured and how stability versus increase/decrease was defined. Only pre-treatment size in millimetres is reported. It is our personal experience that centralised and more precise slice-by-slice measurements can demonstrate significant change in tumour size in a higher number of patients than when solely based on routine radiological reports. Furthermore, it should be emphasised that, unlike the GALANT trial, the study by Fusco et al. did not have a randomised controlled design.

In the GALANT trial, we aimed to optimise therapeutic success rate by randomising only those patients for treatment with *in vivo* evidence of SSTR expression based on ^68^Ga-DOTATATE PET-uptake within the NFPMA. ^68^Ga-DOTATATE has superb affinity for SSTR2, the main target of lanreotide.^22^ We found a remarkable high positive uptake rate (92%) in our population when compared with results from ^111^In-DTPA-octreotide planar scintigraphy or SPECT studies that reported uptake in two thirds of patients with NFPMA.^11^^,^^14^ As discussed in our previous publication regarding the PET-results of a subset of participants, this is most probably due to the superior sensitivity and spatial resolution of ^68^Ga-DOTATATE PET versus ^111^In-DTPA-octreotide imaging.^25^ It also concurs with *in vitro* findings that a total lack of SSTR expression was found in less than 10% of NFPA samples.^13^

There was no relation between the degree of ^68^Ga-DOTATATE uptake and change in tumour size in the lanreotide group (data not shown). While the three participants with significant tumour decrease all had an NFPMA SUV_mean_ >5, high uptake was also observed in participants with significant tumour increase. Whether a higher tracer uptake threshold could select patients with greater potential for treatment response remains uncertain, as the GALANT trial was not powered to address this question. Of note, a discrepancy between intensity of tracer uptake on SSTR scintigraphy and response to SSA treatment has been observed in earlier studies.^11^^,^^20^

With respect to harms, all lanreotide participants experienced one or more AEs that are known and expected side-effects of lanreotide, with a higher rate than placebo participants. Although most reported events were of mild intensity, three participants in the lanreotide group discontinued treatment due to gastrointestinal intolerance, all within the first six months of use. Other trials using lanreotide 120 mg in patients with acromegaly or metastasised neuroendocrine tumours have reported lower withdrawal rates due to treatment-related AEs.^24^^,^^30^ However, these trials were performed in patients with a higher disease burden, which makes comparison difficult.

The GALANT study is the first randomised, double-blind and placebo-controlled phase 3 trial performed in patients with NFPMA. The trial was powered on the change in cranio-caudal tumour diameter, which is clinically the most important criterion in the decision to intervene in order to prevent or relieve optic chiasm compression.^4^ The use of ANCOVA for final analysis (with an observed rho of >0·9 between baseline and end-of-treatment measurement) has increased the power of the study compared to the more conservative t-test based power calculation.^31^ An additional strength in the design of the study is the inclusion of ^68^Ga-DOTATATE PET/CT to objectify SSTR expression *in vivo*. Unbiased tumour measurements were ensured through centralised and standardised assessment by two independent outcome assessors who were additionally blinded for scan chronology. Furthermore, main outcome data were available for all participants.

Several limitations of the study can be noted. A treatment period of 18 months may still be rather short considering the slow natural growth rate of NFPMA, especially if tumour stability is regarded as a reasonable treatment goal.^7^ We cannot exclude that a longer study duration would have increased the power to detect a difference, although it is debatable whether this difference would be clinically relevant, especially in light of risk of side effects and cost of treatment. While some participants without documented growth before study inclusion may have had very slow growing tumours, this cannot be considered a major limitation as it was a controlled trial. Furthermore, the proportion of participants with significant tumour increase was similar among participants with and without documented tumour growth before study treatment (data not shown). There were some chance imbalances in baseline characteristics between treatment groups. Differences in time since diagnosis and the proportion of patients with previous resection may raise concerns about underlying tumour behaviour that may have affected the primary outcome. However, as NFPA can grow undetected for a long time and are not seldom incidental findings, time since diagnosis is not necessarily related to tumour size or growth. Indeed, in our data there was no statistically significant correlation within treatment groups between time since diagnosis and either baseline cranio-caudal diameter or post-treatment change (data not shown). A post-hoc sensitivity ANCOVA for the primary outcome with inclusion of time since diagnosis as additional continuous covariate did not change our results in a significant manner. With respect to previous resection, several studies have shown that the proportion of patients with tumour progression over time is similar for NFPMA followed-up conservatively and postoperative remnants not treated with adjuvant radiotherapy (both close to 50% at 5 years).^4^^,^^6^ Our data are consistent with these earlier observations: of the participants with previous resection, 11/24 (46%) experienced significant tumour size increase, while of those with unoperated tumours, 8/20 (40%) had significant tumour size increase. With regard to treatment discontinuation, the differential dropout rate of 41% for lanreotide versus 14% for placebo may have introduced bias. However, the imbalance in dropout was largely due to (gastrointestinal) AEs in the lanreotide group. As the AEs leading to dropout were deemed unrelated to tumour size, there is less risk of bias due to data being missing-not-at-random. In addition, we obtained post-baseline outcome data for all participants at time of treatment discontinuation, further reducing the risk of bias. In light of our power calculation, 16 lanreotide and 19 placebo participants completed at least 16 out of 18 injections and thus (near-)completed the trial, ensuring adequate power. Furthermore, results were robust to sensitivity analyses for missing data. We therefore consider our results to be reliable. As a final note, the frequent occurrence of lanreotide-related AEs may have compromised blinding of participants. However, this is unlikely to have affected tumour growth rate.

Despite advances in surgical techniques, complete resection is not achieved in the majority of patients with NFPMA, and long-term follow-up is required due to the high regrowth rate.^5^^,^^6^ An effective medical treatment modality would thus be of great value. The results of our study do not support the use of lanreotide to reduce NFPMA growth. Alternative medical treatment strategies may still be considered. For example, a recent historical cohort analysis with a mean follow-up of 8·8 years showed residual adenoma stabilisation or shrinkage in up to 87% of 79 patients treated with dopamine receptor agonists (bromocriptine or cabergoline), versus tumour control in 47% of 60 untreated control patients.^9^ A further randomised open-label trial found tumour decrease in 28·8% of 59 participants over two years of treatment with cabergoline versus 10·5% in 57 participants without intervention.^10^ Despite these encouraging data, efficacy of dopamine receptor agonists has not been demonstrated in a double-blind and placebo-controlled randomised trial.

In conclusion, the GALANT trial shows that, compared with placebo, lanreotide treatment for 72 weeks does not reduce tumour size or halt tumour growth in NFPMA with positive ^68^Ga-DOTATATE PET-uptake. Based on our findings, we do not recommend routine use of SSAs in patients with NFPMA.

## Contributors

TMB, MLD, JB, CBLMM, MPMS, MWTT, EF, and PHB participated in the design of the study. TMB coordinated the study. EF supervised the study in name of the sponsor (Amsterdam UMC location AMC). EF, MLD, MPMS, and AMP were principal investigators at the participating study centres. MLD, JH, AMP, NRB, SS, RGV, EF, and PHB assisted with the referral and inclusion of patients. TMB established informed consent, arranged study planning, and was responsible for data collection. TMB performed PET/MRI analysis, under supervision of JB. AJW and JMV performed MRI measurements, under supervision of TMB and CBLMM. TMB verified the data and performed the statistical analyses, assisted by MWTT. TMB, MLD, MWTT, EF, and PHB interpreted the results. TMB, EF, and PHB had access to all of the data in the study and take responsibility for the integrity of the data and the accuracy of the analyses. TMB, MLD, EF, and PHB wrote the manuscript. All authors reviewed and approved the final manuscript.

## Data sharing statement

The study protocol has been published previously.^23^ The study protocol document and amended versions as approved by the ethics committee, as well as the statistical analysis plan and monitoring plan will be available upon request after publication. The datasets with de-identified participant data generated and/or analysed during the current study can be requested by contacting the corresponding author upon approval of a proposal and with a signed data access agreement.

## Declaration of interests

MLD was independent chair and organiser of the Dutch Neuro-endocrine Symposium 2021, funded by Ipsen Farmaceutica BV. JH was invited lecturer at this same symposium. CBLMM has received several unrelated grants paid to the institution in the past 36 months, namely from CVON/Dutch Heart Foundation, the European Commission, Healthcare Evaluation Netherlands, TWIIN Foundation, and Stryker; and further owns a minority interest in Nicolab.

No other potential conflicts of interest have been declared.

## Appendix A Supplementary data

Supplementary Appendix

Study protocol GALANT trial

Statistical Analysis Plan GALANT trial

Data Management Plan GALANT

DELTA2 checklist_GALANT

CONSORT 2008 abstract checklist
