# Supplementary material for: Lanreotide versus placebo for tumour reduction in patients with a 68Ga-DOTATATE PET-positive, clinically non-functioning pituitary macroadenoma (GALANT study): a randomised, multicentre, phase 3 trial with blinded outcome assessment
Source: Lancet Reg Health Eur. 2024 May 13;42:100923. doi: 10.1016/j.lanepe.2024.100923 (PMC11281922; doi:10.1016/j.lanepe.2024.100923)
Supplement: Supplementary Appendix [file mmc1.pdf]

Supplementary Appendix

Lanreotide versus placebo for tumour reduction in patients with a ^{68}Ga-DOTATATE PET-positive, clinically non-functioning pituitary macroadenoma (GALANT study): a randomised, multicentre, phase 3 trial with blinded outcome assessment

Tessel M. Boertien, Madeleine L. Drent, Jan Booij, Charles B. L. M. Majoie, Marcel P. M. Stokkel, Jantien Hoogmoed, Alberto M. Pereira, Nienke R. Biermasz, Suat Simsek PhD, Ronald Groote Veldman, Annick J. Weterings, Juan M. Vink, Michael W.T. Tanck, Eric Fliers, Peter H. Bisschop

Table of Contents

Systematic literature search strategy 2
Protocol versions and substantial amendments 5
Protocol deviations 6
Overview of participants with deviating or missing week-24/week-72 MRI outcome data 7
Statistical methods 8
References 11
Supplementary Tables 12
Table S1. Clinical characteristics and PET results of all included participants 12
Table S2. Primary and secondary tumour size outcomes in the per-protocol population 12
Table S3. Result overview of the main, additional and sensitivity analyses for the primary outcome 13
Table S4. Change in quality of life based on SF-36 component scores 14
Table S5. All recorded adverse events per system organ class in the safety population 15
Supplementary Figures 17
Figure S1. Individual change in tumour size from baseline to end-of-study MRI 17
Figure S2. Time to progression based on tumour volume or cranio-caudal size 18
Figure S3. SF-36 component score spider charts 19

Systematic literature search strategy

Database: **Ovid MEDLINE(R) and Epub Ahead of Print, In-Process & Other Non-Indexed Citations and Daily** 1946 to June 01, 2020

Search Strategy: **2020-06-02**

#	Searches	Results
1	adenoma, chromophobe/	1445
2	(NFPA or NFPAs or NFPT or NFPTs or NFMA or NFMAs or NFA or NFAs or CNPA or CNPAs or CNPT or CNPTs).tw,kf. and ((pituit* or hypophys* or sella).mp. or exp hypothalamic neoplasms/)	545
3	((NF not NF-I) adj6 (pituitary or hypophys*) adj2 (adenom* or microadenom* or macroadenom*)).tw.	17
4	(NF adj (adenom* or microadenom* or macroadenom* or OMA or OMas or pituit* or hypofys* or PA or PAs or PT or PTs or MA or MAs)).tw,kf.	36
5	(chromophob* or gonadotropinom* or gonado-tropinom*).tw,kf.	2735
6	((gonadotrope or gonado-trope or gonadotroph* or gonado-troph* or gonadotrop?ic or gonado-trop?ic or null-cell* or null*cell*) adj3 (pituit* or hypophys* or adenom* or microadenom* or macroadenom* or tumo?r*)).tw,kf.	2628
**7**	**or/1-6 [NFPA-I]**	**6776**
8	exp pituitary neoplasms/	27458
9	(exp pituitary gland/ or (pituit* or hypophys* or sella or PA or PAs).tw,kf.) and (exp adenoma/ or (adenom* or macroadenom* or microadenom* or neoplas* or tum?or*).tw,kf.)	38694
**10**	**or/8-9 [pituitary adenoma]**	**48030**
11	adenoma, oxyphilic/	2073
12	negative results/	89
13	(nonfunct* or non-funct* or functionles* or non-secret* or nonsecret* or "not-secreting" or no?-hormone-secret* or nonproduc* or non-produc* or non-hormone-produc* or non-PRL-produc* or non-prolactin*-produc* or PRL-negativ* or "not-producing" or no-pituitary-hormone-excess* or no-hormone-excess* or silent).tw,kf.	69749
14	(inactive adj2 (tumo?r* or adenom* or macroadenom* or microadenom* or pituit* or hypophys* or hormon* or clinically)).tw,kf.	1120
15	((immunonegativ* or immuno-negativ*) adj3 (hormone* or adenom* or microadenom* or macroadenom* or pituit* or hypophys*)).tw,kf.	31
16	(negativ* adj2 (hormon* or stain* or immunostain*)).tw,kf.	13840
17	((((alpha* or alfa*) adj2 (subunit* or sub-unit*)) or alpha-SU or alfa-SU) adj4 (secret* or hypersecret* or producing or releasing)).tw,kf.	375
18	((enhanced or elevated or increased) adj2 (((alpha* or alfa*) adj2 (subunit* or sub-unit*)) or alpha-SU or alfa-SU)).tw,kf.	422
19	oncocytom*.tw,kf.	2664
20	(oxyphil* adj3 (adenom* or microadenom* or macroadenom* or pituit* or hypophys*)).tw,kf.	193
**21**	**or/11-20 [nonfunctional]**	**88944**
**22**	**10 and 21 [NFPA-II]**	**3755**
**23**	**7 or 22 [NFPA I & II]**	**9553**
24	exp dopamine agonists/ or exp ergolines/ or quinolines/ or aminoquinolines/	70465
25	exp receptors, dopamine/ag, ai, tu [agonists, antagonists & inhibitors, therapeutic use]	3757
26	((dopamin* or DA or D2 or D-2) adj3 agonist*).tw,kf.	17140
27	((dopamine or DA) adj3 receptor* adj2 (therap* or stimulator*)).tw,kf.	95
28	((dopaminerg* adj3 (agent* or drug or drugs or medicin* or therap* or treatm*)) or dopaminergics).tw,kf.	4917
29	(ergolin* or nonergot or non-ergot).tw,kf.	860

30	(bromo*r#ptin* or ergo*r#ptin* or Parlodel or CB-154 or CB154 or pergolid* or cabergolin* or Cabaser* or Cabest or FCE-21336 or FCE21336 or Galastop or Dostinex or quinagolid* or Norprolac or CV-205-502 or CV205-502).tw,kf.	9994
31	somatostatin/ag, aa, tu [agonists, analogs & derivatives, therapeutic use]	5618
32	octreotide/	7545
33	(lanreotide or angiopeptin or pasireotide).rn.	1110
34	((somatostatin* or SS* or SRIF) adj6 (analog* or agonist* or derivativ*)).tw,kf.	9699
35	(oc*reotid* or Sandostatin* or "201995" or "201-995" or lanreotid* or angiopeptin* or Somatulin* or Ipstyl or BIM23014* or BIM-23014* or DC13-116 or DC-13-116 or pasireotid* or Signifor or som-230 or som230).tw,kf.	9611
**36**	**or/24-35 [DA, SS-analogues]**	**101121**
**37**	**23 and 36 [NFPA + DA & SS-analogues]**	**608**
**38**	**exp animals/ not humans/ [animal filter]**	**4703271**
**39**	**37 not 38 [human NFPA + DA & SS-analogues]**	**581**
**40**	**remove duplicates from 39 [human NFPA + DA & SS-analogues]**	**580**

Database: **Embase Classic+Embase** 1947 to 2020 June 01
Search Strategy: **2020-06-02**

#	Searches	Results
1	nonfunctioning pituitary adenoma/	1097
2	chromophobe adenoma/ or gonadotroph adenoma/ or null cell adenoma/	1998
3	(NFPA or NFPAs or NFPT or NFPTs or NFMA or NFMAs or NFA or NFAs or CNPA or CNPAs or CNPT or CNPTs).tw,kw. and ((pituit* or hypophys* or sella).mp. or exp hypophysis tumor/)	767
4	((NF not NF-I) adj6 (pituitary or hypophys*) adj2 (adenom* or microadenom* or macroadenom*)).tw.	24
5	(NF adj (adenom* or microadenom* or macroadenom* or OMA or OMas or pituit* or hypofys* or PA or PAs or PT or PTs or MA or MAs)).tw,kw.	53
6	(chromophob* or gonadotropinom* or gonado-tropinom*).tw,kw.	5095
7	((gonadotrope or gonado-trope or gonadotroph* or gonado-troph* or gonadotrop?ic or gonado-trop?ic or null-cell* or null*cell*) adj3 (pituit* or hypophys* or adenom* or microadenom* or macroadenom* or tumo?r*)).tw,kw.	3966
**8**	**or/1-7 [NFPA-I]**	**11134**
9	exp hypophysis tumor/	43807
10	(hypophysis/ or (pituit* or hypophys* or sella or PA or PAs).tw,kw.) and (exp adenoma/ or (adenom* or macroadenom* or microadenom* or neoplas* or tum?or*).tw,kw.)	54335
**11**	**or/9-10 [pituitary adenoma]**	**70357**
12	oncocytoma/	5210
13	null result/	1563
14	(nonfunct* or non-funct* or functionles* or non-secret* or nonsecret* or "not-secreting" or no?-hormone-secret* or nonproduc* or non-produc* or non-hormone-produc* or non-RPL-produc* or non-prolactin*-produc* or PRL-negativ* or "not-producing" or no-pituitary-hormone-excess* or no-hormone-excess* or silent).tw,kw.	95702
15	(inactive adj2 (tumo?r* or adenom* or macroadenom* or microadenom* or pituit* or hypophys* or hormon* or clinically)).tw,kw.	1674
16	((immunonegativ* or immuno-negativ*) adj3 (hormone* or adenom* or microadenom* or macroadenom* or pituit* or hypophys*)).tw,kw.	37
17	(negativ* adj2 (hormon* or stain* or immunostain*)).tw,kw.	19391
18	((((alpha* or alfa*) adj2 (subunit* or sub-unit*)) or alpha-SU or alfa-SU) adj4 (secret* or hypersecret* or producing or releasing)).tw,kw.	405
19	((enhanced or elevated or increased) adj2 (((alpha* or alfa*) adj2 (subunit* or sub-unit*)) or alpha-SU or alfa-SU)).tw,kw.	459
20	oncocytom*.tw,kw.	3992
21	(oxyphil* adj3 (adenom* or microadenom* or macroadenom* or pituit* or hypophys*)).tw,kw.	263

**22**	**or/12-21 [nonfunctional]**	**124839**
**23**	**11 and 22 [NFPA-II]**	**5359**
**24**	**8 or 23 [NFPA I & II]**	**14677**
25	dopamine receptor stimulating agent/ or bromocriptine/ or bromocriptine mesilate/ or cabergoline/ or dopamine 1 receptor stimulating agent/ or dopamine 2 receptor stimulating agent/ or dopamine 3 receptor stimulating agent/ or pergolide/ or quinagolide/	43035
26	((dopamin* or DA or D2 or D-2) adj3 agonist*).tw,kw.	22464
27	((dopamine or DA) adj3 receptor* adj2 (therap* or stimulator*)).tw,kw.	144
28	((dopaminerg* adj3 (agent* or drug or drugs or medicin* or therap* or treatm*)) or dopaminergics).tw,kw.	7365
29	(ergolin* or nonergot or non-ergot).tw,kw.	1240
30	(bromo*r#ptin* or ergo*r#ptin* or Parlodel or CB-154 or CB154 or pergolid* or cabergolin* or Cabaser* or Cabest or FCE-21336 or FCE21336 or Galastop or Dostinex or quinagolid* or Norprolac or CV-205-502 or CV205-502).tw,kw.	14429
31	somatostatin derivative/ or angiopeptin/ or octreotide/ or pasireotide/	28291
32	((somatostatin* or SS* or SRIF) adj6 (analog* or agonist* or derivativ*)).tw,kw.	13793
33	(oc*reotid* or Sandostatin* or "201995" or "201-995" or lanreotid* or angiopeptin* or Somatulin* or Ipstyl or BIM23014* or BIM-23014* or DC13-116 or DC-13-116 or pasireotid* or Signifor or som-230 or som230).tw,kw.	16335
**34**	**or/25-33 [DA or SS-analogues]**	**90443**
**35**	**24 and 34 [NFPA & DA or SS-analogues]**	**1052**
**36**	**(animal/ or animal experiment/ or animal model/ or nonhuman/ or rat/ or mouse/) not human/**	**6915046**
**37**	**35 not 36 [human NFPA & DA or SS-analogues]**	**1025**
**38**	**remove duplicates from 37**	**1016**

Database: **CENTRAL**
Search run: **2020-06-02**

ID	Search	Hits
#1	(NFPA or NFPAs or NFPT or NFPTs or NFMA or NFMAs or NFA or NFAs or CNPA or CNPAs or CNPT or CNPTs):ti,ab,kw. and ((pituit* or hypophys* or sella) or [mh "Hypothalamic Neoplasms"])	12
#2	(NF near/6 (pituitary or hypophys*))	0
#3	(NF next (adenom* or microadenom* or macroadenom* or OMA or OMas or pituit* or hypofys* or PA or PAs or PT or PTs or MA or MAs)):ti,ab	1
#4	chromophob* or gonadotropinom* or gonado-tropinom*	47
#5	((gonadotrope or gonado-trope or gonadotroph* or gonado-troph* or gonadotrop*ic or gonado-trop*ic or (null next cell*) or null*cell*) near/3 (pituit* or hypophys* or adenom* or microadenom* or macroadenom* or tumo*r*)):ti,ab,kw	37
**#6**	**{OR #1-#5}**	**96**
#7	[mh "Pituitary Neoplasms"]	208
#8	((pituit* or hypophys* or sella):ti,ab,kw or (PA or PAs):ti,ab) and (adenom* or macroadenom* or microadenom* or neoplas* or tumo*r*):ti,ab,kw	1759
**#9**	**#7 or #8**	**1769**
#10	(nonfunct* or non-funct* or functionles* or non-secret* or nonsecret* or "not-secreting" or "not-hormone-secreting" or nonproduc* or non-produc* or non-hormone-produc* or non-RPL-produc* or non-prolactin*-produc* or PRL-negativ* or "not-producing" or no-hormone-excess* or silent):ti,ab,kw	2420
#11	(inactive near/2 (tumo*r* or adenom* or macroadenom* or microadenom* or pituit* or hypophys* or hormon* or clinically)):ti,ab,kw	42
#12	((immunonegativ* or immuno-negativ*) near/3 (hormone* or adenom* or microadenom* or macroadenom* or pituit* or hypophys*))	0
#13	(negativ* near/2 (hormon* or stain* or immunostain*)):ti,ab	367

#14	((((alpha* or alfa*) near/2 (subunit* or sub-unit*)) or alpha-SU or alfa-SU) near/4 (secret* or hypersecret* or producing or releasing)):ti,ab,kw	3
#15	((enhanced or elevated or increased) near/2 (((alpha* or alfa*) near/2 (subunit* or sub-unit*)) or alpha-SU or alfa-SU)):ti,ab	2
#16	oncocytom*	12
#17	(oxyphil* near/3 (adenom* or microadenom* or macroadenom* or pituit* or hypophys*))	5
**#18**	**{OR #10-#17}**	**2849**
**#19**	**#9 and #18**	**46**
**#20**	**#6 or #19**	**129**
#21	[mh "dopamine agonists"] or [mh ergolines] or [mh quinolines] or [mh aminoquinolines]	10958
#22	[mh "Receptors, Dopamine"/AG,AI,TU]	61
#23	((dopamin* or DA or D2 or D-2) near/3 agonist*):ti,ab,kw	1895
#24	((dopamine or DA) near/3 receptor* near/2 (therap* or stimulator*)):ti,ab,kw	135
#25	((dopaminerg* near/3 (agent* or drug or drugs or medicin* or therap* or treatm*)) or dopaminergics):ti,ab,kw	695
#26	(ergolin* or nonergot or non-ergot):ti,ab,kw	315
#27	bromocr*ptin* or ergocr*ptin* or bromokr*ptin* or ergokr*ptin* or Parlodel or CB-154 or CB154 or pergolid* or cabergolin* or Cabaser* or CAB or CABEST or FCE-21336 or FCE21336 or Galastop or Dostinex or quinagolid* or Norprolac or (CV205 NEXT 502) or (CV NEXT 205 NEXT 502):ti,ab,kw	2099
**#28**	[mh Somatostatin/ag,aa,tu]	321
#29	((somatostatin* or SS* or SRIF) near/6 (analog* or agonist* or derivativ*)):ti,ab,kw	1235
#30	oc?reotid* or Sandostatin* or "201995" or "201-995" or lanreotid* or angiopeptin* or Somatulin* or Ipstyl or BIM23014* or BIM-23014* or DC13-116 or DC-13-116 or pasireotid* or Signifor or som-230 or som230	1976
#31	**{OR #21-#30}**	**16299**
**#32**	**#20 and #31**	**14**
**#33**	**#32 in Trials**	**14**
**#34**	#32 in Cochrane Reviews	**0**

Protocol versions and substantial amendments

- V2·0, May 21st 2015: first approved protocol version.
- V3·0, October 13th 2016:
  - Leiden University Medical Centre added as participating centre to achieve target recruitment goal
  - Ability to perform the ^{68}Ga-DOTATATE PET/CT at Amsterdam UMC location AMC
  - Possibility for participants to have study injections administered at home by trained nurses of a specialised homecare company (Eurocept Homecare).
- V4·0, August 31st 2017:
  - Clearer definition of exclusion criterion concerning dopamine receptor agonist use: “Use of dopamine receptor agonists” was modified to “Use of dopamine receptor agonist in the past 6 months”. Inclusions up to this amendment were not affected by the modification.
- V5·0, November 30th 2018 (final protocol version, used for publication[1]):
  - Increased sample size to 22 participants per treatment group to account for an observed overall dropout rate of ~25%.
  - More detailed statistical analysis section.

All amendments were approved centrally by the ethics committee of Amsterdam UMC location AMC (number METC 2015_103) and the Central Committee on Research Involving Human Subjects (number NL52821.018.15), and locally by the boards of directors of the participating centres.

Protocol deviations

The pre-randomisation timeline as presented in the protocol (page 22) proved difficult to adhere to, due to the short two-week window between screening, which included planning and evaluation of the ^{68}Ga-DOTATATE PET-CT, and start of study treatment. This difficulty was discussed during a monitoring visit and relaxation of the screening period was recorded on a protocol deviation form at study level, without the need to submit an official protocol amendment. The published version of the final protocol shows the relaxed schedule without a specific time window for the screening phase.[1] Allowance for more flexible scheduling of study injections and visits to accommodate participants, aiming for a maximum of seven days before or after an originally scheduled appointment, was discussed at the same monitoring visit and recorded on a separate protocol deviation form at study level.

The preferred three month limit between baseline pituitary MRI and start of study treatment was exceeded in ten participants (five in the lanreotide group and five in the placebo group), with a range from six to 46 days. The median (IQR) time between baseline MRI and first study injection was 57 (8–92) days in the lanreotide group and 10 (0–93) days in the placebo group. The reasons for these deviations were almost all logistical in nature, where delays in e.g. ^{68}Ga-DOTATATE PET-CT imaging or injection planning resulted in a longer than expected time between baseline MRI and the first study injection, without the possibility of repeating the MRI.

Deviations in the study schedule exceeding seven days occurred for six injections, two week-24 visits and three week-72 visits. Deviations were mainly due to planning outside of holidays. Week-24 MRI was missing in one placebo participant due to cancellation by the participant without rescheduling of the appointment.

<u>Deviations related to the COVID-19 pandemic</u>

There were no important trial modifications following the COVID-19 pandemic. In two participants in the placebo group, the MRI of the week-24 visit was postponed with respectively two and five months due to COVID-19 restrictions regarding MRI quota. The remainder of the week-24 assessment for these participants was conducted according to protocol on the originally scheduled date.

Overview of participants with deviating or missing week-24/week-72 MRI outcome data

Study number	Study completed	Final injection before end-of-treatment MRI	MRI week-24	MRI week-72	MRI performed outside of study schedule	Reason for deviation/incomplete data	Assumed missing mechanism	Estimation of impact on study outcome
**LANREOTIDE**								
2	No	16	✓	Missing	Yes, after 16th injection	Treatment discontinuation following diagnosis of de novo Cushing disease (history of resected silent corticotroph adenoma without earlier signs of hypercortisolism)	MCAR – dropout unrelated to study or study outcome	Minimal impact, study near-completed at time of end-of-treatment MRI
3	No	9	✓	Missing	Yes, after 9th injection	Treatment discontinuation to undergo elective tumour resection following tumour progression on week-24 MRI with increased optic chiasm compression but no visual compromise	MAR plausible given baseline tumour size	Possible impact
12	No	6	✓	Missing	No	Treatment discontinuation due to gastrointestinal AEs	MAR plausible given treatment – AE not related to tumour size	Possible impact
16	No	6	✓	Missing	No	Treatment discontinuation to undergo tumour resection following visual disturbances and new objectified visual field defects, week-24 MRI showed subtle tumour progression	MAR plausible given baseline tumour size	Possible impact
20	No	16	✓	Missing	Yes, after 16th injection	Treatment discontinuation to undergo tumour resection following visual disturbances and new objectified visual field defects, additional MRI showed tumour progression	MAR plausible given baseline tumour size	Minimal impact, study near-completed at time of end-of-treatment MRI
23	No	4	Missing	Missing	Yes, after 4th injection	Treatment discontinuation due to AEs of light-headedness and parosmia	MAR plausible given treatment – AE not related to tumour size	Possible impact
25	No	2	Missing	Missing	Yes, after 2nd injection	Treatment discontinuation due to severe gastrointestinal AEs with weight loss	MAR plausible given treatment – AE not related to tumour size	Possible impact
28	No	16	✓	Missing	Yes, after 16th injection	Treatment discontinuation after additional MRI was performed for nonspecific visual complaints. MRI showed no relevant tumour progression or reason for intervention, but repeat imaging within 3 months was deemed not indicated	MCAR – study not completed due to logistic reasons	Minimal impact, study near-completed at time of end-of-treatment MRI
34	No	3	Missing	Missing	Yes, after 3rd injection	Treatment discontinuation due to gastrointestinal AEs	MAR plausible given treatment – AE not related to tumour size	Possible impact
**PLACEBO**								
14	No	6	✓	Missing	No	Treatment discontinuation to start dopamine receptor agonist treatment following subtle tumour progression on week-24 MRI in patient with unrelated visual complaints	MAR plausible given baseline tumour size	Possible impact
18*	Yes	18	Missing	✓	No	Week-24 MRI cancelled by participant without rescheduling	MCAR – MRI cancellation unrelated to study	No impact, week-72 MRI performed
21	No	6	✓	Missing	No	Treatment discontinuation to undergo elective tumour resection following tumour progression on week-24 MRI with new optic chiasm compression but no visual compromise; pathology results revealed a pituicytoma	MAR plausible given baseline tumour size	Possible impact
40	No	12	✓	Missing	Yes, after 12th injection	Treatment discontinuation to undergo elective tumour resection following subtle tumour progression on week-24 MRI without optic chiasm compression	MAR plausible given baseline tumour size	Possible impact
42*	Yes	18	Missing	✓	Yes, after 8th injection	Week-24 MRI postponed due to COVID-19 related restrictions, week-72 MRI took place as scheduled	MCAR – MRI rescheduling unrelated to study	No impact, week-72 MRI performed
43*	Yes	18	Missing	✓	Yes, after 12th injection	Week-24 MRI postponed due to COVID-19 related restrictions, week-72 MRI took place as scheduled	MCAR – MRI rescheduling unrelated to study	No impact, week-72 MRI performed

According to protocol, week-24 MRI was planned after the 6th injection, and week-72 MRI was planned after the 18th injection. Out of 88 planned post-baseline MRIs in 44 participants, 18 (20%) MRIs in 15 participants were not performed according to protocol. There were 10 (additional) MRIs performed outside of study schedule, either as a premature end-of-treatment assessment (n=8) or due to postponement of the MRI appointment (n=2). AEs=adverse events. *These participants were included in the per-protocol analysis, as treatment was completed and week-72 MRI performed.

Statistical methods

Distribution of tumour size data

Distribution of baseline and end-of-treatment cranio-caudal diameter had slight positive skew, and the median and interquartile range are reported. Change in cranio-caudal diameter from baseline to end-of-treatment was normally distributed. For the primary outcome, parametric tests without transformation of baseline or end-of-treatment values could be applied provided that assumptions were met (eg, linearity, homogeneity, homoscedasticity and normality of residuals).

Distribution of baseline and end-of-treatment tumour volume had more pronounced positive skewness, and the change from baseline also had skewed distribution. Therefore, tumour values were natural log-transformed before analysis. The ANCOVA-adjusted log-transformed mean difference in the change from baseline versus placebo was interpreted as follows: back-transformation of the mean difference of two natural log-transformed samples mean1 and mean2 via the natural exponential function results in $e^{mean1-mean2}$, which is equal to e^{mean1}/e^{mean2}. Therefore, the back-transformed mean difference value signifies the ratio of group means (ie, mean1 has a certain percentage higher/lower value compared to mean2) and not the absolute difference.[2] This value was then used to correctly calculate the mean difference versus placebo in mm^3 using the back-transformed estimated marginal mean of the placebo group. The back-transformed 95% confidence interval values were interpreted and calculated in a similar way.

In the per-protocol population (including only those participants who completed study treatment with all 18 injections and underwent week-72 MRI), both baseline and end-of-treatment cranio-caudal diameter as baseline and end-of-treatment tumour were more normally distributed. No transformations were needed for analysis.

Missing week-72 MRI outcome data: assumed missing mechanism

Missing data patterns and relation between missing data and other variables were explored via Missing Value Analysis, group comparisons and logistic regression analysis in SPSS (version 28).[3,4] Group comparisons showed no apparent or statistically significant difference in age at enrolment or sex between participants who completed treatment ('completers') and those who discontinued treatment ('dropouts'). There was an apparent difference in baseline cranio-caudal diameter, with a larger size for participants who eventually dropped out (mean [SD] completers 16·5 [3·0] mm versus dropouts 19·8 [9·0] mm). Although this difference was statistically non-significant (mean difference 3·3 mm, Welch test p=0·24), it was deemed relevant.

Through logistic regression we assessed whether the probability of dropout was related to baseline cranio-caudal diameter, treatment group, and/or post-baseline significant increase in tumour size (of either cranio-caudal diameter or tumour volume). This showed that only treatment was a significant predictor of dropout (p=0·04), indicating higher chance of dropout due to the interventional treatment. This was mostly due to a higher occurrence of adverse events (AEs) in the lanreotide group, with four AE-related dropouts in the lanreotide group versus zero in the placebo group. The AEs that led to dropout were deemed not related to tumour size: gastrointestinal complaints in three participants and light-headedness and parosmia in one participant. For the six participants who dropped out due to indication for tumour-related intervention (ie, 'tumour progression', n=3 in each group), baseline cranio-caudal diameter was a significant predictor of dropout (p=0·01).

The relation of dropout with treatment group and (partly) with baseline size rejected data missing completely at random (MCAR) for most missings. However, as treatment group and baseline size were both included as covariates in the outcome model, and as the AEs leading to dropout were deemed unrelated to tumour size, the assumption of data missing-at-random (MAR) remained plausible.

Multiple imputation of missing week-72 MRI outcome data

For the supplementary efficacy analysis of the primary outcome change in cranio-caudal tumour diameter and the secondary outcome change in tumour volume, ANCOVA was performed after imputation of missing week-72 data.[3,5] This was done separately for cranio-caudal diameter and tumour volume data via univariate multiple imputation. Tumour volume values were natural log-transformed before imputation.

With the aim of only imputing missing week-72 MRI outcome data, under the MAR assumption, imputations from the Bayesian paradigm were obtained using a regression model and uninformative priors. Predictive mean matching was considered inadequate due to insufficient nearby donors. Multiple imputation was performed separately for each treatment arm (which implicitly allows for interactions between all included covariates and treatment group).[5] To facilitate a monotone missing pattern and reduce the risk of collinearity and/or overfitting due to multiple highly

correlated measurements, a separate variable was constructed to contain data from the last MRI performed before the week-72 visit for each participant. This variable thus contained MRI-data from varying time-points, depending on treatment completion, timing of dropout, and (for two participants) different timing of the week-24 MRI. Note that as a consequence, the imputation model did not include week-24 data from five participants who underwent a premature end-of-treatment MRI past the week-24 visit. The new variable was entered as predictor variable next to size at baseline and at week-72. For the one placebo participant in whom only baseline and week-72 MRI were performed (week-24 MRI missing, assumed MCAR), week-24 MRI tumour size values were simply imputed as the average of baseline and week-72 to ensure the monotone missing pattern without impacting imputation results. The number of imputed datasets was set at 27, corresponding to 27% of missing week-72 MRI data (12/44 participants).[6]

Precision of the multiple imputation was checked via the fraction of missing information (FMI), the relative increase in variance due to nonresponse (RIV), and the relative efficiency (RE).[3] An FMI divided by the number of imputed datasets of ≤0.01 (ie, 1%) is regarded as adequate precision with low variability between the imputed datasets. Lower RIV values indicate less increase in sampling variance due to the missing data. The RE provides information on the precision of the parameter estimate as the standard error of a regression coefficient, where higher values are better. Precision checks were satisfactory for multiple imputation of both cranio-caudal diameter and tumour volume.

After imputation, the ANCOVA as specified for the main analysis was performed in each imputed dataset, with end-of-treatment measurement as dependent variable, baseline measurement as continuous covariate, and treatment as categorical covariate. Assumptions were checked and results pooled following Rubin's rules. Results for the primary outcome are included in supplementary table 3; for secondary outcome change in tumour volume, the adjusted mean difference versus placebo was +75 mm^3 (95% CI –499 to 583 mm^3), p=0·79.

<u>Linear mixed effects model</u>
Efficacy for the primary outcome was additionally assessed via a linear mixed effects (LME) model, which makes full use of information from repeated MRI measurements taken at varying time-points and implicitly imputes missing data under the MAR assumption.[7] It provides flexibility to accommodate irregular observation intervals across participants as well as sparse data at specific time-points, caused by premature assessments in case of treatment discontinuation. The original, prespecified model in the statistical analysis plan included treatment group, measurement time (in the form of the number of received injections after which MRI measurement was obtained), and group-by-time interaction as fixed effects, plus a by-subject random intercept as random effect. Fitting of a random slope was not considered feasible by design due to too few post-baseline observations. The final model was modified post-hoc to include the additional fixed effects of baseline cranio-caudal diameter and baseline diameter-by-time interaction to correctly adjust for baseline size; the outcome/response vector was adjusted to only contain post-baseline measurements.[8] As an additional post-hoc optimisation, a residual correlation matrix was specified to address possible residual serial autocorrelation not sufficiently accounted for by the random intercept.[9] The continuous time first-order autoregressive correlation structure was deemed most suitable considering the use of a continuous time variable with unequally spaced observations and a plausible assumption of decreasing autocorrelation over time.

The LME model was fitted in R using the *lme* function of the *nlme* package, which allows for modelling of correlation structures[10]:

```
nlme::lme(cc_size ~ group*MRI_time_inj + cc_base_*MRI_time_inj,
      random = ~ 1 | participant_id,
      correlation = corCAR1(form = ~ MRI_time_inj | participant_id),
      data = data, method = "REML")
```

Where `cc_size` is post-baseline cranio-caudal diameter, `group` is treatment group (0=placebo and 1=lanreotide), `MRI_time_inj` is the continuous time variable based on number of received injections after which measurement was obtained, `cc_base` is cranio-caudal diameter at baseline, and `participant_id` is the participant. The model includes the fixed effects of group, time, group-by-time interaction, baseline size, and baseline size-by-time interaction. A by-subject random intercept is specified through: `random = ~ 1 | participant_id`. A continuous time first-order autoregressive correlation structure is specified through: `correlation = corCAR1(form = ~ MRI_time_inj | participant_id)`. The model was fitted using restricted maximum likelihood estimation of parameters (`method = "REML"`). Treatment effect was subsequently estimated as the contrast between treatment groups at the final measurement time (after the 18th injection, corresponding to week-72) using the *emmeans* package, based on least-square means with specification of the Satterthwaite method for

approximation of the denominator degrees of freedom. This yielded an adjusted mean difference versus placebo of –0·13 mm (95% CI –1·29 to 1·03 mm).

To relax the assumption of linear time trends, data were also fitted post-hoc using a mixed model for repeated measurements (MMRM), with time as a categorical variable and an unstructured covariance matrix to model within-patient residual errors.[11,12] This, however, required 'simplification' of post-baseline time data to a factor with only 4 levels: time1=measurement obtained before week-24 visit, time2=week-24 visit, time3=measurement obtained between week-24 and week-72 visit, and time4=week-72 (end) visit. The MMRM was fitted in R using the *mmrm* function of the *mmrm* package[13]:

```
mmrm::mmrm(cc_size ~ group*MRI_time_factor + cc_base*MRI_time_factor
       + us(MRI_time_factor|participant_id),
       data=data, reml = TRUE, method = "Satterthwaite")
```

Where `cc_size` is post-baseline cranio-caudal diameter, `group` is treatment group (0=placebo and 1=lanreotide), `MRI_time_factor` is the time variable (modelled as a factor with 4 levels as described above), `cc_base` is cranio-caudal diameter at baseline, and `participant_id` is the participant. The model includes the fixed effects of group, time, group-by-time interaction, baseline size, and baseline size-by-time interaction. An unstructured covariance matrix is specified through: `us(MRI_time_factor|participant_id)`. The model was fitted using restricted maximum likelihood estimation of parameters (`reml = TRUE`), with the denominator degrees of freedom approximated by the Satterthwaite method (`method = "Satterthwaite"`). Treatment effect was subsequently estimated as the contrast between treatment groups at the end visit based on least-square means using the *emmeans* package. This yielded an adjusted mean difference versus placebo of –0·08 mm (95% CI –1·60 to 1·44 mm).

The Akaike information criterion (AIC) was 310 for the LME model and 274 for the MMRM, checked when fitted with maximum likelihood instead of restricted maximum likelihood. The lower AIC for the MMRM may have resulted from the simplified model with time as a 4-level categorical variable. Inspection of residuals showed some degree of leptokurtosis for both models without highly influential data points/outliers. As the LME model retained full information on the timing of assessments, results from this model were included in supplementary table 3.

Pattern-mixture model

A pattern-mixture model was applied as a sensitivity analysis for the primary outcome to explore departures from the MAR assumption and address potential bias due to differential dropout between treatment groups.[4,5] This model involved controlled multiple imputation under different post-dropout scenarios. This was implemented by shifting the MAR imputed data by a range of offsets (termed δ [delta]). First, the mean change in cranio-caudal diameter from baseline to week-72 based on the observed data of all participants who completed treatment was computed; this was 1·27 mm. Then, to explore a missing-not-at-random (MNAR) pattern in which dropouts would have experienced faster tumour growth than implied by the observed data and imputation under MAR, increasing percentages of this mean change (δ range of 25-50-75-100-200%) were added to the imputed data. Afterwards, data were re-analysed for each δ via ANCOVA as specified for the main analysis and results pooled. As there were more dropouts in the lanreotide group than in the placebo group (nine versus three), this strategy resulted in an increasingly larger mean week-72 cranio-caudal diameter in the lanreotide group versus the placebo group. Results under MAR were considered robust if an observed treatment effect was qualitatively maintained for a range of plausible offsets (supplementary table 3).

Reason for dropout was not included in the model (eg, assuming faster tumour growth only for participants who discontinued treatment due to tumour progression, while assuming a growth pattern similar to trial completers within the same treatment group for participants who discontinued treatment due to AEs), as this would be less conservative and only favour the lanreotide group.

A second, tipping-point analysis explored which δ-shift was needed to overturn the conclusion of the main analysis (ie, in which alternative post-dropout scenario would treatment effect be statistically significant).[5] This involved subtracting increasing percentages of the mean change from the MAR imputed data in the lanreotide group until the resulting treatment effect following ANCOVA was statistically significant. If the δ required to overturn the conclusion was considered clinically implausible, the results of the main analysis could be regarded with greater confidence (supplementary table 3).

References

1 Boertien TM, Drent ML, Booij J, *et al.* The GALANT trial: study protocol of a randomised placebo-controlled trial in patients with a 68Ga -DOTATATE PET-positive, clinically non-functioning pituitary macroadenoma on the effect of lanreotide on tumour size. *BMJ Open* 2020; **10**: e038250.

2 Lee DK. Data transformation: a focus on the interpretation. *Korean J Anesthesiol* 2020; **73**: 503–8.

3 Heymans MW, Eekhout I. Applied Missing Data Analysis With SPSS and R(Studio). Amsterdam: Heymans and Eekhout, 2019 https://bookdown.org/mwheymans/bookmi/.

4 Carpenter JR, Kenward MG. Missing data in randomised controlled trials–a practical guide. *Birmingham Natl Coord Cent …* 2007. http://www.hta.nhs.uk/nihrmethodology/reports/1589.pdf.

5 Cro S, Morris TP, Kenward MG, Carpenter JR. Sensitivity analysis for clinical trials with missing continuous outcome data using controlled multiple imputation: A practical guide. *Stat Med* 2020; **39**: 2815–42.

6 White IR, Royston P, Wood AM. Multiple imputation using chained equations: Issues and guidance for practice. *Stat Med* 2011; **30**: 377–99.

7 Molenberghs G, Verbeke G. Linear Mixed Models for Longitudinal Data. New York, NY: Springer New York, 2000 DOI:10.1007/978-1-4419-0300-6.

8 Dinh P, Yang P. Handling baselines in repeated measures analyses with missing data at random. *J Biopharm Stat* 2011; **21**: 326–41.

9 Molenberghs G, Verbeke G. A Model for Longitudinal Data. In: Linear Mixed Models for Longitudinal Data. New York, NY: Springer New York, 2000: 19–29.

10 Pinheiro JC, Bates DM, Team RC. nlme: Linear and Nonlinear Mixed Effects Models. 2023. https://cran.r-project.org/package=nlme.

11 Mallinckrodt CH, Lane PW, Schnell D, Peng Y, Mancuso JP. Recommendations for the Primary Analysis of Continuous Endpoints in Longitudinal Clinical Trials. *Drug Inf J* 2008; **42**: 303–19.

12 Molenberghs G, Thijs H, Jansen I, *et al.* Analyzing incomplete longitudinal clinical trial data. *Biostatistics* 2004; **5**: 445–64.

13 Sabanes Bove D, Li L, Dedic J, *et al.* mmrm: Mixed Models for Repeated Measures. 2024. https://openpharma.github.io/mmrm/.

Supplementary Tables

Table S1. Clinical characteristics and PET results of all included participants

	PET-positive (n=45)	PET-negative (n=4)
Age (years)	61·0 (8·5)	40·5 (10·0)
Female sex	16 (36%)	2 (50%)
Any pituitary hormone deficiency	26 (58%)	3 (75%)
Previous NFPMA resection	25 (56%)	1 (25%)
Maximum NFPMA diameter in any direction (mm)*	21·6 (5·5)	15·8 (3·8)
**^{68}Ga-DOTATATE PET results**		
NFPMA SUV_{mean}	5·6 (3·2–7·5)	0·7 (0·5–0·7)
NFPMA SUV_{max}	7·2 (4·5–10·7)	1·0 (0·6–1·8)

Data are mean (SD), median (IQR), or n (%). NFPMA=non-functioning pituitary macroadenoma. PET=positron emission tomography. SUV=standard uptake value. *No formal cranio-caudal measurements by two independent assessors were available for not-randomised participants.

Table S2. Primary and secondary tumour size outcomes in the per-protocol population

	Lanreotide (n=13)	Placebo (n=19)	Adjusted mean difference in change *vs* placebo (95% CI)*	p value
**Primary outcome**				
Baseline cranio-caudal diameter, mm	16·1 (3·3)	16·9 (2·8)	··	··
End cranio-caudal diameter, mm	17·4 (5·1)	18·1 (3·3)	··	··
Change in cranio-caudal diameter, mm	1·3 (3·0)	1·2 (1·6)	0·2 (–1·5 to 1·8)	0·83
**Secondary outcome**				
Baseline tumour volume. mm^3	3026 (1249)	3320 (1897)	··	··
End tumour volume, mm^3	3544 (1766)	3887 (2557)	··	··
Change in volume, mm^3	518 (686)	567 (1160)	24 (–657 to 706)	0·94

Data are mean (SD) or mean difference (95% confidence interval). The per-protocol population included participants who completed study treatment with all 18 injections and underwent week-72 MRI (deviations in visit time windows were allowed). *Adjusted for baseline tumour size using ANCOVA.

Table S3. Result overview of the main, additional and sensitivity analyses for the primary outcome

	Treatment estimate *vs* placebo	95% CI	SE	p value
**Main analysis**				
ANCOVA with while-on-treatment observed data	–0·05	–1·30 to 1·20	0·62	0·94
**Additional efficacy analyses**				
ANCOVA in per-protocol population	0·17	–1·50 to 1·84	0·81	0·83
ANCOVA after multiple imputation of missing week-72 data	0·36	–1·25 to 1·97	0·82	0·66
Linear mixed effects model	–0·13	–1·29 to 1·03	0·58	0·82
**Sensitivity analysis: pattern-mixture model***				
MAR imputed data, δ = +0·317 mm	0·46	–1·16 to 2·08	0·83	0·58
MAR imputed data, δ = +0·634 mm	0·55	–1·09 to 2·18	0·83	0·51
MAR imputed data, δ = +0·950 mm	0·64	–1·01 to 2·30	0·84	0·45
MAR imputed data, δ = +1·267 mm	0·74	–0·94 to 2·41	0·85	0·39
MAR imputed data, δ = +2·534 mm	1·11	–0·68 to 2·90	0·91	0·22
Tipping point: MAR imputed data, δ = –5·385 mm in lanreotide group	1·89	–0·002 to 3·78	0·96	0·05

Data are in millimetres. The primary outcome was the change from baseline in cranio-caudal tumour diameter. The intention-to-treat population was the basis for all analyses except the per-protocol analysis, and included all randomised participants who received at least one study injection. Treatment estimate is the baseline size-adjusted mean difference in the change from baseline. See the Statistical methods section in the appendix for details. Small differences in treatment estimate/95% CI compared to those in Table 2 and Table S2 are due to rounding to two decimal places instead of one. CI=confidence interval. MAR=missing-at-random. SE=standard error. *The increasing values of δ in the pattern-mixture model were based on 25-50-75-100-200% times the observed mean change to week-72 of 1·267 mm. For this range, results of the main and efficacy analyses were qualitatively maintained. The tipping point at which results were overturned required a δ in MAR imputed data of lanreotide-treated dropouts of –425% times the observed mean change. Such a large deviation from the MAR imputed data was considered highly implausible, supporting the results of the main and efficacy analyses. Note that larger δ-shifts lead to higher variability in the final cranio-caudal diameter values with an increase in standard errors.

Table S4. Change in quality of life based on SF-36 component scores in the intention-to-treat population

	Baseline score	Change from baseline	Adjusted mean difference in change *vs* placebo, mean (95% CI)*	p value
**Physical functioning**				
Lanreotide (n=22)	81·6 (18·9)	–0·4 (16·1)	3·8 (–6·7 to 14·2)	0·47
Placebo (n=22)	92·5 (12·4)	–5·5 (16·1)	··	··
**Role limitations due to physical health problems**				
Lanreotide (n=22)	72·7 (40·8)	–12·0 (26·9)	–9·2 (–28·2 to 9·9)	0·34
Placebo (n=22)	81·0 (35·3)	–4·8 (36·5)	··	··
**Bodily pain**				
Lanreotide (n=22)	76·6 (20·2)	–8·5 (26·1)	–6·6 (–19·0 to 5·7)	0·29
Placebo (n=22)	80·2 (23·0)	–2·9 (13·7)	··	··
**General health perceptions**				
Lanreotide (n=22)	65·1 (17·4)	–1·6 (13·7)	–1·1 (–9·3 to 7·1)	0·78
Placebo (n=22)	79·5 (18·5)	–1·1 (12·0)	··	··
**Vitality**				
Lanreotide (n=22)	62·3 (22·1)	–5·4 (17·3)	–6·5 (–15·8 to 2·9)	0·17
Placebo (n=22)	73·0 (18·0)	–0·9 (12·7)	··	··
**Social functioning**				
Lanreotide (n=22)	83 (21·0)	–5·6 (22·0)	–3·0 (–14·7 to 8·8)	0·61
Placebo (n=22)	93·8 (13·8)	–4·5 (14·2)	··	··
**Role limitations due to emotional problems**				
Lanreotide (n=22)	83·3 (36·7)	–8·7 (30·9)	–6·2 (–23·4 to 10·9)	0·47
Placebo (n=22)	97·0 (9·8)	–6·1 (24·4)	··	··
**General mental health**				
Lanreotide (n=22)	77·1 (16·1)	–4·9 (13·4)	–5·2 (–11·9 to 1·5)	0·12
Placebo (n=22)	82·9 (12·4)	0·2 (6·8)	··	··

Data are mean (SD) or mean difference (95% confidence interval). Quality of life was assessed with the 36-item Short Form Health Survey (SF-36), the eight component scores are presented. The mean imputation method was used to replace missing values for baseline 'role limitations due to physical health problems' component score of one placebo participant and all end-of-study component scores of one lanreotide participant. *Adjusted for baseline component score using ANCOVA.

Table S5. All recorded adverse events per system organ class in the safety population

	Lanreotide (n=22)	Placebo (n=22)
**Blood and lymphatic system disorders**	**0**	**2 (9%)**
Anaemia	0	1 (5%)
Polycythaemia	0	1 (5%)
**Cardiac disorders**	**6 (27%)**	**2 (9%)**
Atrial fibrillation	1 (5%)	1 (5%)
Bradycardia (<60 beats per minute)	4 (18%)	0
Chest pain or discomfort	2 (9%)	1 (5%)
**Endocrine disorders**	**13 (59%)**	**6 (27%)**
Adrenal insufficiency	1 (5%)	0
Cushing disease	1 (5%)	0
Hyperandrogenism	1 (5%)	1 (5%)
Hyperglycaemia (glucose ≥7 mmol/L)	1 (5%)	2 (9%)
Impaired fasting glucose (5·7–6·9 mmol/L)	10 (45%)	3 (14%)
**Eye disorders**	**7 (32%)**	**6 (27%)**
Blepharitis / conjunctivitis	1 (5%)	1 (5%)
Corneal erosion	0	1 (5%)
Meibomian gland dysfunction	1 (5%)	0
Ocular discomfort	1 (5%)	2 (9%)
Superior oblique myokymia	1 (5%)	0
Visual disturbances	3 (14%)	3 (14%)
**Gastrointestinal disorders**	**18 (82%)**	**8 (36%)**
Abdominal pain or discomfort	10 (45%)	2 (9%)
Crohn's disease	1 (5%)	0
Decreased appetite	2 (9%)	1 (5%)
Flatulence	4 (18%)	2 (9%)
Increased stool frequency or diarrhoea	16 (73%)	4 (18%)
Nausea or dyspepsia	8 (36%)	1 (5%)
Oral mucosa hematoma	0	1 (5%)
Papillary cystadenoma lymphomatosum	0	1 (5%)
Reflux disease	0	1 (5%)
Stomatitis	1 (5%)	0
**General disorders and administration site conditions**	**14 (64%)**	**6 (27%)**
Fatigue	5 (23%)	3 (14%)
Malaise	1 (5%)	2 (9%)
Injection site reaction	12 (55%)	0
Oedema peripheral	0	1 (5%)
Temperature intolerance	1 (5%)	0
**Hepatobiliary disorders**	**1 (5%)**	**0**
Gallbladder enlargement (incidental finding)	1 (5%)	0
**Immune system disorders**	**1 (5%)**	**1 (5%)**
Hypersensitivity reaction*	1 (5%)	1 (5%)
**Infections and infestations**	**10 (45%)**	**7 (32%)**
Influenza like illness	8 (36%)	2 (9%)
Nasopharyngitis / upper respiratory tract infection	2 (9%)	4 (18%)
Oral candidiasis	0	1 (5%)
Skin bacterial infection	0	1 (5%)
Urinary tract infection	1 (5%)	0
**Injury, poisoning and procedural complications**	**3 (14%)**	**4 (18%)**
Accident / fall	1 (5%)	2 (9%)
Contusion	1 (5%)	0
Ligament sprain / traumatic tendonitis	1 (5%)	1 (5%)
Presyncope related to blood withdrawal	0	1 (5%)
**Investigations**	**12 (55%)**	**7 (32%)**
Blood cortisol abnormal	0	1 (5%)
Blood testosterone decreased	3 (14%)	0
Blood testosterone increased	0	2 (9%)
Free thyroxine decreased	6 (27%)	1 (5%)
Free thyroxine increased	2 (9%)	0

Table S5, continued	*Lanreotide (n=22)*	*Placebo (n=22)*
Insulin-like growth factor decreased	4 (18%)	0
Hyponatraemia	0	1 (5%)
Liver function tests:		
Alanine aminotransferase increased†	1 (5%)	1 (5%)
Alkaline phosphatase increased†	1 (5%)	0
Gamma-glutamyltransferase increased‡	2 (9%)	1 (5%)
Weight decreased	2 (9%)	0
Weight increased	0	1 (5%)
**Musculoskeletal and connective tissue disorders**	**2 (9%)**	**7 (32%)**
Arthralgia	0	3 (14%)
Bursitis	0	1 (5%)
Bone marrow oedema syndrome	0	1 (5%)
Gout	1 (5%)	0
Musculoskeletal pain / back pain	1 (5%)	2 (9%)
**Neoplasms benign, malignant and unspecified**	**1 (5%)**	**2 (9%)**
Adrenal adenoma (incidental finding)	1 (5%)	0
Meningioma (incidental finding)	0	1 (5%)
Schwannoma (incidental finding)	0	1 (5%)
**Nervous system disorders**	**8 (36%)**	**8 (36%)**
Dizziness / light-headedness / vertigo	2 (9%)	3 (14%)
Epilepsy aggravated	0	1 (5%)
Headache	7 (32%)	4 (18%)
Neuropathy / paraesthesia	2 (9%)	1 (5%)
**Psychiatric disorders**	**1 (5%)**	**1 (5%)**
Acute stress reaction	1 (5%)	0
Claustrophobia	0	1 (5%)
**Renal and urinary disorders**	**1 (5%)**	**1 (5%)**
Haematuria	0	1 (5%)
Renal impairment	1 (5%)	0
**Reproductive system and breast disorders**	**2 (9%)**	**0**
Uterine prolapse / cystocele	2 (9%)	0
**Respiratory, thoracic and mediastinal disorders**	**2 (9%)**	**3 (14%)**
Dyspnoea	1 (5%)	2 (9%)
Hyposmia / parosmia	1 (5%)	1 (5%)
**Skin and subcutaneous tissue disorders**	**5 (23%)**	**7 (32%)**
Alopecia	5 (23%)	0
Benign neoplasm of skin	0	2 (9%)
Eczema / dermatitis	1 (5%)	2 (9%)
Folliculitis	0	1 (5%)
Intertrigo	0	2 (9%)
**Surgical and medical procedures**	**3 (14%)**	**4 (18%)**
Eyelid operation	0	1 (5%)
Pituitary tumour removal	2 (9%)	2 (9%)
Varicose vein operation	0	1 (5%)
Wisdom tooth removal	1 (5%)	0
**Vascular disorders**	**3 (14%)**	**5 (23%)**
Cerebrovascular arteriovenous malformation (incidental finding)	1 (5%)	0
Hot flushes	0	2 (9%)
Hypertension	2 (9%)	3 (14%)

Data are n (%). The safety population included all randomised participants who received at least one study injection. An adverse event is defined as any undesirable finding or experience occurring to a participant during the study (between signing of informed consent and up to 30 days after study completion or treatment discontinuation), whether or not considered related to the study or study treatment. Adverse events were coded and grouped using the Medical Dictionary for Regulatory Activities (MedDRA version 24·0). One participant may experience multiple adverse events within a specific system organ class, either during the same event (eg, one event comprising abdominal pain and nausea), or as two separate events (eg, fatigue and injection site reaction). *Hypersensitivity reaction to other substance than study treatment/injection. †At least two times the upper limit of normal. ‡At least 20 U/L above the upper limit of normal.

Supplementary Figures

Figure S1. Individual change in tumour size from baseline to end-of-treatment MRI

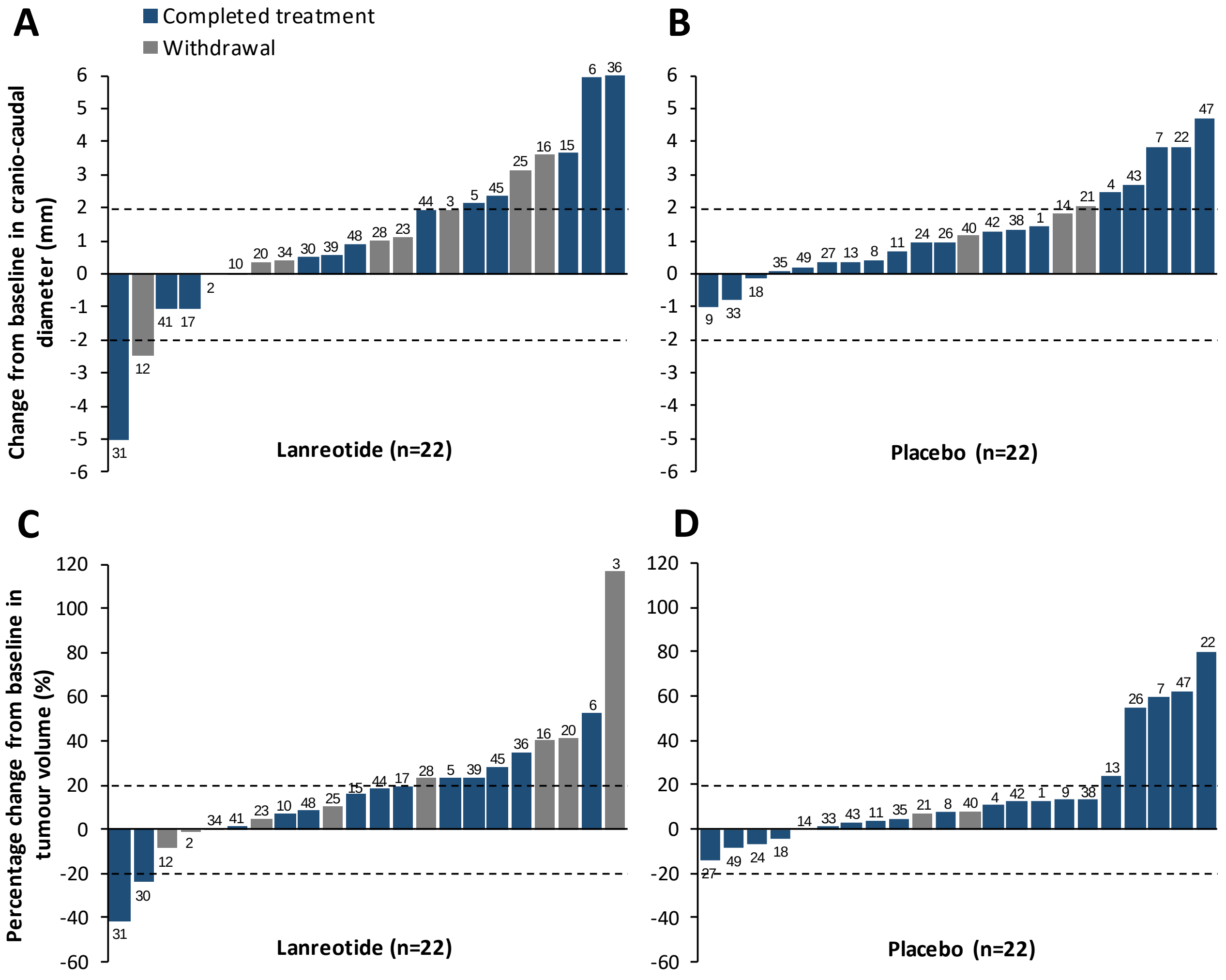

Figure S1. Upper panels: change in cranio-caudal diameter (in millimetres) from baseline to end-of-treatment measurement for individual participants in (**A**) the lanreotide group and (**B**) the placebo group. Lower panels: percentage change in tumour volume from baseline to end-of-treatment measurement for individual participants in (**C**) the lanreotide group and (**D**) the placebo group. The participant's study number at each bar enables quick cross-referencing between cranio-caudal diameter and tumour volume charts. The dashed horizontal lines denote clinically significant change in tumour size.

Figure S2. Time to progression based on tumour volume or cranio-caudal size

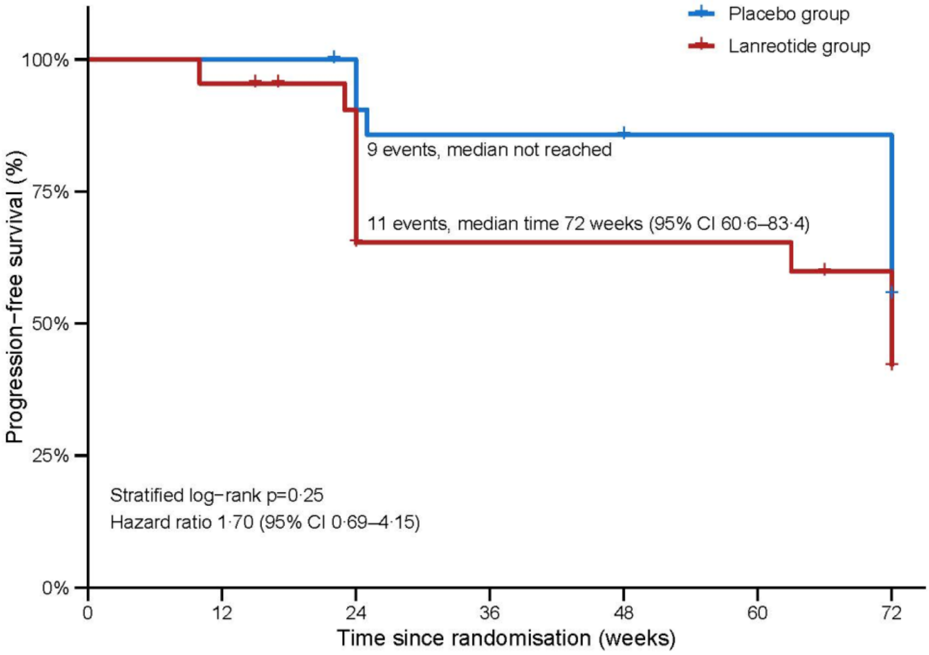

Number at risk (number censored)

	0	12	24	36	48	60	72
Placebo group	22 (0)	22 (0)	21 (1)	18 (1)	18 (2)	17 (2)	17 (13)
Lanreotide group	22 (0)	21 (0)	18 (3)	12 (3)	12 (3)	12 (3)	10 (11)

Figure S2. Kaplan-Meier estimates of progression-free survival for the post-hoc outcome time to tumour progression based on significant increase in either tumour volume (≥20%) or cranio-caudal diameter (≥2 mm). Tick marks indicate censored data. Outcome data at time of censoring was available for all participants. Time to progression was compared between groups using the stratified log-rank test, with stratification for presence or absence of documented tumour growth at baseline. The hazard ratio was derived from a Cox proportional-hazards model with terms for study treatment and tumour growth at baseline; there was no statistically significant interaction between these terms. The difference between 9 events of tumour progression in the placebo group shown here and 8 participants with significant increase in tumour size described in the main text is due to 1 participant who showed significant increase in cranio-caudal diameter of 2·1 mm at week-24 MRI, but had a subsequent decrease in tumour size resulting in a final change in diameter of +1·0 mm at week-72 MRI.

Figure S3. SF-36 component score spider charts

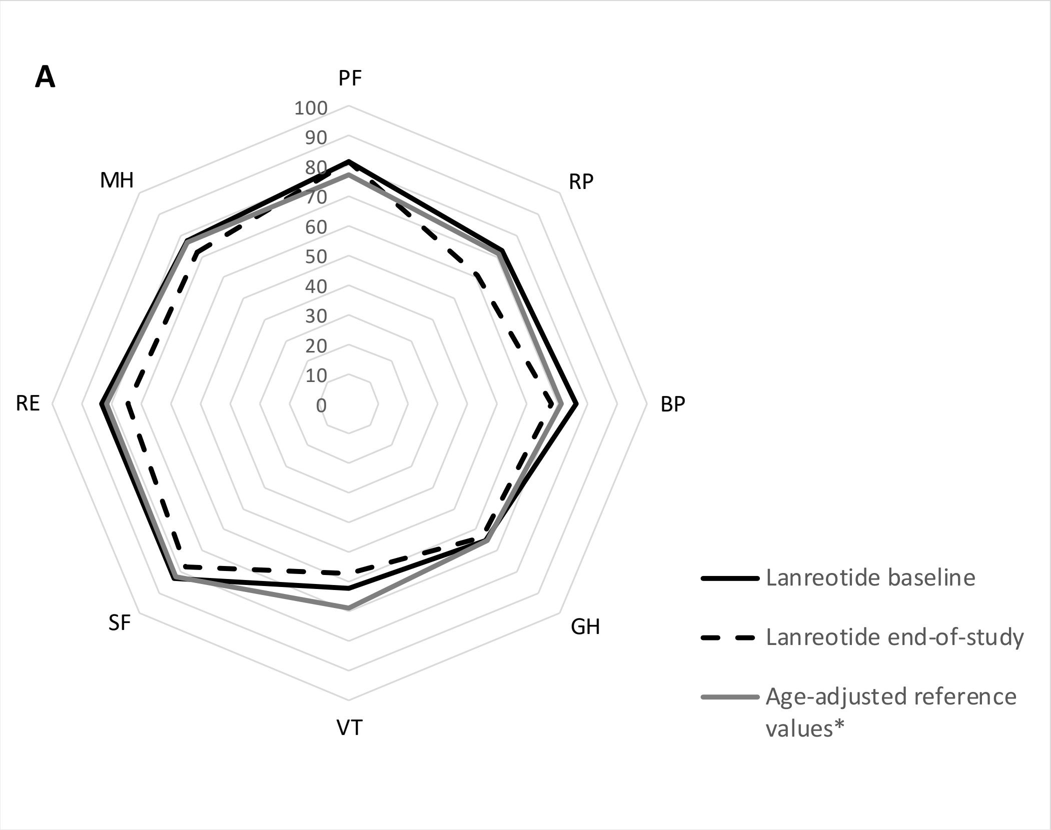

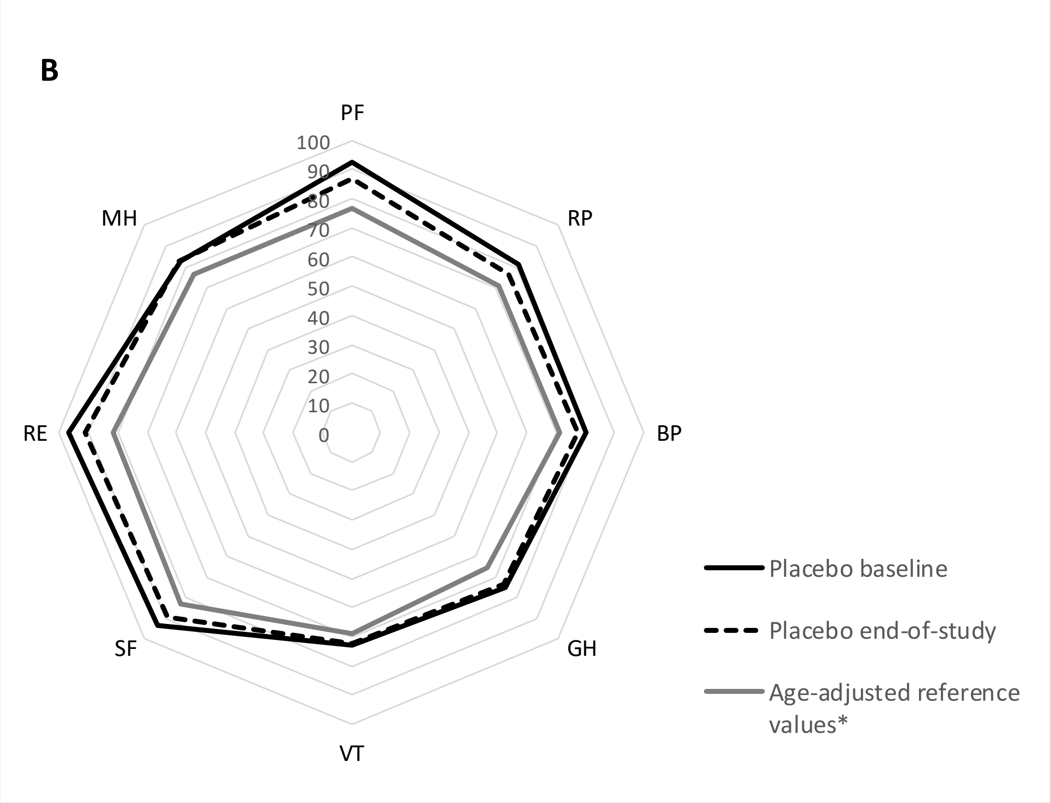

Figure S3. Spider plots of mean SF-36 component scores at baseline and end-of-study for (**A**) lanreotide group, and (**B**) placebo group. *Age-adjusted reference values were derived from Aaronson et al. [21]. PF=physical functioning, RP=role limitations due to physical health problems, BP=bodily pain, GH=general health perceptions, VT=vitality, SF=social functioning, RE=role limitations due to emotional problems, MH=general mental health.
