# Supplementary material for: Lanreotide versus placebo for tumour reduction in patients with a 68Ga-DOTATATE PET-positive, clinically non-functioning pituitary macroadenoma (GALANT study): a randomised, multicentre, phase 3 trial with blinded outcome assessment
Source: Lancet Reg Health Eur. 2024 May 13;42:100923. doi: 10.1016/j.lanepe.2024.100923 (PMC11281922; doi:10.1016/j.lanepe.2024.100923)
Supplement: Study protocol GALANT trial [file mmc2.pdf]

A randomized placebo-controlled study in patients with a Gallium-68 DOTATATE PET/CT positive, clinically non-functioning pituitary macroadenoma (NFMA) of the effect of Lanreotide autosolution on Tumor (adenoma) size (GALANT)

(version 5.0, 30 November 2018)

PROTOCOL TITLE

A randomized placebo-controlled study in patients with a Gallium-68 DOTATATE PET/CT positive, clinically non-functioning pituitary macroadenoma (NFMA) of the effect of Lanreotide autosolution* on Tumor (adenoma) size (GALANT)

Dutch: Een gerandomiseerde, placebo-gecontroleerde studie onder patiënten met Gallium-68 DOTATATE PET/CT positieve, klinisch niet-functionerende hypofyse macroadenomen (NFMA) naar het effect van Lanreotide autosolution* op Tumor(adenoom)grootte (GALANT)

*Official marketing name: Somatuline® AutoSolution®, Ipsen Farmaceutica

**Protocol ID**	NL 52821.018.15 / GALANT
**Short title**<br><br>**Dutch:**	Efficacy of lanreotide in patients with Gallium-68 DOTATATE PET/CT positive NFMA<br><br>Effectiviteit van lanreotide bij patiënten met NFMA en een positieve Gallium-68 DOTATATE PET/CT
**Title for lay people**<br><br>**Dutch:**	The effect of lanreotide on non-functioning pituitary macroadenoma size<br><br>Het effect van lanreotide op de grootte van niet-functionerende hypofyse macroadenomen
**EudraCT number**	2015-001234-22
**Version**	5.0
**Date**	30 November 2018
**Coordinating investigator/project leader**	T.M. Boertien, MD<br>Academic Medical Center, University of Amsterdam<br>Dept. of Endocrinology & Metabolism,<br>room F5-176<br>Meibergdreef 9, 1105 AZ Amsterdam<br>Tel: +31 20 566 60 71<br>t.m.boertien@amc.uva.nl
**Principal investigators (in Dutch: hoofdonderzoekers/uitvoerders)**	**AMC**<br>Prof. dr. E. Fliers - Principal Investigator<br>Academic Medical Center, University of Amsterdam<br>Dept. of Endocrinology & Metabolism, room F5-171<br>Meibergdreef 9, 1105 AZ Amsterdam<br>e.fliers@amc.uva.nl<br><br>**VUmc**<br>Prof. dr. M.L. Drent - local Principal Investigator<br>VU Medical Center, VU University Amsterdam<br>Dept. of Internal Medicine, section of Endocrinology,<br>room 4A 61<br>De Boelelaan 1117, 1081 HV Amsterdam

	ml.drent@vumc.nl<br><br>**NKI**<br>Dr. M.P.M. Stokkel - local Principal Investigator<br>Nederlands Kanker Instituut<br>Dept. of Nuclear Medicine, room C0-135<br>Plesmanlaan 121, 1066 CS Amsterdam<br>m.stokkel@nki.nl<br><br>**LUMC**<br>Prof dr. A.M. Pereira Arias- local Principal Investigator<br>Leiden University Medical Center<br>Dept. of Internal Medicine, section of Endocrinology, room C-07-050<br>Albinusdreef 2, 2333 ZA Leiden<br>a.m.pereira@lumc.nl
**Sponsor (in Dutch: verrichter/opdrachtgever)**	Prof. dr. E. Fliers<br>Academic Medical Center, University of Amsterdam<br>Dept. of Endocrinology & Metabolism, room F5-171<br>Meibergdreef 9, 1105 AZ Amsterdam<br>e.fliers@amc.uva.nl
**Subsidising party**	Ipsen Farmaceutica B.V.<br>Taurusavenue 33b<br>2132 LS Hoofddorp
**Independent expert (s)**	**AMC**<br>Dr. M.R. Soeters<br>Academic Medical Center, University of Amsterdam<br>Dept. of Endocrinology & Metabolism, room F5-161<br>Meibergdreef 9, 1105 AZ Amsterdam<br>m.r.soeters@amc.uva.nl<br><br>**VUmc**<br>Dr. E.M.W. Eekhoff<br>VU Medical Center, VU University Amsterdam<br>Dept. of Internal Medicine, section of Endocrinology, room 4A 60<br>De Boelelaan 1117, 1081 HV Amsterdam<br>emw.eekhoff@vumc.nl<br><br>**LUMC**<br>Prof. dr. H. Pijl<br>Dept. of Internal Medicine, section of Endocrinology, room C7-056<br>Albinusdreef 2, 2333 ZA Leiden<br>h.pijl@lumc.nl
**Laboratory sites**	**AMC**<br>Laboratory of Endocrinology and Radiochemistry<br>Academic Medical Center, University of Amsterdam

	Meibergdreef 9, 1105 AZ Amsterdam<br><br>**VUmc**<br>Laboratory of Endocrinology<br>VU Medical Center, VU University Amsterdam<br>De Boelelaan 1117, 1081 HV Amsterdam<br><br>**LUMC**<br>Laboratory of Clinical Chemistry<br>Leiden University Medical Center<br>Albinusdreef 2, 2333 ZA Leiden
**Pharmacy**	**AMC – IMPs**<br>AMC Trial Pharmacy, E0-224<br>(Knowledge Centre Drug Research)<br>Postbus 22660, 1100 DD Amsterdam<br>Tel: +31 (0) 20 5663871<br>trial.apotheek@amc.uva.nl<br><br>**AMC – NIMP**<br>Y. Chahid, clinical pharmacist<br>AMC Hospital Pharmacy<br>Dept. RASA (Radio Actieve Stoffen Apotheek)<br>Postbus 22660, 1100 DD Amsterdam<br>Tel: +31 (0) 20 5667280<br>y.chahid@amc.uva.nl<br><br>**NKI-AvL – NIMP**<br>Dr. A.D.R. Huitema, clinical pharmacist<br>NKI-AvL, Pharmacology<br>Plesmanlaan 121, 1066 CX Amsterdam<br>Tel: +31 (0) 20 5124481<br>a.huitema@nki.nl

PROTOCOL SIGNATURE SHEET

Name	Signature	Date
**Sponsor or legal representative:**<br>Prof. dr. E. Fliers<br>Internist-endocrinologist<br><br>**Head of Department:**<br>Prof. dr. J.M. Prins<br>Head of Department of Internal Medicine		13 DEC 2018<br><br><br><br>14 DEC 2018
**Coordinating Investigator / Project leader**<br>Drs. T.M. Boertien, PhD candidate		13-dec-2018

TABLE OF CONTENTS

1. INTRODUCTION AND RATIONALE 11
2. OBJECTIVES 13
3. STUDY DESIGN 13
4. STUDY POPULATION 14
4.1 Population (base) 14
4.2 Inclusion criteria 14
4.3 Exclusion criteria 14
4.4 Sample size calculation 15
5. TREATMENT OF SUBJECTS 16
5.1 Investigational product/treatment 16
5.2 Use of co-intervention 16
5.3 Escape medication (if applicable) 16
6. INVESTIGATIONAL PRODUCT 17
6.1 Name and description of investigational product(s) 17
6.2 Summary of findings from non-clinical studies 17
6.3 Summary of findings from clinical studies 17
6.4 Summary of known and potential risks and benefits 17
6.5 Description and justification of route of administration and dosage 17
6.6 Dosages, dosage modifications and method of administration 17
6.7 Preparation and labelling of Investigational Medicinal Product 17
6.8 Drug accountability 18
7. NON-INVESTIGATIONAL PRODUCT (NIMP) 19
7.1 Name and description of non-investigational product(s) 19
7.2 Summary of findings from non-clinical studies 19
7.3 Summary of findings from clinical studies 19
7.4 Summary of known and potential risks and benefits 19
7.5 Description and justification of route of administration and dosage 19
7.6 Dosages, dosage modifications and method of administration 19
7.7 Preparation and labelling of Non Investigational Medicinal Products 19
7.8 Drug accountability 19
8 METHODS 20
8.1 Study parameters/endpoints 20
8.1.1 Main study parameter/endpoint 20
8.1.2 Secondary study parameters/endpoints 20
8.1.3 Other study parameters 21
8.2 Randomization, blinding and treatment allocation 21
8.3 Study procedures 22
8.4 Withdrawal of individual subjects 24
8.4.1 Specific criteria for withdrawal 24
8.5 Replacement of individual subjects after withdrawal 24
8.6 Follow-up of subjects withdrawn from treatment 24

8.7 Premature termination of the study 24
9 SAFETY REPORTING 25
9.1 Temporary halt for reasons of subject safety 25
9.2 AEs, SAEs and SUSARs 25
9.2.1 Adverse events (AEs) 25
9.2.2 Serious adverse events (SAEs) 25
9.2.3 Suspected unexpected serious adverse reactions (SUSARs) 26
9.3 Annual safety report 27
9.4 Follow-up of adverse events 27
9.5 Data Safety Monitoring Board (DSMB) / Safety Committee 27
10. STATISTICAL ANALYSIS 28
10.1 Primary study parameter 28
10.2 Secondary study parameters 28
10.3 Other study parameters 29
10.4 Interim analysis (if applicable) 29
11. ETHICAL CONSIDERATIONS 30
11.1 Regulation statement 30
11.2 Recruitment and consent 30
11.3 Objection by minors or incapacitated subjects (if applicable) 30
11.4 Benefits and risks assessment, group relatedness 30
11.5 Compensation for injury 31
11.6 Incentives (if applicable) 31
12 ADMINISTRATIVE ASPECTS, MONITORING AND PUBLICATION 32
12.1 Handling and storage of data and documents 32
12.2 Monitoring and Quality Assurance 32
12.3 Amendments 32
12.4 Annual progress report 32
12.5 Temporary halt and (prematurely) end of study report 32
12.6 Public disclosure and publication policy 33
13 STRUCTURED RISK ANALYSIS 34
13.1 Potential issues of concern 34
13.2 Synthesis 35
14 REFERENCES 37

LIST OF ABBREVIATIONS AND RELEVANT DEFINITIONS

**ABR**	**ABR form, General Assessment and Registration form, is the application form that is required for submission to the accredited Ethics Committee (In Dutch, ABR = Algemene Beoordeling en Registratie)**
**AE**	**Adverse Event**
**AMC**	**Academical Medical Center**
**AR**	**Adverse Reaction**
**CA**	**Competent Authority**
**CCMO**	**Central Committee on Research Involving Human Subjects; in Dutch: Centrale Commissie Mensgebonden Onderzoek**
**CV**	**Curriculum Vitae**
**DSMB**	**Data Safety Monitoring Board**
**EU**	**European Union**
**EudraCT**	**European drug regulatory affairs Clinical Trials**
**GCP**	**Good Clinical Practice**
**IB**	**Investigator's Brochure**
**IC**	**Informed Consent**
**IMP**	**Investigational Medicinal Product**
**IMPD**	**Investigational Medicinal Product Dossier**
**ITT**	**Intention-to-treat**
**LUMC**	**Leiden University Medical Center**
**MBq**	**megabecquerel**
**METC**	**Medical research ethics committee (MREC); in Dutch: medisch ethische toetsing commissie (METC)**
**MRI**	**Magnetic resonance imaging**
**mSv**	**millisievert**
**NFMA**	**Non-functioning macroadenoma**
**NKI**	**Nederlands Kanker Instituut**
**PET/CT**	**Positron emission tomography–computed tomography**
**RCT**	**Randomized controlled trial**
**(S)AE**	**(Serious) Adverse Event**
**SPC**	**Summary of Product Characteristics (in Dutch: officiële productinfomatie IB1-tekst)**
**SPECT**	**Single-photon emission computed tomography**
**Sponsor**	**The sponsor is the party that commissions the organisation or performance of the research, for example a pharmaceutical company, academic hospital, scientific organisation or investigator. A party that provides funding for a study but does not commission it is not regarded as the sponsor, but referred to as a subsidising party.**
**SSTR**	**Somatostatin receptor**
**SUSAR**	**Suspected Unexpected Serious Adverse Reaction**
**VUmc**	**VU (Vrije Universiteit) Medical Center**
**SUV**	**Standardized uptake value**
**Wbp**	**Personal Data Protection Act (in Dutch: Wet Bescherming Persoonsgevens)**
**WMO**	**Medical Research Involving Human Subjects Act (in Dutch: Wet Medisch-wetenschappelijk Onderzoek met Mensen)**

SUMMARY

Rationale: In patients with clinically non-functioning pituitary macroadenomas (NFMA), the current therapeutic approach is to substitute any hormonal deficits, and to perform transsphenoidal surgery if necessary, based on optic chiasm compression or visual field defects. In the literature, remission rate after surgery is estimated to be about 44%, which is substantially lower than in other pituitary tumor types, and recurrence rate is about 11% during an average follow-up period of 5 years [1]. In patients with NFMA without giving rise to visual field defects, a wait-and-see approach is commonly followed. Whereas medical therapy is regarded as first-line treatment in prolactinoma patients, the role for medical therapy in patients with NFMA is controversial. Although in surgical specimens the majority of NFMA express somatostatin receptors [2], in vivo assessment of NFMA by Indium-111 pentetreotide SPECT yielded lower, and sometimes conflicting positive rates. This may be caused by differences in subjective evaluation of these scans. A well-defined ROI method may be helpful in this respect, but the spatial resolution of SPECT is insufficient to differentiate NFMA from normal anterior pituitary tissue. Octreotide, a somatostatin analogue, has been shown to be able to reduce tumor size in ~12% of patients with NFMA [3]. However, the number of patients studied was small, follow-up was short, and no randomized controlled trials (RCTs) have been published.

Thus, somatostatin analogues may have potential in some NFMA patients, but there is a remarkable lack of randomized and controlled studies. Furthermore, it is not possible at present to predict which patients will respond to somatostatin analogues. A recent pilot study in the NKI using a new method of somatostatin receptor imaging with PET showed marked uptake of Gallium-68 DOTATATE in a small number of patients with acromegaly. The sensitivity and spatial resolution of clinical PET/CT is superior to SPECT/CT, and allows for accurate quantification of radioligand uptake within neuroendocrine tumors [4]. **We hypothesize that positive somatostatin receptor imaging in pituitary NFMA using Gallium-68 DOTATATE PET/CT predicts response to somatostatin analogue therapy**. At present, the positive rate of pituitary somatostatin receptor imaging using Gallium-68 DOTATATE PET/CT in patients with clinically non-functioning pituitary macroadenomas (NFMA) is unknown. In the present study, we will randomize patients with NFMA and positive pituitary somatostatin receptor imaging using Gallium-68 DOTATATE PET/CT to receive lanreotide autosolution therapy or placebo treatment during 18 months to investigate if lanreotide reduces NFMA diameter in these patients.

Objective: To investigate the efficacy of lanreotide autosolution during 18 months, as compared to placebo, to reduce and/or stabilize adenoma size in patients with NFMA and positive pituitary somatostatin receptor imaging using Gallium-68 DOTATATE PET/CT.

Study design: Double-blind, randomized, placebo-controlled trial

Study population: Adult patients with NFMA with suprasellar extension who are treated at the outpatient clinics of the AMC, VUmc and LUMC. Only patients with a positive Gallium-68 DOTATATE PET/CT scan will be randomized for treatment.

Intervention: One group receives treatment with lanreotide autosolution 120 mg injections every 4 weeks during 18 months, and the other group will receive treatment with placebo injections, consisting of saline, during 18 months

Main study parameters/endpoints: Primary efficacy variable: Pituitary MRI at baseline and after 18 months of lanreotide autosolution or placebo. The cranio-caudal size will be determined by 2 radiologists, who are blinded to treatment group and timing. Cranio-caudal size will be expressed in millimetres. The cranio-caudal size used for the primary endpoint will be the mean of the results of 2 radiologists. In case of a difference >2 mm between the 2 radiologists, the images will be offered for second review. In case of a persistent difference >2 mm, a 3rd radiologist will review the images and the average of 3 radiologists will be used for the primary endpoint.

Nature and extent of the burden and risks associated with participation, benefit and group relatedness: Interested patients with NFMA will be invited for visit 1 at their own center (informed consent, in- and exclusion criteria, quality of life questionnaire, physical exam, venipuncture, pituitary MRI, and – if applicable – a pregnancy test). The physical exam, venipuncture and pituitary MRI are part of standard NFMA evaluation. Included patients will undergo a Gallium-68 DOTATATE PET/CT scan at the AMC. The scan is very well tolerated and the total radiation exposure is estimated at 3.1 mSv (millisievert). The first 44 patients with a positive PET/CT will be randomized to receiving a deep subcutaneously injection of lanreotide autosolution 120 mg, or to receiving placebo injection consisting of saline once every 4 weeks for 18 months (18 visits). The injections will either be administered at the clinical Endocine Unit of the AMC by an endocrine nurse, or at home by trained nurses from Eurocept Homecare. Treatment with lanreotide autosolution 120 mg was shown to be safe and well-tolerated as a first-line therapy in patients with growth hormone secreting pituitary adenomas [5]. It does carry a small risk of developing symptomatic gall stones. In ~10% of patients it may cause diarrhea, loose stools, or abdominal pain, especially after the first injection. In <10% it may cause injection site reactions. Pharmacological studies in animals and humans show that lanreotide, like somatostatin and other somatostatin analogues, inhibits the secretion of insulin and glucagon. Hence, patients treated with lanreotide may experience mild hypoglycemia or hyperglycemia. Blood glucose and HbA1c levels will be monitored during the study and any anti-diabetic treatment will be adjusted accordingly. Every 24 weeks or so a study visit takes place, consisting of a short physical examination (± 5 minutes), blood tests (8 vials via one venipuncture, total volume 36 milliliters), a quality of life questionnaire and documentation of adverse events. Pituitary MRI will be repeated after 6 months and after 18 months. Again, the physical exam, the blood tests and the MRI are part of standard NFMA care. The total number of study visits to the hospital depends on where the injections are administered. The minimum is 4 visits for AMC patients and 5 visits for VUmc and LUMC patients, the maximum is 21 visits. During the study period, patients are not deprived of any standard therapy. The benefit is potentially reduced NFMA growth rate, which may postpone or obviate transsphenoidal surgery or radiotherapy. If this study shows a positive effect on tumor size, standard treatment of NFMA could be improved in a major way.

1. INTRODUCTION AND RATIONALE

In patients with clinically non-functioning pituitary macroadenomas (NFMA), the current therapeutic approach is to substitute any hormonal deficits, and to perform transsphenoidal surgery if necessary, based on optic chiasm compression or visual field defects. In Amsterdam alone, approximately 40 of these patients per year undergo surgery. In the literature, remission rate after surgery is estimated to be about 44%, which is substantially lower than in other pituitary tumor types, and recurrence rate is about 11% during an average follow-up period of 5 years [1]. In patients with substantial remnants after surgery, radiotherapy may help to control tumor growth, but this is associated with long-term side effects such as hypopituitarism, optic nerve atrophy and increased cerebrovascular mortality [6]. In patients with small NFA, and in patients with larger tumors without giving rise to visual field defects, a wait-and-see approach is commonly followed. This policy is based on the rather slow growth rate of NFA, which was only 0.6 mm/year in one series of non-operated patients [7]. In these patients, MRI is advised with intervals of 1-3 years and evaluation of visual fields when appropriate [6]. However, the available evidence concerning treatment and follow-up of NFA is based exclusively on small, observational studies, and there is a remarkable lack of randomized studies [3, 6].

Whereas medical therapy is regarded as first-line treatment in prolactinoma patients, and is currently considered an excellent alternative for surgery in acromegaly patients, the role for medical therapy in patients with clinically non-functioning pituitary adenomas is controversial. The efficacy of therapy with dopaminergic drugs, including the dopamine receptor agonists bromocriptine and cabergoline, has generally been disappointing, although it should be noted that only small observational series have been published at the time of preparing this protocol. Although NFMA have high affinity dopaminergic receptor binding sites using [^{3}H]spiperone as a radioligand, the number of binding sites was only 18.8% of that seen in prolactinomas and similar to normal pituitaries (for review see [8]), which may explain the rather disappointing therapeutic results. In 2016, Greenman *et al.* published a historical cohort analysis of the effect of dopamine agonist therapy in a large series of postoperative NFMA patients [9]. In 55 patients in which dopamine agonist therapy was initiated in case of tumor remnant on the first postoperative MRI, tumor shrinkage was seen in 38% of patients after a mean follow-up time of 6.7 years. Tumor progression was seen in only 13% of patients. In 60 control patients with postoperative tumor remnants who received no medical treatment, tumor shrinkage was absent whereas tumor growth was seen in 47% of patients. Based on these results, a role for postoperative dopamine agonist therapy in NFMA is conceivable, although it is not clear which patients might benefit most and results of a proper placebo-controlled RCT are lacking.

It has been known for some decades that NFMA may express somatostatin receptors. In surgical specimens, the majority of NFMA may express somatostatin receptors [2], but in vivo assessment yielded lower, and sometimes conflicting positive rates. However, published series were mostly on very small patient groups. Plöckinger et al. studied 12 patients with NFMA, and reported a positive Indium-111 pentetreotide SPECT scan in n=4 (33%), without a clear correlation with the response to 3 months octreotide treatment [10]. Two further studies by Broson-Chazot et al. and Oppizzi et al. found

increased uptake in a higher amount of patients, namely 18/29 (62%) and 14/22 (64%), respectively [11, 12]. However, treatment with octreotide did not lead to improvement of visual disturbances [11] or tumor shrinkage [12]. It is important to note that the normal anterior pituitary gland takes up ^{111}In-pentetreotide as well. Thus, conflicting results in NFMA may be caused by differences in subjective evaluation of these scans. A well-defined ROI method may be helpful in this respect, but the spatial resolution of SPECT is insufficient to differentiate NFMA from normal anterior pituitary tissue. In sum, the role of somatostatin receptor scintigraphy using SPECT in patients with pituitary tumors, including NFMA, is very limited [13]. Octreotide, a somatostatin analogue, has been shown to reduce tumor size in some patients with NFMA with concomitant improvement in visual field defects. Initial studies focused on very small patient series (e.g., only 4 patients on octreotide treatment by De Bruin et al., 1992). More recently, Colao et al. (2008) reviewed the available literature and concluded that tumor reduction by somatostatin analogues can be seen in 12% of NFMA cases, whereas the majority had stable tumors [3]. However, the number of patients studied was small, follow-up was short (6 months on average) and no randomized controlled trials (RCTs) have been published.

Thus, somatostatin analogues may have potential in some NFMA patients, but there is a remarkable lack of randomized and controlled studies. Furthermore, it is not possible at present to predict which patients will respond to somatostatin analogues. A recent pilot study in the NKI using a new method of somatostatin receptor imaging with PET showed marked uptake of Gallium-68 DOTATATE in a small number of patients with acromegaly. The sensitivity and spatial resolution of clinical PET/CT is superior to SPECT/CT, and allows for accurate quantification of radioligand uptake within neuroendocrine tumors [4].

We hypothesize that positive somatostatin receptor imaging in pituitary NFMA using Gallium-68 DOTATATE PET/CT predicts response to somatostatin analogue therapy. At present, the positive rate of pituitary somatostatin receptor imaging using Gallium-68 DOTATATE PET/CT in patients with clinically non-functioning pituitary macroadenomas (NFMA) is unknown. In the present study, we will randomize patients with NFMA and positive pituitary somatostatin receptor imaging using Gallium-68 DOTATATE PET/CT to receive lanreotide autosolution therapy or placebo treatment during 18 months to investigate if lanreotide reduces NFMA diameter in these patients.

2. OBJECTIVES

Primary Objective: to compare the change in cranio-caudal NFMA size over 18 months between the lanreotide autosolution 120 mg and placebo group.
Primary efficacy variable: Change in cranio-caudal NFMA size (mm) over 18 months.

Secondary Objectives:
1. To assess the safety of lanreotide use in NFMA based on the number of (serious) adverse events.
2. To compare the change in quality of life using a standardized questionnaire (SF-36) between the lanreotide autosolution 120 mg and placebo group.
3. To compare the change in NFMA volume measured on pituitary 3DT1 MRI over 18 months between the lanreotide autosolution 120 mg and placebo group.
4. To compare time to tumor progression (i.e. tumor growth) between the lanreotide autosolution 120 mg and placebo group.

3. STUDY DESIGN

Double-blind, randomized, placebo-controlled trial.
Setting: outpatient setting.
Duration: 18 months.
After obtaining informed consent all subjects will undergo standard endocrine evaluation, including pituitary MRI if necessary, and a Gallium-68 DOTATATE PET/CT (week -2 to week 0). The PET/CT images will be co-registered with the MR images. Then, the macroadenoma will be delineated on the MR image, and the SUVmean will be calculated. A SUVmean of the tumor > 2 will be considered a positive Gallium-68 DOTATATE PET/CT scan. The first 44 patients with a positive scan result will be randomized for inclusion in either the intervention arm (lanreotide autosolution 120 mg injection every 4 weeks) or the control arm (placebo: saline injections). See chapter 8.3 for a detailed schedule of procedures (Table 1.).

Figure 1 (v5.0). Study flow chart

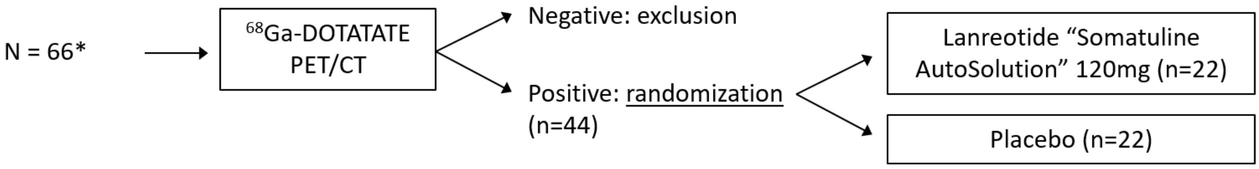

*estimation based on a minimum expected PET-positivity in ⅔ of patients

4. STUDY POPULATION

4.1 Population (base)

The research population consists of all adult (18 years or older) patients who are treated in the AMC, the VUmc and the LUMC for non-functioning pituitary adenoma (NFA). At the Outpatient Departments of Endocrinology of the AMC and VUmc together, 264 patients are currently being treated for NFA according to a recent DBC screen. However, a substantial part of these patients were found to be ineligible when considering the criteria. The LUMC has therefore been added as participating center to expand the research population. Patients with NFMA who have previously undergone transsphenoidal surgery can be included if there is evidence on recent pituitary MRI of residual adenoma or recurrence of more than 10 mm.

We will perform the RCT in 44 NFMA patients with a positive Gallium-68 DOTATATE PET/CT scan and a tumor with suprasellar extension without giving rise to visual field defects due to optic chiasm compression. Based on previous reports that about ⅓ to ⅔ of NFMA patients have positive imaging using Indium-111 pentetreotide SPECT [10–12], and our own findings thus far of >⅔ PET-positivity using the superior Gallium-68 DOTATATE PET/CT [14] , we expect to need to enroll a maximum of 66 patients to be able to randomize 44 patients. It is likely that we will be able to enroll all patients from the combined research population of the AMC, VUmc and LUMC.

4.2 Inclusion criteria

In order to be eligible to participate in this study, a subject must meet all of the following criteria:

- NFMA with suprasellar extension (in previously operated patients: residual adenoma or recurrence > 10 mm)
- Positive Gallium-68 DOTATATE PET/CT scan (SUVmean of the tumor > 2; see chapter 3)

4.3 Exclusion criteria

A potential subject who meets any of the following criteria will be excluded from participation in this study:

- Hypersensitivity to somatostatin or similar peptides
- Optic chiasm compression with visual field defects
- Obstructive neuroendocrine gut tumor
- Previous radiation therapy in the pituitary region
- Symptomatic cholelithiasis (previously proven using abdominal ultrasound or another adequate imaging modality)
- Use of dopamine agonists in the past 6 months
- Use of somatostatin analogues in the past 6 months
- Pregnancy (or wish to conceive)

- Any contraindication to perform MRI with gadolinium-based contrast agent (including implanted metallic devices, impaired renal function and severe claustrophobia)

4.4 Sample size calculation

We will perform the RCT in 44 NFMA patients with a positive Gallium-68 DOTATATE PET/CT scan. This study is powered to detect a mean difference in cranio-caudal size of 2 mm (standard deviation 1.9 mm) between the groups after 18 months with a power of 80% and a significance level of 0.05 using a 2-sided t-test (nQuery Advisor 7.0). The amended sample size takes into account an observed dropout rate of ~27%. Dropout is defined as failure to complete all 18 trial injections and undergo final MRI due to any reason. Data of all subjects randomized for treatment will be analyzed following an intention-to-treat (ITT) design. The value of 2 mm has been selected on the basis of its clinical relevance. Such a difference in cranio-caudal size of the tumor is sufficient to prevent complications related to tumor growth and therefore transsphenoidal surgery. The final analysis of the primary endpoint will be performed by ANCOVA (0.05 level of significance). Although ANCOVA may reduce the number of patients required for an RCT, we prefer a t-test for the power calculation. A t-test based power analysis is more conservative and therefore less likely to result in an under powered study, because a reliable estimation of Rho is not possible for this study (cf Borm et al. [15]).

5. TREATMENT OF SUBJECTS

5.1 Investigational product/treatment

Participants will be treated with either lanreotide autosolution 120 mg subcutaneously every 4 weeks or with placebo injections (consisting of saline 0.9%) every 4 weeks during 18 months. Lanreotide and placebo will be provided to the participants free of charge.
Lanreotide autosolution 120 mg is the highest dose currently available. This dose is used routinely for various therapeutic indications (see the attached SPC of Somatuline® AutoSolution®, latest version at time of this protocol amendment 1st November 2018). The present study will investigate the efficacy of lanreotide in NFMA, a new indication. In order to demonstrate a potential effect the use of a high dose is justified. Treatment with lanreotide autosolution 120 mg was shown to be safe and well-tolerated in two recent efficacy studies [5, 16]. A safety review on dose optimization of somatostatin analogues and a phase II RCT specifically investigating lanreotide autosolution (or Autogel) in different dosages did not demonstrate significant changes in adverse effects and tolerability with increasing dose. Higher doses were, however, related to greater efficacy [17, 18].

5.2 Use of co-intervention

Participants are allowed to use their regular prescription medication as well as non-prescription medicines and products, such as analgesics or vitamin supplements. Patients using dopamine agonists will be excluded from participation as described in chapter 4.3. The pharmacological gastrointestinal effects of lanreotide may result in reduced intestinal absorption of most notably ciclosporin. Patients using ciclosporin will be advised to have their ciclosporin level checked regularly and adjust the dose to maintain therapeutic levels, if necessary. Concomitant use of bradycardia inducing drugs (e.g. beta blockers) may have an additive effect on the slight reduction of heart rate associated with lanreotide. Dose adjustments of such concomitant medicines may be necessary. Participants shall be instructed to notify the research team if they have been prescribed a new drug by their general practitioner or specialist during the trial.
There are no dietary restrictions. With regard to fertile, female participants: pregnancy or plans to become pregnant during the study are exclusion criteria. Adequate contraception is essential.

5.3 Escape medication (if applicable)

Not applicable.

6. INVESTIGATIONAL PRODUCT

Lanreotide "Somatuline® AutoSolution®" 120 mg is a product with a marketing authorization (RVG 26303). The therapeutic indications for pituitary tumors are acromegaly and TSH-producing pituitary adenomas. The summary of product characteristics (SPC) is attached to the protocol, latest version 1st November 2018. A structured risk analysis including references is provided in chapter 13.1.

The placebo injections will consist of saline (sodium chloride 0.9%) and will be provided by the Hospital Pharmacy of the Academic Medical Centre (AMC).

The injections will either be administered by an endocrine nurse at the clinical Endocine Unit of the AMC (Department of Endocrinology and Metabolism), or at home by trained nurses from Eurocept Homecare.

6.1 Name and description of investigational product(s)

Lanreotide "Somatuline® AutoSolution®" 120 mg. Lanreotide is an octapeptide analogue of natural somatostatin. Each pre-filled syringe contains a supersaturated solution of lanreotide acetate corresponding to 0.246 mg of lanreotide base / mg of solution, which ensures an actual injection dose of 120 mg of lanreotide.

6.2 Summary of findings from non-clinical studies

See SPC and chapter 13.1.

6.3 Summary of findings from clinical studies

See SPC and chapter 13.1.

6.4 Summary of known and potential risks and benefits

See SPC and chapter 13.1.

6.5 Description and justification of route of administration and dosage

Lanreotide autosolution 120 mg or saline will be injected, via the deep subcutaneous route, into the superior, external quadrant of the buttock. The deep subcutaneous route of administration presents minimal discomfort to the subject and is the accepted route for administration in standard patient care. Treatment with lanreotide autosolution 120 mg was shown to be safe and well-tolerated as a first-line therapy in patients with growth hormone secreting pituitary adenomas (Caron et al., 2014).

6.6 Dosages, dosage modifications and method of administration

Lanreotide autosolution 120 mg will be provided in a pre-filled syringe. There will be no dosage modifications during this study. The method of administration has been described in chapter 6.5.

6.7 Preparation and labelling of Investigational Medicinal Product

Since lanreotide autosolution 120mg and saline differ in appearance, the study medication will be delivered by the Trial Pharmacy of the AMC in a sealed, opaque bag placed into a cardboard box with a warning message to maintain blinding for the investigators. Patient details will be shown on the

exterior of the bag, and a separate label will be provided inside the bag for the syringe (an example of the label is provided as a separate document). Lanreotide is delivered in a pre-filled syringe. The placebo syringe (with saline) will either be prepared at the day of administration by the unblinded endocrine nurse at the Endocrine Unit in case of administration at the AMC, or by the AMC Pharmacy in case of administration at home, no more than 7 days before administration is planned.

6.8 Drug accountability

Drug accountability will be performed by the Trial Pharmacy of the AMC (Kenniscentrum Geneesmiddelenonderzoek) according to GCP guidelines.

7. NON-INVESTIGATIONAL PRODUCT (NIMP)

A gadolinium-based contrast agent will be used during pituitary MRI. Since this procedure is part of standard clinical care the contrast-agent shall not be described here.
The radiopharmaceutical Gallium-68 DOTATATE will be used to perform a PET/CT. This scan will be performed only once, at the beginning of the study.

The PET/CT can be performed at either the AMC or the NKI-AvL; the Gallium-68 DOTATATE is produced on site according to GMPZ guidelines. It is performed as in standard clinical practice.

7.1 Name and description of non-investigational product(s)

Gallium-68 DOTATATE (also written as ^{68}Ga-DOTATATE) is a radiolabeled somatostatin analog. DOTATATE is an amide of the acid DOTA, which allows chelation with the PET-emitting metal ion Gallium-68, and (Tyr3)-octreotate, a derivative of octreotide. The latter binds to somatostatin receptors, with a predominant affinity for SSTR2. The pre-formulated GMP kit is used to prepare the final product, which is a ready-to-inject solution. The product is for diagnostic use only.

7.2 Summary of findings from non-clinical studies

Not applicable, the product is used as in standard clinical practice.

7.3 Summary of findings from clinical studies

Not applicable, the product is used as in standard clinical practice.

7.4 Summary of known and potential risks and benefits

Not applicable, the product is used as in standard clinical practice.

7.5 Description and justification of route of administration and dosage

Not applicable, the product is used as in standard clinical practice.

7.6 Dosages, dosage modifications and method of administration

The standard dose of Gallium-68 DOTATATE consists of 100 megabecquerel (MBq). Considering the estimated effective dose equivalent of 0.026 millisievert (mSv) per megabecquerel (MBq), this dose will amount to 2.6 mSv radiation exposure [19]. The combination of PET with low-dose CT will lead to a total effective dose of 3.1 mSv. The radiopharmaceutical is administered intravenously as a bolus injection, using a venous catheter to avoid extravasation. Based upon the pharmacokinetic properties, PET/CT can take place approximately 60 minutes after administration.

7.7 Preparation and labelling of Non Investigational Medicinal Products

Preparation and labelling of Gallium-68 DOTATATE will be performed according to standard protocol and current GMPZ guidelines at the AMC or the NKI-AvL. The respected pharmacies have been contacted for this. The preparation and imaging procedures are based on the recently published procedure guidelines by Virgolini et al. [20], which are supported by the European Association of Nuclear Medicine.

7.8 Drug accountability

Not applicable, the product is used as in standard clinical practice.

8 METHODS

8.1 Study parameters/endpoints

In patients with NFMA, a number of small open label studies have shown variable effects of treatment with somatostatin analogues on tumor size. In the present study we will perform an RCT in NFMA patients with positive pituitary somatostatin receptor imaging using Gallium-68 DOTATATE PET/CT. The hypothesis to be tested is that treatment with lanreotide autosolution 120 mg every 4 weeks during 18 months will decrease NFMA size as compared to placebo treatment.

8.1.1 Main study parameter/endpoint

The change in cranio-caudal NFMA size over 18 months.

Pituitary MRI is performed at baseline, at 6 months (week 24) and after 18 months of lanreotide autosolution 120 mg or placebo (week 72). The cranio-caudal size will be determined by 2 radiologists, who are blinded to treatment group and timing. Cranio-caudal size will be expressed in millimetres. The cranio-caudal size used for the primary endpoint will be the mean of the results of 2 radiologists. In case of a difference >2 mm between the 2 radiologists, the images will be offered for second review. In case of a persistent difference >2 mm, a 3rd radiologist will review the images and the average of 3 radiologists will be used for the primary endpoint.

8.1.2 Secondary study parameters/endpoints

1. Number of (serious) adverse events

Adverse events will be collected using a non-leading question at predefined time points (at time of the third injection around week 8 and at week 24, 48 and 72; see study procedures in Table 1, chapter 8.3) and at any time during the study in case of a serious adverse event. Handling of adverse events is described in chapter 9.2.

2. The change in quality of life

Quality of life will be assessed using a standardized questionnaire (SF-36), validated Dutch translation. The SF-36 Health Survey is a widely used and validated general health survey. It is composed of 36 questions, organized into eight scales: physical functioning, role limitations due to physical health problems, bodily pain, general health perceptions, vitality, social functioning, role limitations due to emotional problems, and general mental health [21]. Questionnaires will be completed at baseline, and at week 24, 48 and 72. The results will be compared with the normal population and interpreted according to the SF-36 Manual and Interpretation Guide.

3. The change in NFMA volume measured on pituitary 3DT1 MRI over 18 months.

A volumetric 3DT1 sequence has been added to the standard contrast-enhanced pituitary MRI at baseline , at 6 months (week 24) and 18 months (week 72). The images will be analyzed and NFMA volume determined by a qualified person who will be blinded to treatment group and timing. NFMA volume will be expressed in cubic millimeters (mm^3). Tumor shrinkage or growth of ≥25% will be considered clinically significant. A change of <25% is considered as stable disease [22, 23].

4. Time to tumor progression (i.e. tumor growth)
The presence or absence of tumor progression will be based on the change in tumor volume as described in secondary endpoint no. 3, measured between baseline and subsequent pituitary MRI's. Presence of tumor progression is defined as an increase in tumor volume of ≥25% , absence of tumor progression comprises both tumor shrinkage (volume decrease ≥25%) and stable disease (volume change of <25%).

8.1.3 Other study parameters

At entry (visit 1), all participants will undergo a brief physical examination and a medical history will be taken. The following data will be documented:

- Date of enrolment
- Study participant number
- Initials, date of birth, sex
- Medical history, family history
- Current and recent medication use
- Alcohol use, smoking
- Blood pressure, pulse rate, height, weight
- Auscultation of heart, lungs and abdominal examination

Apart from this data there will be documentation of laboratory test results obtained at baseline , week 24, 48 and 72. Laboratory tests comprise full blood count, HbA1c, glucose, liver enzymes, bilirubin, creatinine, electrolytes, TSH, free T4, prolactin, IGF-1, cortisol, testosterone or estradiol, gonadotropins, and alpha subunits (see Table 1. Study procedures).

8.2 Randomization, blinding and treatment allocation

We will randomly allocate all subjects to treatment with lanreotide autosolution 120 mg or with placebo injections based on randomization performed and provided by the Hospital Pharmacy (block randomization, 11 blocks of 4 patients). Lanreotide autosolution 120mg is provided in pre-filled syringes, which differs in appearance from placebo (saline 0,9%). It is unfortunately not possible to transfer the lanreotide to another syringe similar to a syringe with placebo due to stability issues of the lanreotide. To maintain the study blinding, the hospital pharmacy will provide the investigator with a sealed, opaque bag, placed in a cardboard box with a "blinded medication" warning message, containing lanreotide or saline, a protocol to prepare the syringe in case of placebo, and a label for the syringe. If administered at the AMC, the medication will be prepared, controlled and administered by an experienced, unblinded endocrine nurse at the Endocrine unit of the Department of Endocrinology and Metabolism of the AMC. If the injection is administered at home, the AMC Pharmacy will prepare the placebo and place the prepared syringe in the sealed bag before it is delivered at the patient's home at the day of the administration. The investigators will remain blinded, but the nurse who prepares and/or administers the medication will be aware of the nature of the medication. As the injection will be placed into the superior, external quadrant of the buttock, the patient will not see the

injection and therefore blinding of the patient is guaranteed. This procedure is similar to a recently published RCT with lanreotide in metastatic enteropancreatic neuroendocrine tumors (CLARINET, ClinicalTrials.gov number NCT00353496; EudraCT 2005-004904-35) [16]. The randomization code will be broken in case of a SUSAR (breaking of the code by the pharmacy will be requested by an independent trial staff member, to maintain blinding of the investigators).

8.3 Study procedures

Table 1. Study procedures

WEEK →	-2	-1	0 - 20	24	28 - 44	48	52 - 68	72
VISIT* → ↓ EVENT	1	(2)	x	2(3)	x	3(4)	x	4(5)
Injection number (each 28 days)			1-6	7	8-12	13	14-18	
In-/exclusion criteria	X	(X)						
Informed consent	X							
History	X							
Physical examination	X			X		X		X
Quality of Life	X			X		X		X
Laboratory[1]	X			X		X		X
Pregnancy test (if applicable)	X							
Pituitary MRI[2]	X			X				X
PET/CT[3]	X	(X)						
Adverse events			X	X	X	X	X	X
End of study (EOS)								X

↑ RANDOMIZATION

**the amount of study visits is dependent on the center of inclusion and on where the injections are administered. For AMC patients the PET/CT can be performed on visit 1, for VUmc and LUMC patients this will be done on visit 2. If all injections are administered at the AMC, the maximum amount of study visits is 21*

[1]full blood count, HbA1c, glucose, liver enzymes, bilirubin, creatinine, electrolytes, TSH, free T4, prolactin, IGF-1, cortisol, testosterone or estradiol, gonadotropins and alpha subunits
[2]pituitary MRI does not have to be performed is there is documentation that the procedure was performed within the previous three months and the results do not give the investigator cause to repeat it
[3]Gallium-68 DOTATATE PET/CT

History
At entry (visit 1, week -2), a complete history will be taken, including medical history, family history, current and recent medication use, alcohol use and smoking.

Questionnaires

At visit 1 (week -2), all participants will complete the SF-36 Health Survey to assess quality of life. This will be repeated at week 24, 48 and 72. The questionnaires are part of the study.

Physical examination

At visit 1 (week -2), all participants will undergo a brief physical examination, consisting of measurement of blood pressure, pulse rate, height and weight, auscultation of heart and lungs and abdominal examination. This will be repeated at week 24, 48 and 72. Regular physical examinations are part of the standard clinical care.

Laboratory tests

At visit 1 (week -2), a blood sample will be obtained to measure full blood count, glucose, liver enzymes, bilirubin, creatinine, electrolytes, TSH, free T4, prolactin, IGF-1, cortisol, testosterone or estradiol, gonadotropins, and alpha subunits (single venipuncture, 8 vials, 36 mL). Visit 1 will include a pregnancy test if applicable. Laboratory tests will be repeated at week 24, 48 and 72 and are part of the standard clinical care. Part of the serum and plasma obtained will be stored until publication.

Imaging procedures

- Pituitary MRI

At visit 1 (week -2) pituitary MRI will be performed unless there is documentation that the procedure was performed within the previous 3 months and the results do not give the investigator cause to repeat it. Pituitary MRI will consist of a contrast-enhanced sequence using a gadolinium-based contrast agent and a volumetric 3DT1 sequence. MRI will be repeated at week 24 and week 72. Pituitary MRI's are performed regularly as part of standard clinical care.

- Gallium-68 DOTATATE PET/CT of the head

Gallium-68 DOTATATE PET/CT will be performed at visit 1 for AMC based patients and visit 2 for VUmc and LUMC patients. It is performed only once and it is an extra procedure for this study. Patients will receive a venous catheter through which the radionuclide is administered (maximum 10 mL). The catheter is then flushed with 10 mL of saline and removed. After 60 minutes a PET scan is performed directly followed by a low-dose CT scan. The total procedure (including explanation, injection, resting, and scanning) lasts 90 minutes.

Subcutaneous injections

Lanreotide autosolution 120 mg or saline will be injected, via the deep subcutaneous route, into the superior, external quadrant of the buttock. The injections will be given every 4 weeks, either during a short visit at the AMC or at home. Injection number 7 and 13 will both be administered at the AMC, during the week 24 and week 48 study visit. The final visit takes place after the 18th and last injection. All injections will be administered by qualified and trained nurses, either at the AMC Endocrine unit or through Eurocept Homecare.

Adverse events

Adverse events can be reported throughout the study period and during the injections, and will be inquired about specifically at the study visits at week 24, week 48 and week 72.

8.4 Withdrawal of individual subjects

Subjects can leave the study at any time for any reason if they wish to do so without any consequences. The investigator can decide to withdraw a subject from the study for urgent medical reasons.

8.4.1 Specific criteria for withdrawal

The necessity of withdrawal from the study will be examined for each individual subject in case of any new condition during the study requiring hospital admission. If withdrawal is deemed necessary we plan to perform a pituitary MRI at the time of withdrawal in order to include the data in the intention-to-treat analysis, unless the previous MRI was performed within the last 4 weeks. Subjects who wish to leave the study for personal reasons will also be requested to undergo an MRI in order to determine the NFMA size at withdrawal, unless the previous MRI was performed within the last 4 weeks. Patients who develop visual field impairment or mydriasis, limitation of gaze, or ptosis (pointing to cranial nerve injury to CN II, III, VI or V) during the study will undergo MRI and visual field examination. If transsphenoidal surgery is deemed necessary the patient will be withdrawn from the study. In these patients, the cranio-caudal adenoma size at the time of MRI will also be included in the intention-to-treat analysis.

8.5 Replacement of individual subjects after withdrawal

In the updated sample size calculation, a drop-out/withdrawal percentage of ~27% is taken into account. Individual subjects will therefore not be replaced by new subjects. Data of all randomized subjects will be included in the intention-to-treat analysis (see section 10 for details on the analysis).

8.6 Follow-up of subjects withdrawn from treatment

If a subject decides to withdraw from the study, the investigator will ask for the reason. Withdrawal from the study will have no effect on the regular treatment. Subjects who leave the study for medical reasons will be followed until the interfering condition has resolved or reached a stable state.

8.7 Premature termination of the study

In case of multiple SUSARs the study will be terminated. This will be the responsibility of the principal investigator.

9 SAFETY REPORTING

9.1 Temporary halt for reasons of subject safety

In accordance to section 10, subsection 4, of the WMO, the AMC (sponsor) will suspend the study if there is sufficient ground that continuation of the study will jeopardize subject health or safety. The AMC will notify the accredited METC without undue delay of a temporary halt including the reason for such an action. The study will be suspended pending a further positive decision by the accredited METC. The investigator will take care that all subjects are kept informed.

9.2 AEs, SAEs and SUSARs

9.2.1 Adverse events (AEs)

Adverse events are defined as any undesirable experience occurring to a subject during the study, whether or not considered related to lanreotide treatment. All adverse events reported spontaneously by the subject or observed by the investigator or his staff will be recorded.

9.2.2 Serious adverse events (SAEs)

A serious adverse event is any untoward medical occurrence or effect that at any dose:

- results in death;
- is life threatening (at the time of the event);
- requires hospitalization or prolongation of existing inpatients' hospitalization;
- results in persistent or significant disability or incapacity;
- is a congenital anomaly or birth defect;
- any other important medical event that may not result in death, be life threatening, or require hospitalization, may be considered a serious adverse experience when, based upon appropriate medical judgment, the event may jeopardize the subject or may require an intervention to prevent one of the outcomes listed above.

An elective hospital admission will not be considered as a serious adverse event.

At the VUmc prof M.L. Drent and at the LUMC prof A.M. Pereira will be responsible for correct handling of SAEs and notifying the AMC. At the AMC, the other (principal) investigators will be responsible for correct handling of SAEs and for notifying the AMC.
The AMC will report the SAEs through the web portal *ToetsingOnline* to the accredited METC that approved the protocol, within 15 days after the AMC has first knowledge of the serious adverse events.

SAEs that result in death or are life threatening should be reported expedited. The expedited reporting will occur not later than 7 days after the responsible investigator has first knowledge of the adverse event. This is for a preliminary report with another 8 days for completion of the report.

9.2.3 Suspected unexpected serious adverse reactions (SUSARs)

Adverse reactions are all untoward and unintended responses to an investigational product related to any dose administered.

Unexpected adverse reactions are SUSARs if the following three conditions are met:

1. the event must be serious (see chapter 9.2.2);
2. there must be a certain degree of probability that the event is a harmful and an undesirable reaction to the medicinal product under investigation, regardless of the administered dose;
3. the adverse reaction must be unexpected, that is to say, the nature and severity of the adverse reaction are not in agreement with the product information as recorded in:
   - Summary of Product Characteristics (SPC) for an authorized medicinal product;
   - Investigator’s Brochure for an unauthorized medicinal product.

At the VUmc prof M.L. Drent and at the LUMC prof A.M. Pereira will be responsible for correct handling of SUSARs and notifying the AMC. At the AMC, the other (principal) investigators will be responsible for correct handling of SUSARs and for notifying the AMC.
The AMC will report expedited all SUSARs through the web portal *ToetsingOnline* to the METC. This includes:

– All SUSARs that have arisen in the clinical trial that was assessed by the METC;
– All SUSARs that have arisen in other clinical trials of the same sponsor and with the same medicinal product, and that could have consequences for the safety of the subjects involved in the clinical trial that was assessed by the METC.

The expedited reporting of SUSARs through the web portal *ToetsingOnline* is sufficient as notification to the competent authority.

To maintain the investigators blinded, an independent trial staff member is added and authorized in *ToetsingOnline* to report the event either as a SUSAR or a SAE, depending on treatment allocation (the code is only broken in case of well-founded suspicion of a SUSAR and approval of the Principal Investigator).

The AMC will report expedited all SUSARs to the competent authorities in other Member States, according to the requirements of the Member States.

The expedited reporting will occur not later than 15 days after the AMC has first knowledge of the adverse reactions. For fatal or life threatening cases the term will be maximal 7 days for a preliminary report with another 8 days for completion of the report.

9.3 Annual safety report

In addition to the expedited reporting of SUSARs, the AMC will submit, once a year throughout the clinical trial, a safety report to the accredited METC, competent authority, and competent authorities of the concerned Member States.

This safety report consists of:

- a list of all suspected (unexpected or expected) serious adverse reactions, along with an aggregated summary table of all reported serious adverse reactions, ordered by organ system, per study;
- a report concerning the safety of the subjects, consisting of a complete safety analysis and an evaluation of the balance between the efficacy and the harmfulness of the medicine under investigation.

9.4 Follow-up of adverse events

All AEs will be followed until they have abated, or until a stable situation has been reached. Depending on the event, follow up may require additional tests or medical procedures as indicated, and/or referral to the general physician or a medical specialist.

SAEs need to be reported until end of study within the Netherlands, as defined in the protocol.

9.5 Data Safety Monitoring Board (DSMB) / Safety Committee

Not applicable.

10. STATISTICAL ANALYSIS

All data will be analyzed using SPSS for Windows, version 21.0 or later if available (SPSS Inc. Chicago, USA). The data of categorical variables will be presented as incidence rates (number and percentage). The data of numerical variables will be presented as measures of central tendency (i.e., mean, median) and dispersion (i.e., standard deviation, range) or median interquartile range according to distribution. Differences between groups in numerical variables are evaluated using the student's unpaired t-test for normally distributed variables and the Mann–Whitney U test for not normally distributed parameters. The Chi-square test is used to analyze group differences in categorical variables. If the sample size is small or cells have an expected count <5, the Fisher exact test is used. When performing linear regression analyses, assumptions for linearity, distribution and homoscedasticity will be checked for each model using scatter plots and residual analysis. The statistical significance level for all analyses is set at P = 0.05 (two-sided) unless otherwise mentioned. In case of multiple testing, Bonferroni correction will be applied if appropriate. Data will be presented using tables and/or graphs.

Data on baseline clinical characteristics, adverse events and PET/CT results of all included subjects will be reported. Data of all subjects randomized for treatment will be analyzed according to intention-to-treat (ITT). In case of withdrawal of a subject after initiation of treatment, we aim to perform follow-up tests at time of withdrawal in order to obtain outcome data for the ITT analysis, unless those tests were performed within the previous 4 weeks. As withdrawn subjects will still undergo regular follow-up tests as part of standard care for their pituitary adenoma, these results can be included in the analyses if appropriate. In case of incomplete/missing outcome data, the assumption that the data is missing at random (MAR) will be checked. If the MAR assumption is plausible, a main analysis using all observed data is performed (e.g. a mixed model). This main analysis does not require the inclusion of subjects with no outcome measures (i.e. subjects that lack all follow-up tests after randomization) [24]. Sensitivity analyses are then performed to explore the impact of departures from the assumption made in the main analysis; these sensitivity analyses will include <u>all</u> randomized subjects [24]. This will include an analysis using multiple imputation in case of missing outcome data [25]. For the primary study endpoint and secondary endpoint no.3, a separate per-protocol (complete case) efficacy analysis is performed including only those subjects that have completed all 18 study injections.

10.1 Primary study parameter

The change in cranio-caudal NFMA size over 18 months.

The final analysis of the primary endpoint will be performed by one-way ANCOVA between groups, controlling for baseline adenoma size.

10.2 Secondary study parameters

1. Number of (serious) adverse events (AEs)

The data on AEs (categorical variables) will be analyzed and reported as described above.

2. The change in quality of life (SF-36 Health Survey)

The results of SF-36 will be compared with the normal population and interpreted according to the SF-36 Manual and Interpretation Guide. The mean imputation method will be used to replace missing values. The data (continuous variables) will be analyzed and reported as described above.

3. The change in NFMA volume measured on pituitary 3DT1 MRI over 18 months.

The analysis of this secondary endpoint will be performed by one-way ANCOVA (between groups) and one-way repeated measures ANOVA (within groups).

4. Time to tumor progression (i.e. tumor growth)

Time to progression is analyzed using the Kaplan-Meier method. Missing cases due to withdrawal without outcome data will be censored. Differences in distribution and percentage of censoring between the groups will be evaluated. Between-group differences in time to progression are analyzed using the stratified logrank test, with stratification for the presence or absence of tumor progression at baseline. To quantify the difference, the hazard ratio and confidence intervals are estimated with the use of the Cox proportional-hazards model.

10.3 Other study parameters

Baseline and clinical characteristics comprise categorical and continuous variables and will be analyzed and reported as described above.

10.4 Interim analysis (if applicable)

Not applicable.

11. ETHICAL CONSIDERATIONS

11.1 Regulation statement

This study will be conducted according to the principles of the Declaration of Helsinki (October 2013) and in accordance with the Medical Research Involving Human Subjects Act (WMO, March 2006) and the Dutch Good Clinical Practice guideline (March 2003).

11.2 Recruitment and consent

Participants will be recruited from the total population of adult (18 years or older) patients who are currently treated at the Outpatient Departments of Endocrinology in the AMC or in the VUmc for non-functioning pituitary adenoma (NFA) identified by a DBC screen. The treating physician at the Outpatient Department of Endocrinology will inform eligible patients about the study and will enable the patient to contact the investigators by providing their contact details. Patients who contact the principal investigator will receive the patient information letter and informed consent form. These forms are attached as a separate document. Subjects will be offered at least five days to consider their decision before written informed consent is obtained. Some of the treating physicians are part of the investigator team. There are, however, no commercial or financial interests or gains related to inclusion of patients that could lead to conflict of interest. Moreover, participation could prove beneficial to the subjects.

11.3 Objection by minors or incapacitated subjects (if applicable)

Not applicable.

11.4 Benefits and risks assessment, group relatedness

At present, there is no medical treatment option for patients with NFMA. The standard practice is, therefore, a wait-and-see approach to monitor growth of the pituitary tumor. The ideal timing for surgery is unclear. As tumor progression is often slow (0.6 mm/year in one series by Dekkers et al. [7]), remission rate after surgery is estimated to be only about 44%, and recurrence rate is about 11% during an average follow-up period of 5 years [1], surgery is often postponed until surgery is warranted because of the development of visual field defects or impaired visual acuity resulting from optical chiasm compression. If medical therapy is effective to reduce tumor volume, or to slow down tumor progression, this would represent a potentially important new treatment strategy obviating surgery in a substantial number of patients. In the present study, patients treated with lanreotide may experience tumor shrinkage, which may postpone or obviate surgery. Thus, the proposed research may be beneficial to the subject. In addition, if the hypothesis proves correct, larger studies could follow that may impact and improve standard treatment of NFMA in a major way.

During the study, the participants will undergo a Gallium-68 DOTATATE PET/CT and, if positive, they will be randomized to receive a 4-weekly deep subcutaneous injection of lanreotide or placebo during 18 months (18 injections). Patients can choose to receive the injections at home, limiting the amount

of study visits to the hospital and the burden of participation. The most commonly expected adverse drug reactions following treatment with lanreotide are gastrointestinal disorders (most commonly reported are diarrhea and abdominal pain, usually mild or moderate and transient), cholelithiasis (often asymptomatic) and injection site reactions (pain, nodules and indurations). Additionally, lanreotide, like somatostatin and other somatostatin analogues, inhibits the secretion of insulin and glucagon which may induce (mild) hypoglycaemia or hyperglycaemia. Because of the surveillance at regular intervals during the study the risks are small. The laboratory tests performed during the study are part of standard clinical care and the risks associated with venous blood sampling are negligible. The radiation exposure associated with Gallium-68 DOTATATE PET/CT is estimated at 3.1 mSv and presents a negligible risk.

11.5 Compensation for injury

The AMC has a liability insurance which is in accordance with article 7, subsection 9 of the WMO.

The AMC (also) has an insurance which is in accordance with the legal requirements in the Netherlands (Article 7 WMO and the Measure regarding Compulsory Insurance for Clinical Research in Humans of 23th June 2003). This insurance provides cover for damage to research subjects through injury or death caused by the study.

1. € 450.000,-- (i.e. four hundred and fifty thousand Euro) for death or injury for each subject who participates in the Research;
2. € 3.500.000,-- (i.e. three million five hundred thousand Euro) for death or injury for all subjects who participate in the Research;
3. € 5.000.000,-- (i.e. five million Euro) for the total damage incurred by the organization for all damage disclosed by scientific research for the Sponsor as ‘verrichter’ in the meaning of said Act in each year of insurance coverage.

The insurance applies to the damage that becomes apparent during the study or within 4 years after the end of the study.

11.6 Incentives (if applicable)

Participants will receive travel reimbursements during the study.

12 ADMINISTRATIVE ASPECTS, MONITORING AND PUBLICATION

12.1 Handling and storage of data and documents

All data will be handled confidentially and will be coded by a code known only to the investigator. The key to the code will be safeguarded by the investigator. A subject identification code list will be used to link the data to the subject. Data will be kept as long as needed for the analysis and publication in accordance with the FMVV Code of Conduct for Health Research. The data storage period of medical research is 15 years, in accordance with the Archiefwet 1995. Blood samples obtained during the study will be stored at the Laboratory of Endocrinology according with local regulatory guidelines (“spijtmateriaal”). These samples will be stored until publication (end of study).

12.2 Monitoring and Quality Assurance

Monitoring of the conduct of the study will take place internally. See the monitoring plan attached as a separate document.

12.3 Amendments

A ‘substantial amendment’ is defined as an amendment to the terms of the METC application, or to the protocol or any other supporting documentation, that is likely to affect to a significant degree:

- the safety or physical or mental integrity of the subjects of the trial;
- the scientific value of the trial;
- the conduct or management of the trial; or
- the quality or safety of any intervention used in the trial.

All substantial amendments will be notified to the METC and to the competent authority.

Non-substantial amendments will not be notified to the accredited METC and the competent authority, but will be recorded and filed by the sponsor.

12.4 Annual progress report

The sponsor/investigator will submit a summary of the progress of the trial to the accredited METC once a year. Information will be provided on the date of inclusion of the first subject, numbers of subjects included and numbers of subjects that have completed the trial, serious adverse events/serious adverse reactions, other problems, and amendments.

12.5 Temporary halt and (prematurely) end of study report

The sponsor will notify the accredited METC and the competent authority of the end of the study within a period of 90 days. The end of the study is defined as the last patient’s last visit.

The sponsor will notify the METC immediately of a temporary halt of the study, including the reason of such an action.

In case the study is ended prematurely, the sponsor will notify the accredited METC and the competent authority within 15 days, including the reasons for the premature termination.

Within one year after the end of the study, the investigator/sponsor will submit a final study report with the results of the study, including any publications/abstracts of the study, to the accredited METC and the competent authority.

12.6 Public disclosure and publication policy

The trial will be registered in a public trial registry before the first patient is recruited. The results of this study will be submitted for publication in international, peer-reviewed journals.

13 STRUCTURED RISK ANALYSIS

13.1 Potential issues of concern

a. Level of knowledge about mechanism of action

Lanreotide is a somatostatin receptor agonist, and is registered for the treatment of acromegaly and TSH-producing pituitary adenomas. There is no registration for NFMA. Although the majority of pathological NFMA specimens express somatostatin receptors [2], in vivo assessment of NFMA by Indium-111 pentetreotide SPECT yielded lower positive rates. This may be caused by insufficient sensitivity of SPECT. Octreotide, a somatostatin analogue, has been shown to reduce tumor size in ~12% of patients with NFMA [3]. However, the number of patients studied was small, and no randomized controlled trials (RCTs) have been published. Thus, somatostatin analogues may have potential in some NFMA patients, but there is a remarkable lack of randomized and controlled studies. We hypothesize that lanreotide may be effective to reduce NFMA size in patients selected on the basis of a positive Gallium-68 DOTATATE PET/CT scan of the pituitary, which is probably more sensitive to visualize somatostatin receptor expression than Indium-111 pentetreotide SPECT.

b. Previous exposure of human beings with the test product(s) and/or products with a similar biological mechanism

Treatment with lanreotide autosolution 120 mg was shown to be safe and well-tolerated as a first-line therapy in patients with growth hormone secreting pituitary adenomas [5].
Pharmacological studies in animals and humans show that lanreotide, like somatostatin and other somatostatin analogues, inhibits the secretion of insulin and glucagon. Hence, patients treated with lanreotide may experience mild hypoglycemia or hyperglycemia. Blood glucose and HbA1c levels will be monitored during the study and any anti-diabetic treatment will be adjusted accordingly. See also the attached SPC.

c. Can the primary or secondary mechanism be induced in animals and/or in *ex-vivo* human cell material?

In surgical specimens of pituitary tumors, somatostatin receptor expression can be assessed by immunocytochemistry, mRNA in situ hybridization, and pharmacological approaches [26]. These characteristics correlate in general with clinical response to somatostatin analogue treatment. However, this specific information is not available for NFMA.

d. Selectivity of the mechanism to target tissue in animals and/or human beings

Lanreotide has a high affinity for human somatostatin receptors (SSTR) 2 and 5, and a reduced binding affinity for human SSTR 1, 3 and 4. The somatostatin receptor has a wider tissue distribution, especially in the gut. This may explain gastrointestinal side effects that are frequently reported, see also the SPC.

e. Analysis of potential effect

See chapter 11.4 and SPC.

f. Pharmacokinetic considerations

Pharmacokinetic properties are known and are available [27], see also SPC.

g. Study population

The study will be performed in an outpatient setting. The patients are in a stable condition and are expected to show slow growth of the NFMA. Women in the child-bearing age will undergo a pregnancy test before inclusion.

h. Interaction with other products

For detailed description: see SPC.

Participants are allowed to use their regular prescription medication. The pharmacological gastrointestinal effects of lanreotide may result in reduced intestinal absorption of most notably ciclosporin. Patients using ciclosporin will be advised to have their ciclosporin level checked regularly and adjust the dose to maintain therapeutic levels, if necessary. Concomitant use of bradycardia inducing drugs (e.g. beta blockers) may have an additive effect on the slight reduction of heart rate associated with lanreotide. Dose adjustments of such concomitant medicines may be necessary.

Participants shall be instructed to notify the research team if they have been prescribed a new drug by their general practitioner or specialist during the trial.

i. Predictability of effect

The change in cranio-caudal NFMA size over 18 months will be assessed by MRI. This parameter is the main determinant of necessity for surgery as suprasellar extension of the NFMA towards the optic chiasm may cause visual field defects and/or decreased visual acuity.

j. Can effects be managed?

Not applicable

13.2 Synthesis

In this study the investigational product is a registered product for a variety of pituitary adenomas including TSH-producing pituitary adenomas and acromegaly. Treatment with lanreotide autosolution 120 mg is a safe and well-tolerated first-line therapy in patients with growth hormone secreting pituitary adenomas. In the present study we will perform a Gallium-68 DOTATATE PET/CT and, if positive (n=44), patients will be randomized to treatment with either lanreotide autosolution 120 mg or placebo for 18 months. The most common side effects of lanreotide are gastrointestinal disorders (most commonly reported are diarrhea and abdominal pain, usually mild or moderate and transient), cholelithiasis (often asymptomatic) and injection site reactions (pain, nodules and indurations). After completing the study, we expect that patients treated with

lanreotide will show reduced tumor progression compared with placebo treated patients. Many, but not all, patients with NFMA will undergo surgical treatment at some point. Thus, patients treated with lanreotide in the present study may benefit by postponing or obviating surgical treatment. In addition, if the trial shows a positive effect of lanreotide, many more patients may benefit from medical instead of surgical treatment. We therefore feel that we do not expose the participants to an unacceptable risk of side effects. Moreover, we do not deprive them of any standard therapy.

14 REFERENCES

1. Roelfsema F, Biermasz NR, Pereira AM (2012) Clinical factors involved in the recurrence of pituitary adenomas after surgical remission: A structured review and meta-analysis. Pituitary 15:71–83. https://doi.org/10.1007/s11102-011-0347-7
2. de Bruin TW, Kwekkeboom DJ, Van 't Verlaat JW, et al (1992) Clinically nonfunctioning pituitary adenoma and octreotide response to long term high dose treatment, and studies in vitro. J Clin Endocrinol Metab 75:1310–7. https://doi.org/10.1210/jcem.75.5.1430093
3. Colao A, Di Somma C, Pivonello R, et al (2008) Medical therapy for clinically non-functioning pituitary adenomas. Endocr Relat Cancer 15:905–15. https://doi.org/10.1677/ERC-08-0181
4. Kabasakal L, Demirci E, Ocak M, et al (2012) Comparison of 68Ga-DOTATATE and 68Ga-DOTANOC PET/CT imaging in the same patient group with neuroendocrine tumours. Eur J Nucl Med Mol Imaging 39:1271–1277. https://doi.org/10.1007/s00259-012-2123-y
5. Caron PJ, Bevan JS, Petersenn S, et al (2014) Tumor shrinkage with lanreotide autogel 120 mg as primary therapy in acromegaly: Results of a prospective multicenter clinical trial. J Clin Endocrinol Metab 99:1282–1290. https://doi.org/10.1210/jc.2013-3318
6. Dekkers OM, Pereira AM, Romijn JA (2008) Treatment and follow-up of clinically nonfunctioning pituitary macroadenomas. J Clin Endocrinol Metab 93:3717–3726. https://doi.org/10.1210/jc.2008-0643
7. Dekkers OM, Hammer S, de Keizer RJW, et al (2007) The natural course of non-functioning pituitary macroadenomas. Eur J Endocrinol 156:217–224. https://doi.org/10.1530/eje.1.02334
8. Molitch ME (2014) Nonfunctioning pituitary tumors. In: Fliers E, Korbonits M, Romijn JA (eds) Clinical Neuroendocrinology. Handbook of Clinical Neurology Series, Volume 124, 3rd ed. Elsevier, pp 167–184
9. Greenman Y, Cooper O, Yaish I, et al (2016) Treatment of clinically nonfunctioning pituitary adenomas with dopamine agonists. Eur J Endocrinol 175:63–72. https://doi.org/10.1530/EJE-16-0206
10. Plöckinger U, Reichel M, Fett U, et al (1994) Preoperative octreotide treatment of growth hormone-secreting and clinically nonfunctioning pituitary macroadenomas: effect on tumor volume and lack of correlation with immunohistochemistry and somatostatin receptor scintigraphy. J Clin Endocrinol Metab 79:1416–1423. https://doi.org/10.1210/jcem.79.5.7962337
11. Broson-Chazot F, Houzard C, Ajzenberg C, et al (1997) Somatostatin receptor imaging in somatotroph and non-functioning pituitary adenomas: correlation with hormonal and visual responses to octreotide. Clin Endocrinol (Oxf) 47:589–98
12. Oppizzi G, Cozzi R, Dallabonzana D, et al (1998) Scintigraphic imaging of pituitary adenomas: an in vivo evaluation of somatostatin receptors. J Endocrinol Invest 21:512–519
13. de Herder WW, Kwekkeboom DJ, Feelders RA, et al (2006) Somatostatin receptor imaging for neuroendocrine tumors. Pituitary 9:243–248. https://doi.org/10.1007/s11102-006-0270-5
14. Sharma P, Singh H, Bal C, Kumar R (2014) PET/CT imaging of neuroendocrine tumors with (68)Gallium-labeled somatostatin analogues: An overview and single institutional experience from India. Indian J Nucl Med 29:2–12. https://doi.org/10.4103/0972-3919.125760
15. Borm GF, Fransen J, Lemmens WAJG (2007) A simple sample size formula for analysis of covariance in randomized clinical trials. J Clin Epidemiol 60:1234–8. https://doi.org/10.1016/j.jclinepi.2007.02.006
16. Caplin ME, Pavel M, Cwikła JB, et al (2014) Lanreotide in metastatic enteropancreatic neuroendocrine tumors. N Engl J Med 371:224–33. https://doi.org/10.1056/NEJMoa1316158
17. Ludlam WH, Anthony L (2011) Safety review: Dose optimization of somatostatin analogs in patients with acromegaly and neuroendocrine tumors. Adv Ther 28:825–841. https://doi.org/10.1007/s12325-011-0062-9

18. Bronstein M, Musolino N, Jallad R, et al (2005) Pharmacokinetic profile of lanreotide Autogel?? in patients with acromegaly after four deep subcutaneous injections of 60, 90 or 120 mg every 28 days. Clin Endocrinol (Oxf) 63:514–519. https://doi.org/10.1111/j.1365-2265.2005.02372.x
19. Walker RC, Smith GT, Liu E, et al (2013) Measured human dosimetry of {68Ga-DOTATATE}. J Nucl Med 54:855–860. https://doi.org/10.2967/jnumed.112.114165
20. Virgolini I, Ambrosini V, Bomanji JB, et al (2010) Procedure guidelines for PET/CT tumour imaging with 68Ga-DOTA- conjugated peptides: 68Ga-DOTA-TOC, 68Ga-DOTA-NOC, 68Ga-DOTA-TATE. Eur J Nucl Med Mol Imaging 37:2004–2010. https://doi.org/10.1007/s00259-010-1512-3
21. Aaronson NK, Muller M, Cohen PDA, et al (1998) Translation, validation, and norming of the Dutch language version of the SF-36 Health Survey in community and chronic disease populations. J Clin Epidemiol 51:1055–1068. https://doi.org/10.1016/S0895-4356(98)00097-3
22. Therasse P, Arbuck SG, Eisenhauer EA, et al (2000) New guidelines to evaluate the response to treatment in solid tumors. J Natl Cancer Inst 92:205–216. https://doi.org/10.1093/jnci/92.3.205
23. Schalin-Jäntti C, Valanne L, Tenhunen M, et al (2010) Outcome of fractionated stereotactic radiotherapy in patients with pituitary adenomas resistant to conventional treatments: A 5.25-year follow-up study. Clin Endocrinol (Oxf) 73:72–77. https://doi.org/10.1111/j.1365-2265.2009.03755.x
24. White IR, Carpenter J, Horton NJ (2012) Including all individuals is not enough: Lessons for intention-to-treat analysis. Clin Trials 9:396–407. https://doi.org/10.1177/1740774512450098
25. Sterne JAC, White IR, Carlin JB, et al (2009) Multiple imputation for missing data in epidemiological and clinical research: potential and pitfalls. BMJ 338:b2393. https://doi.org/10.1136/bmj.b2393
26. Hofland LJ, van der Hoek J, Feelders RA, et al (2005) Pre-clinical and clinical experiences with novel somatostatin ligands: advantages, disadvantages and new prospects. J Endocrinol Invest 28:36–42
27. Kuhn JM, Legrand A, Ruiz JM, et al (1994) Pharmacokinetic and pharmacodynamic properties of a long-acting formulation of the new somatostatin analogue, lanreotide, in normal healthy volunteers. Br J Clin Pharmacol 38:213–9
