# Supplementary material for: Lanreotide versus placebo for tumour reduction in patients with a 68Ga-DOTATATE PET-positive, clinically non-functioning pituitary macroadenoma (GALANT study): a randomised, multicentre, phase 3 trial with blinded outcome assessment
Source: Lancet Reg Health Eur. 2024 May 13;42:100923. doi: 10.1016/j.lanepe.2024.100923 (PMC11281922; doi:10.1016/j.lanepe.2024.100923)
Supplement: Statistical Analysis Plan GALANT trial [file mmc3.pdf]

Statistical analysis plan (SAP) for:

A randomized placebo-controlled study in patients with a Gallium-68 DOTATATE PET/CT positive, clinically non-functioning pituitary macroadenoma (NFMA) of the effect of Lanreotide autosolution on Tumor (adenoma) size (GALANT)

Version 2.0, 16 February 2023

Aligned with Protocol version 5.0, 30 November 2018, and the published version (doi 10.1136/bmjopen-2020-038250)

Ethics committee number: METC 2015_103
Protocol number: NL52821.018.15
Netherlands Trial Registry: NL5136
EudraCT: 2015-001234-22

Section 1a. Title.
**What is the title of the statistical analysis plan?**
Statistical analysis plan for: A randomized placebo-controlled study in patients with a Gallium-68 DOTATATE PET/CT positive, clinically non-functioning pituitary macroadenoma (NFMA) of the effect of Lanreotide autosolution on Tumor (adenoma) size (GALANT)

Section 1b. Names and Signatures.

What are the names, affiliations and roles of contributors to this statistical analysis plan?

Role of contributor	Name and full affiliation*	Signature	Date of signature
**Principal investigator**	Prof. E. Fliers[1]		21/2/2023
**Researcher who will perform the statistical analysis**	Drs. T.M. Boertien[1]		21-2-2023
**Senior statistician consulted**	M.W.T. Tanck[2]		21-2-2023
**Contributor to statistical analysis plan**	Prof. P.H. Bisschop[1]		21-2-23

***Affiliations:**

[1] Amsterdam UMC location University of Amsterdam, Department of Endocrinology and Metabolism, Meibergdreef 9, Amsterdam, The Netherlands

[2] Amsterdam UMC location University of Amsterdam, Department of Epidemiology and Data Science, Meibergdreef 9, Amsterdam, The Netherlands

Section 1c. Revision history of the statistical analysis plan.

What versions of the statistical analysis plan have been approved and filed and what was the reason for producing each version?

Updated statistical analysis plan version	Protocol version	Section number(s) changed	Description of and reason for changes	Date of approval
1.0	5.0	NA	Increased sample size following observed dropout rate of ~25%. Plus a more detailed statistical analysis section in study protocol (as kick-off for first SAP version).	10-FEB-2019
2.0	5.0	 <br>Section 1d<br>Section 5.4<br>Section 6<br>Section 7.2/7.3	Finalisation of SAP before export of unblinded data from the database and start of outcome analyses.<br>Reporting of date of last visit of last patient.<br>Information on final dropout rate added.<br>Further details on statistical analyses added.<br>Reference to latest version of data management plan.	16-FEB-2023

Section 1d. Administrative Information.
**1.1. What is the trial registration number?**
This study is registered in the Netherlands Trial Register under reference NL5136, and with EudraCT under reference 2015-001234-22.
**1.2. What is the planned period of observation?**
Inclusion of the first patient took place on 3 November 2015.<br>Last visit of the last patient took place on 26 May 2021.
**1.3. What is the date and version number of the current statistical analysis plan?**
This statistical analysis plan is version 2.0 dated 16 February 2023.
**1.4. What is the date, version number and reference number of the protocol used when writing this statistical analysis plan?**
This statistical analysis plan is based on the protocol with reference number NL52821.018.15 version 5.0 dated 30-NOV-2018.

Section 2. Introduction.

2.1. What is the background and rationale for the study?

Despite advances in surgical techniques, the remission rate of transsphenoidal surgery in patients with clinically non-functioning pituitary macroadenoma (NFMA) remains around 50% and there is a high propensity for regrowth during follow-up. Currently, no established medical treatment options exist for patients with a large postoperative remnants or with macroadenomas with no immediate indication for resection. The expression of somatostatin receptors may provide a target for treatment with somatostatin analogues to reduce tumour size, but randomised controlled trials are lacking. In vivo somatostatin receptor assessment with ^{68}Ga-DOTATATE PET could help in selecting patients for treatment. We aimed to determine the effect of the somatostatin analogue lanreotide on tumour size in patients with a ^{68}Ga-DOTATATE PET-positive NFMA.

For further details on the background and rationale of this study see pages 10-12 (paragraph 1) of the protocol.

2.2. What are the objectives of the study?

The primary hypothesis is that lanreotide is superior to placebo in reducing tumour size (or growth) in patients with a ^{68}Ga-DOTATATE PET-positive NFMA.

Primary objective: to compare the change in cranio-caudal NFMA size over 72 weeks of treatment between treatment groups of lanreotide autosolution 120 mg and placebo.
- primary efficacy variable: absolute change in cranio-caudal tumour diameter in millimetres from baseline to week 72 (or last available post-baseline measurement).

Secondary Objectives:
1. To compare the change in NFMA tumour volume over 72 weeks of treatment between treatment groups.
2. To compare time to significant tumour progression (i.e. tumour growth) between treatment groups.
3. To compare the change in quality of life using a standardized questionnaire (SF-36) between treatment groups.
4. To assess the safety of lanreotide use in NFMA based on the number of (serious) adverse events.

Section 3. Study Methods.

3.1. What is the study design?

The GALANT study is an investigator-initiated, multicentre, randomized, double-blind, placebo-controlled, parallel-group, phase 3 trial. There are two treatment arms: “lanreotide” and “placebo”, both are administered as deep subcutaneous injections every 28 days for 72 weeks. The allocation ratio is 1:1.

For further details see page 13 (paragraph 3) and pages 22-24 (paragraph 8.3) of the protocol.

3.2. Will randomization be performed in this study?

A total of 44 patients with a ^{68}Ga-DOTATATE PET-positive NFMA are randomised without stratification by means of a computer-generated randomisation list through Sealed Envelope Ltd (https://www.sealedenvelope.com/simple-randomiser/v1/lists) using blocked randomisation with an allocation ratio of 1:1 and block size of four. The randomisation list is stored centrally in a secure trial file at the independent Trial Pharmacy of the Amsterdam UMC, location AMC, and will be disclosed after database lock via a written request and confirmation by the principal investigator.

Treating physicians, participants, investigators, and outcome assessors are blinded to treatment allocation. Because the placebo injections differ in appearance from the lanreotide (Somatuline AutoSolution®) pre-filled syringes, pharmacy employees preparing the treatment and nurses administering the injections cannot be blinded. Blinding during transport and storage is maintained by placement of the study medication in an opaque bag within a sealed cardboard box with a “blinded study medication” warning message. Participants will not see the syringes as these are administered into the superior, external quadrant of the buttock. Furthermore, participants have no earlier experience with lanreotide treatment to have expectations concerning the administration.

3.3. How was the sample size calculated?

The study is powered on a between-group difference in primary outcome (change in cranio-caudal diameter from baseline to end-of-treatment) of ≥2 mm. This value is considered clinically relevant, as such a difference is sufficient to prevent complications related to upwards tumour growth. Even a small increase in cranio-caudal diameter can be reason for intervention via surgery or radiotherapy to prevent or relieve compression of the optic nerves or chiasm.[1] Moreover, the value of 2 mm is commonly used as cut-off to reliably detect a change in tumour size on consecutive pituitary MRI.[2-4] In order to detect a mean difference of 2 mm with an estimated standard deviation of 1.9 mm (based on MRI resolution with use of 3 mm slice thickness sequences with 1 mm in-plane resolution), we need to randomise 16 patients per group based on a 2-sided independent *t*-test with 80% power and 5% type I error risk. The sample size calculation was performed with nQuery Advisor version 7.0 (Statsol, Boston, Massachusetts, USA).

In a substantial amendment to the protocol, the sample size was corrected for an observed dropout rate of 27%; this was implemented in the current protocol version 5.0 dated 30-NOV-2018.

The final analysis of the primary endpoint will be performed by ANCOVA. While ANCOVA may reduce the required sample size, a reliable estimation of rho (ie, correlation between covariate baseline tumour size and outcome end-of-treatment tumour size) is not possible for this study and a more conservative *t*-test-based power analysis is therefore less likely to result in an underpowered study.[5]

Only patients with a ^{68}Ga-DOTATATE PET-positive NFMA are randomised for treatment. Based on previous reports that about two-thirds of NFMA patients showed increased adenoma uptake using

^{111}In-DTPA-octreotide scintigraphy[6-9] and considering the superior sensitivity of ^{68}Ga-DOTATATE PET/CT[10], we expected to enrol a maximum of 66 patients in order to randomise 44 patients.

References
[1] Molitch ME. Diagnosis and Treatment of Pituitary Adenomas. *JAMA* 2017;**317**:516. doi:10.1001/jama.2016.19699
[2] Dekkers OM, Hammer S, de Keizer RJW, et al. The natural course of non-functioning pituitary macroadenomas. Eur J Endocrinol 2007;156:217–24. doi:10.1530/eje.1.02334
[3] O'Sullivan EP, Woods C, Glynn N, et al. The natural history of surgically treated but radiotherapy-naïve nonfunctioning pituitary adenomas. Clin Endocrinol (Oxf) 2009;71:709–14. doi:10.1111/j.1365-2265.2009.03583.x
[4] Brochier S, Galland F, Kujas M, et al. Factors predicting relapse of nonfunctioning pituitary macroadenomas after neurosurgery: A study of 142 patients. Eur J Endocrinol 2010;163:193–200. doi:10.1530/EJE-10-025
[5] Borm GF, Fransen J, Lemmens WAJG. A simple sample size formula for analysis of covariance in randomized clinical trials. *J Clin Epidemiol* 2007;**60**:1234–8. doi:10.1016/j.jclinepi.2007.02.006
[6] Cortet-Rudelli C, Bonneville J-F, Borson-Chazot F, *et al.* Post-surgical management of non-functioning pituitary adenoma. *Ann Endocrinol (Paris)* 2015;**76**:228–38. doi:10.1016/j.ando.2015.04.003
[7] Plöckinger U, Bäder M, Hopfenmüller W, *et al.* Results of somatostatin receptor scintigraphy do not predict pituitary tumor volume- and hormone-response to ocreotide therapy and do not correlate with tumor histology. *Eur J Endocrinol* 1997;**136**:369–76.http://www.ncbi.nlm.nih.gov/pubmed/9150695
[8] Borson-Chazot F, Houzard C, Ajzenberg C, *et al.* Somatostatin receptor imaging in somatotroph and non-functioning pituitary adenomas: correlation with hormonal and visual responses to octreotide. *Clin Endocrinol (Oxf)* 1997;**47**:589–98. doi:10.1046/j.1365-2265.1997.3361119.x
[9] Duet M, Ajzenberg C, Benelhadj S, *et al.* Somatostatin receptor scintigraphy in pituitary adenomas: a somatostatin receptor density index can predict hormonal and tumoral efficacy of octreotide in vivo. *J Nucl Med* 1999;**40**:1252–6.http://www.ncbi.nlm.nih.gov/pubmed/10450674
[10] Reubi JC, Schaer J-C, Waser B, Wenger S, Heppeler A, Schmitt JS, et al. Affinity profiles for human somatostatin receptor subtypes SST1-SST5 of somatostatin radiotracers selected for scintigraphic and radiotherapeutic use. Eur J Nucl Med Mol Imaging. 2000;**27**:273–82.

3.4. What is the hypothesis testing framework for this study?

The GALANT trial uses a superiority hypothesis testing framework for the primary and secondary outcomes. Regarding the outcome of safety based on adverse events: these will be summarised descriptively without formal hypothesis testing.

3.5. Will interim analyses be performed in this study?

No interim analyses will be performed.
There are no statistical guidelines for stopping the study early. In case of multiple SUSARs (suspected unexpected serious adverse reactions) the study will be terminated prematurely. This will be the responsibility of the principal investigator.

3.6. When will the final statistical analysis of the study data be performed?

All outcomes will be analysed collectively at the end of the study, after all outcome data has been entered into the database and unblinding can take place. Unblinded tumour size outcome assessment will take place centrally (Amsterdam UMC, location AMC) by two independent MRI outcome assessors. This will take place after the last visit of the last patient, to enable coding of all scan dates and ensure additional blinding of the outcome assessors to scan chronology. Regarding calendar time: MRI outcome assessments are estimated to take at least 6 months due to training and other (clinical) duties.

The current SAP version is an update just before data export and analysis of results.

3.7. At which time points are the outcomes measured and which "windows" are allowed?

An outline of the study is provided in the protocol (paragraph 8.3, page 22), and in the protocol publication (figure 3) [11]. The time points at which outcomes are measured are:
- at baseline (before first study injection)
- at week 24 (after the 6th study injection)
- at week 48 (after the 12th study injection – this visit does not include pituitary MRI)
- at week 72 (after the 18th and final study injection)

Pituitary MRI is only repeated at the baseline visit if time between the most recent MRI and planned start of study treatment exceeds three months or if the results provide any reason to repeat it.

Post-baseline visit windows have not been specifically defined in the protocol, but in practice the aim is a maximum of seven days before or after the nominally planned visit/injection, and deviations exceeding this window are recorded. The allowed injection window of 28 ±7 days has been recorded in a 'Protocol deviation on study level' form, dated 15-dec-2017.

Besides the prespecified study visits, effort is made to perform a premature end-of-study visit at time of withdrawal, unless assessments have been performed the four weeks before.

References
[11] Boertien TM, Drent ML, Booij J, *et al.* The GALANT trial: study protocol of a randomised placebo-controlled trial in patients with a 68Ga -DOTATATE PET-positive, clinically non-functioning pituitary macroadenoma on the effect of lanreotide on tumour size. *BMJ Open* 2020; **10**: e038250.

Section 4. Statistical Principles.

4.1. Which level or levels of statistical significance will be used in the study?

Primary and secondary outcomes will be viewed as statistically significantly different between treatment groups if the two-sided p-value is less than 0.05.

4.2. Will the analysis adjust for multiplicity of statistical testing to ensure control of type I error rate?

As there is one primary outcome measured at a single time point in this study, the analysis will not adjust for multiplicity of statistical testing. Regarding supplementary or sensitivity analyses (i.a. the per-protocol and mixed model analyses) and the secondary outcome analyses: no multiplicity adjustments are required as per usual [12].

References
[12] Li G, Taljaard M, Heuvel ER Van Den, Levine MAH, Cook DJ, Wells GA, Devereaux PJ, & Thabane L. An introduction to multiplicity issues in clinical trials: The what, why, when and how. *International Journal of Epidemiology* 2017 **46** 746–756. (doi:10.1093/ije/dyw320)

4.3. Which confidence intervals will be reported?

95% confidence intervals around point estimates will be presented for all outcome parameters.

4.4. How is compliance defined and assessed?

Compliance is defined as the number of study injections received by a participant in relation to the total number injections planned at time of study completion or study dropout. All study injections are administered by trained nurses and each administration is logged, thus adherence is ensured and not participant-dependent. In case of a deviation of the date of a planned injection, the schedule for the following injections remains unchanged and no injections should be skipped. The injection logs of all participants are checked at treatment completion for irregularities.

4.5. How will compliance be presented?

As adherence is expected to be 100% following logged administration by trained nurses, numbers/proportions regarding compliance are not planned to be presented in the report. In case of treatment irregularities/skipped injections this will be reported descriptively.

4.6. What are defined as protocol deviations in this study?

Protocol deviations are defined as: deviations from the eligibility criteria (including concomitant use of medication aimed at tumour reduction); deviations in the study time-line (eg, baseline MRI >3 months before start of study medication or injection/visit >7 days from original planned date); or the unblinding of patients to the allocated study medication.

Unblinding of a participant by an independent trial staff member in case of a SUSAR (suspected unexpected serious adverse reactions) is not regarded as a protocol violation.

4.7. How will protocol deviations be presented in the reporting of this study?
Protocol deviations will be summarised descriptively in the manuscript or supplementary files.
**4.8. Which analysis populations will be defined?**
The intention-to-treat population includes all randomised patients who have received at least one study injection.<br>The per-protocol population includes only those subjects who have completed all 18 study injections and week 72 MRI (deviations in time windows are allowed).<br>The safety population includes all randomised patients who have received at least one study injection (same population as intention-to-treat).

Section 5. Study populations.

5.1. Which data were collected from participants, who were screened for eligibility for inclusion in the study, and how these data will be presented in study reports?

Study reports will include an overview (eg, flowchart) of the number of potentially eligible patients who were approached for informed consent, and the reasons for not participating. Due to privacy restrictions, no data were collected from patients who were screened for eligibility or informed on the study but who did not provide informed consent.

Data of patients who were enrolled in the study but not randomised to treatment due to negative ^{68}Ga-DOTATATE PET-CT will be presented (table format), this will include data on i.a. age, sex, NFMA size and PET uptake.

5.2. What are the inclusion and exclusion criteria for the study?

Adult patients (≥18 years) diagnosed with an NFMA with suprasellar extension, either surgery-naïve or with a postoperative remnant, are eligible for inclusion. Patients are excluded in case of previous or planned radiotherapy in the pituitary region, known hypersensitivity to somatostatin, previous use of somatostatin analogues, use of dopamine receptor agonists in the six months before enrolment, current optic chiasm compression with visual field defects, symptomatic cholelithiasis, known diagnosis of obstructive neuroendocrine gut tumour, pregnancy or wish to conceive, contraindication to perform MRI, or inability to provide informed consent.

Inclusion and exclusion criteria are also detailed in the study protocol in paragraph 4.1, 4.2 and 4.3 on page 14-15. Note that herein the inclusion criterion of positive PET-CT is listed; this scan is however part of the study and is performed after enrolment. Patients with negative uptake are not randomised for treatment, as part of the protocol.

5.3. Which information will be presented in the flow chart for this study?

The flowchart of the study will include information on numbers of patients assessed for eligibility, numbers enrolled and randomised, number of withdrawals per treatment arm with reasons, and number included in the (intention-to-treat) analysis.

The mock-up of the CONSORT flow diagram is presented in the appendix to this statistical analysis plan (figure 1).

5.4. What is the expected level of, timing of and reasons for withdrawal from the intervention and/or from follow-up and how will this be presented in the study reports?

In the course of the trial, there was an observed dropout rate of ±25%. This was cause for a substantial amendment to the protocol to increase the sample size from 32 to 44 participants in order to maintain sufficient power (protocol version 5.0). The most expected reasons for withdrawal were adverse events and need for intervention following tumour progression. Timing of withdrawal was not predictable (although lanreotide-related AEs may present earlier in the trial).

At completion of the trial, the overall dropout rate was 27% (12/44 participants). In all participants, study outcomes (including pituitary MRI) were assessed at time of withdrawal. No patients were lost to follow-up or had withdrawn informed consent.

We will report the number and percentage of withdrawals in each treatment arm, and the timing of and reasons for study discontinuation.

5.5. Which baseline characteristics of participants will be presented?

Baseline characteristics to be presented:
- Age at enrolment
- Sex
- Relevant medical history (eg, diabetes mellitus)
- NFMA-related history: time since diagnoses, previous treatment, pituitary hormone deficiencies
- Baseline NFMA cranio-caudal tumour diameter and tumour volume
- ^{68}GA-DOTATATE PET uptake (SUV)

5.6. How will the baseline characteristics be summarized?

Data of categorical variables will be presented as incidence rates (number and percentage).
Data of numerical variables will be presented as measures of central tendency (i.e., mean, median) and dispersion (i.e., standard deviation, range) or median interquartile range according to distribution.

As this is a randomised clinical trial, no formal statistical tests will be performed on (chance) differences in baseline characteristics between treatment groups.

Section 6. Analysis.

6.1. How are the outcomes of this study defined?

The primary outcome is the absolute change in cranio-caudal tumour diameter in millimetres from baseline to week 72 or treatment discontinuation/last available post-baseline measurement. Methods of MRI measurement are detailed in the published the study protocol [11]. As additional information: measurements were done in the sagittal plane on the gadolinium-enhanced T1-weighted sequence and the maximum height of the tumour at the location of the sella was measured, using ITK-SNAP (versions 3.8.0, Philadelphia, PA, USA; www.itksnap.org).

There is no ordering in secondary outcomes. Secondary outcomes are:
- The absolute change in tumour volume in cubic millimetres from baseline to week 72 or treatment discontinuation/last available post-baseline measurement. As additional information to details listed in the published study protocol: measurements were done on the gadolinium-enhanced 3DT1-weighted sequence using a semi-automatic segmentation tool in ITK-SNAP, with post-segmentation manual slice-by slice adjustment. In case a volumetric sequence was missing for one of the participant's scans, all measurements for that participant were done on the regular gadolinium-enhanced T1-weighted images via multiplane slice-by-slice manual tumour segmentation.
- Time to tumour progression, based on the interval between start of study treatment and the first subsequent MRI showing a clinically significant increase in tumour volume of ≥20% (note: the percentage change regarded as clinically significant is correctly specified in the published protocol as ≥20%, with reference to Caron *et al.* [13], while the latest protocol version lists ≥25% as clinically significant).
- The change in quality of life based on change in SF-36 questionnaire component scores from baseline to week 72 or treatment discontinuation/last available post-baseline measurement.
- Safety in the form of number and type of (serious) adverse events, collected throughout the study from enrolment to 30 days after the last study injection. Adverse events will be assessed specifically at each study visit (through semi-structured interview, short physical exam and laboratory tests), and can be reported at any time during study participation.

References
[11] Boertien TM, Drent ML, Booij J, *et al.* The GALANT trial: study protocol of a randomised placebo-controlled trial in patients with a 68Ga -DOTATATE PET-positive, clinically non-functioning pituitary macroadenoma on the effect of lanreotide on tumour size. *BMJ Open* 2020; **10**: e038250.
[13] Caron PJ, Bevan JS, Petersenn S, *et al.* Tumor Shrinkage With Lanreotide Autogel 120 mg as Primary Therapy in Acromegaly: Results of a Prospective Multicenter Clinical Trial. *J Clin Endocrinol Metab* 2014; **99**: 1282–90.

6.2. Will any calculations or transformations be used to derive any outcome from the original data?

The change from baseline in cranio-caudal tumour diameter and tumour volume will be calculated by subtracting the baseline measurement from the end-of-treatment measurement. Transformations of tumour size variables for further analysis will be performed in case of skewed distribution (eg, square root or logarithmic transformation for positive skewness).

The percentage change in tumour volume is calculated as the absolute change in tumour volume divided by the measurement at baseline x 100.

The time to tumour progression is calculated as the number of weeks between start of study treatment and the first subsequent MRI showing a clinically significant increase in tumour volume. Participants who dropout without significant tumour progression at time of dropout or participants who experience no tumour progression up to week 72 will be regarded as censored.

The scores on the SF-36 components are converted to a 0-100 scale following the manual [14].

References

[14] Ware JE, Snow KK, Kosinski M, Gandek B. SF-36 Health Survey Manual and Interpretation Guide. Boston, MA: New England Medical Center, The Health Institute; 1993.

6.3. What analysis method will be used and how the treatment effects will be presented?

The main analysis of the effect of lanreotide versus placebo on primary outcome change in cranio-caudal diameter and secondary outcome change in tumour volume will be performed using ANCOVA in the intention-to-treat population, with end-of-treatment measurement as dependent variable, baseline measurement as continuous covariate and treatment as categorical covariate (0=placebo, 1=lanreotide) [15]. The effect sizes will be expressed as adjusted mean differences with 95% confidence interval and associated p-values.

A supplemental analysis for the primary outcome change in cranio-caudal diameter and secondary outcome change in tumour volume will be performed in the per-protocol population, with the same ANCOVA model.

The analysis for the secondary outcome time to tumour progression will be performed using the stratified log-rank test, with stratification for presence or absence of documented tumour growth at baseline (which is defined as growth in any direction on pituitary MRIs performed up to three years before enrolment). Group differences in hazard ratio with 95% confidence interval will be estimated with a Cox proportional-hazards model with terms for treatment group and tumour growth at baseline.

The analysis for the secondary outcome change in quality of life will be performed using ANCOVA for each component score in the intention-to-treat population, with score at end-of-treatment as dependent variable, score at baseline as continuous covariate, and treatment as categorical covariate (0=placebo, 1=lanreotide). The effect sizes will be expressed as adjusted mean differences with 95% confidence interval and associated p-values.

The number and type of adverse events will be summarised descriptively.

References
[15] Clifton L, Clifton DA. The correlation between baseline score and post-intervention score, and its implications for statistical analysis. *Trials* 2019; **20**: 43.

6.4. Will any assumptions for statistical methods be checked?

Normality of continuous variables will be assessed primarily by visual inspection of histograms and q-q plots, and secondly using the Shapiro-Wilk test (p-value < 0.05 is regarded as evidence for non-normality). Continuous variables judged to follow a non-normal distribution will be summarised using medians and interquartile ranges. TM Boertien will make the decision in consultation with PH Bisschop and MWT Tanck.

For the ANCOVA and linear mixed effects models, assumptions for linearity, homoscedasticity and normality of residuals will be checked for each model using scatter plots and residual analysis. Clearly positively skewed tumour size variables will be transformed using a natural logarithm (base *e*) before further analysis. Slightly skewed variables can be analysed without transformation depending on normality of residuals.

Regarding secondary outcome time to tumour progression, the assumption of proportional hazards will be checked using a time-dependent variable survival analysis.

6.5. Will sensitivity analyses be performed?

No outcome-definition related sensitivity analyses will be performed.
In case of missing outcome data, sensitivity analyses for missing data will be performed (see section 6.7).

6.6. Will subgroup analyses be performed?

There are no pre-specified subgroup analyses.

6.7. How will missing data be reported in the study reports and handled in the statistical analysis?

For the main analysis of the primary outcome change in cranio-caudal diameter and secondary outcome change in tumour volume, all observed data up to study discontinuation is used for analysis. This requires assessment of outcomes at time of study discontinuation. In case such outcome data is not obtained, the assumption that data is missing-at-random (MAR) will be checked. If the MAR assumption is plausible, participants who lack any post-baseline data are not required to be included in the initial analysis [16], although data missing-not-at-random (MNAR) can never be ruled out completely.

In case of missing outcome data, the following analyses will be performed:
- Exploration of the missing mechanism via Missing Value Analysis and the relation between missing data and several variables via group comparisons and logistic regression [17,18]. Pre-specified baseline variables to be checked will be age, sex, baseline tumour size. Post-baseline variable to be checked will be significant tumour size increase.

- Multiple imputation of missing outcome data [17,19]. Univariate missing data will be imputed via a regression model, and multivariate missing data via MICE (multivariate imputation by chained equations). Predictor variables will be all observed outcome data (including baseline measurement), and any variables that are found to be significant predictors of dropout, as checked in the explorative analysis. Multiple imputation will be performed separately in each treatment group. The number of imputed datasets will be based on the percentage of missing data [20]. Precision parameters will be checked. After imputation, the ANCOVA as specified for the main analysis will be performed in each imputed dataset, and results pooled following Rubin's rules.

- Linear mixed effects model to include all repeated MRI data (allowing for within-subject correlated data) and account for study discontinuation at different time-points. In the model, treatment group, the number of injections after which MRI was performed, and the interaction between these two will be entered as fixed effects; a by-subject random intercept will be entered as random effect [21,22].

- Both multiple imputation as the linear mixed effects model handle missing data under the MAR assumption. To explore departures from this assumption, a pattern-mixture model will be performed as sensitivity analysis, with controlled multiple imputation of data under alternative post-dropout scenarios [18,19]. In this model, the MAR imputed data is shifted by a range of offsets, termed δ (delta). One scenario will be that dropouts have a poorer response (i.e., faster tumour growth). The δ-range will be based on increasing percentages of the mean change from baseline in the observed data from participants who have completed the study (range of 25-50-75-100-200%), and these are added to the MAR imputed data. Another scenario can include reason for dropout, where, eg, participants who dropout due to tumour progression are assigned a larger δ, while participants who dropout due to adverse events have missing data imputed under MAR. For each δ, data are re-analysed with ANCOVA and results pooled. If results are qualitatively maintained for a range of plausible δ, the results under MAR are considered robust. The pattern-mixture model will also include a so-called 'tipping point analysis', to assess which shift in imputed MAR data is required to overturn the conclusion of the main analysis, and whether such a shift would be considered (clinically) plausible.

For the secondary outcome change in quality of life, the mean imputation method will be used to impute missing component score values up to study discontinuation.

References

[16] White IR, Carpenter J, Horton NJ. Including all individuals is not enough: Lessons for intention-to-treat analysis. *Clin Trials* 2012l **9**:396–407. https://doi.org/10.1177/1740774512450098
[17] Heymans MW, Eekhout I. Applied Missing Data Analysis With SPSS and R(Studio). Amsterdam: Heymans and Eekhout, 2019 https://bookdown.org/mwheymans/bookmi/.

[18] Carpenter JR, Kenward MG. Missing data in randomised controlled trials–a practical guide. *Birmingham Natl Coord Cent* … 2007. http://www.hta.nhs.uk/nihrmethodology/reports/1589.pdf.
[19] Cro S, Morris TP, Kenward MG, Carpenter JR. Sensitivity analysis for clinical trials with missing continuous outcome data using controlled multiple imputation: A practical guide. *Stat Med* 2020; **39**: 2815–42.
[20] White IR, Royston P, Wood AM. Multiple imputation using chained equations: Issues and guidance for practice. *Stat Med* 2011; **30**: 377–99.
[21] Winter B (2013). Linear models and limear mixed effects models in R with linguistic applications. arXiv: 1308.54999. [http:///arxiv.org/pdf/1308.5499.pdf]
[22] Douglas Bates, Martin Maechler, Ben Bolker, Steve Walker (2015). Fitting Linear Mixed-Effects Models Using lme4. Journal of Statistical Software, 67(1), 1-48. doi:10.18637/jss.v067.i01.

6.8. Will additional analyses on the primary or secondary outcomes be performed?

No additional analyses will be performed on the primary or secondary outcomes next to those specified in section 6.3 and 6.7.

6.9. How will harms be reported?

Safety/harms will be reported as the number, type, seriousness and severity (mild-moderate-severe) of adverse events recorded during study participation. Adverse events are coded using the Medical Dictionary for Regulatory Activities (MedDRA). Possible relatedness to lanreotide treatment will be reported as well. Most commonly expected adverse drug reactions following treatment with lanreotide are: gastrointestinal disorders (eg, diarrhea, nausea, abdominal pain), cholelithiasis (often asymptomatic), injection site reactions, and changes in glycemic control ([mild] hypoglycaemia or hyperglycaemia). Tumour progression in itself will not be reported as adverse event, but visual disturbances due to optic chiasm compression or surgical intervention will be listed.

Adverse events will be presented using tabulations of counts and percentages of events and of patients experiencing one or more of each type of event for each treatment arm. No formal statistical testing will be performed.

6.10. Which statistical software will be used to carry out the statistical analyses?

Analyses will be performed in SPSS, version 28 or later if available (IBM, Armonk, NY, USA). The linear mixed effects model will be run in R, version 4.2.0 (April 2022) or later if available (R Foundation for Statistical Computing, Vienna, Austria) [23], using the *lme4* package [22].

References
[22] Douglas Bates, Martin Maechler, Ben Bolker, Steve Walker (2015). Fitting Linear Mixed-Effects Models Using lme4. Journal of Statistical Software, 67(1), 1-48. doi:10.18637/jss.v067.i01.
[23] R Core Team (2022). R: A language and environment for statistical computing. R Foundation for Statistical Computing, Vienna, Austria. URL https://www.R-project.org/

Section 7. References to literature, standard operating procedures and reporting guidelines.

7.1. Are non-standard statistical procedures to be used, which have not been described in sufficient depth in the previous sections?

Not applicable.

7.2. What is the title, date and version number of the current data management plan?

The current data management plan is titled: “Data management plan – GALANT study”, version number is 3, date is 21-02-2023.

The data management plan is stored in the trial master file.

7.3. What is the title, date and version number of the current data validation and derivation plan?

Data validation and derivation has been built directly into Castor EDC. Validation checks on completeness, correctness and consistency are incorporated in the data collection system and have been documented: listed in the blank eCRF form saved as PDF “Castor eCRF GALANT v107.04 (final) - study complete”, located digitally in the trial master file at: G:\diva\Endocrinologie\Onderzoek\2015_103-GALANT\1. TMF&ISF GALANT 2015_103 - digitaal\15. Data Management & Statistiek

All used calculations in the database are listed in a separate document “Castor EDC GALANT calculations formula” at the same digital location.

7.4. Where is the study master file stored?

The trial master file is stored:
- physically at: Amsterdam UMC, location AMC, K2-276-2 (dept. of Endocrinology & Metabolism)
- digitally at: G:\diva\Endocrinologie\Onderzoek\2015_103-GALANT\1. TMF&ISF GALANT 2015_103 – digitaal

7.5. Where are the syntax files for data extraction, manipulation and preparation and statistical analysis stored?

The syntax files for data extraction, manipulation and preparation and statistical analysis are stored at: G:\diva\Endocrinologie\Onderzoek\2015_103-GALANT\5. Analyses

7.6. Which standard operating procedures will be adhered to when using and analysing data from this study?

When using and analysing data from the GALANT trial, researchers will adhere to the standard operating procedure *AMC RDM001 Research data management*.

**7.7. Which reporting guidelines will be adhered to when reporting on this study?**
When reporting the results of this randomised clinical trial, we will adhere to the "CONsolidated Standards Of Reporting Trials" (CONSORT) guidelines.

Appendix.

Figure 1. Mock-up of the CONSORT flow diagram for the GALANT trial.

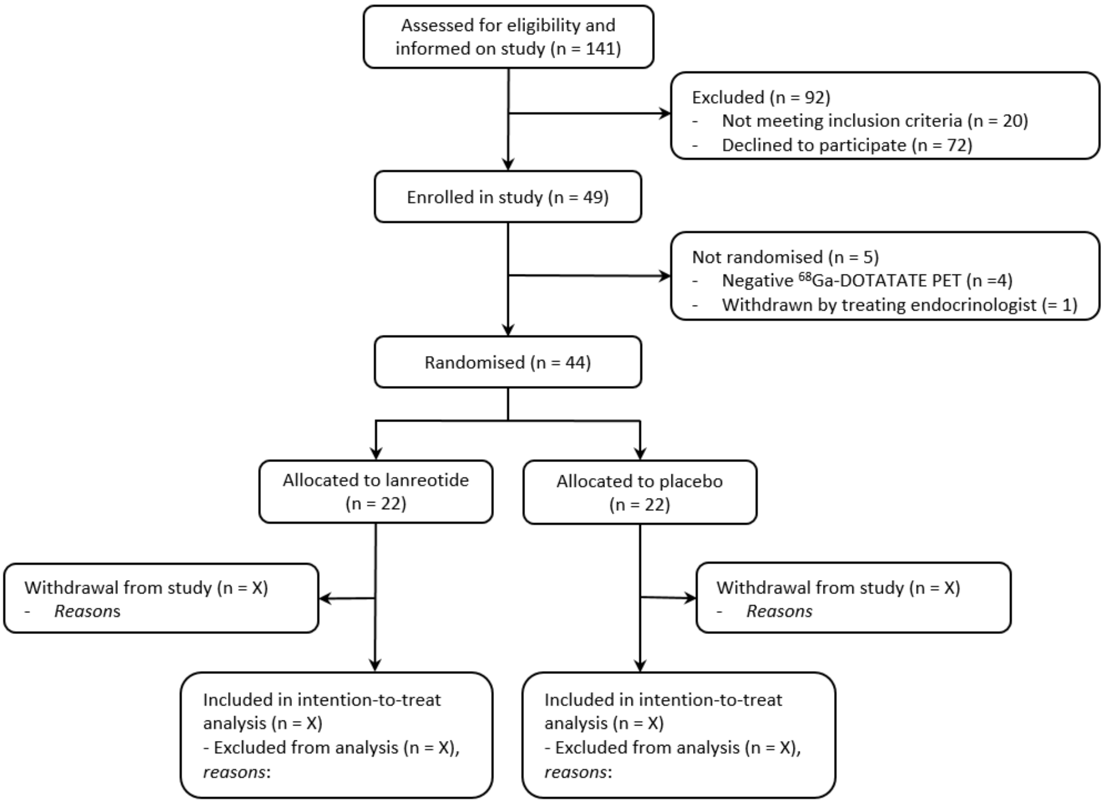

ADDENDUM TO THE STATISTICAL ANALYSIS PLAN – GALANT TRIAL

date: 4 March 2024

The following adjustments have been made to the statistical methods/analyses following manuscript revisions after submission.

With regard to:

- section 2.2: regarding study objectives, the main analysis can be regarded as the 'while-on-treatment' estimand. The estimand framework officially came into effect in 2020 as an addendum to the ICH E9 guideline "Statistical principles for clinical trials"[1], several years after initiation of our trial and the final protocol version. As thus, estimands were not explicitly included in the trial protocol or in the statistical analysis plan (SAP). With regard to our trial objective, this can be described as the 'while-on-treatment' strategy, where all observed data up to an intercurrent event (such as treatment discontinuation) is used in the analysis.[1] This strategy is similar to a 'last observation analysis' and is an assessment of effectiveness in clinical practice. Note that this is not equal to 'last observation carried forward' (LOCF) imputation, as nothing is 'carried forward'/imputed and results are interpreted as the change actually observed at the last observation.[2] Since treatment discontinuation due to various reasons is expected to occur regularly in real life, we considered this an acceptable strategy for the main analysis. It is still considered to adhere to the ITT principle, as all randomised patients are analysed in the groups to which they were randomised.

- section 3.4, 6.3 and 6.9: the SAP states that the outcome of safety/harms (based on adverse events) will be summarised descriptively without formal statistical testing. Following implementation of the CONSORT Harms 2022 statement, between-group absolute risk difference estimates with Wilson 95% confidence intervals were calculated post-hoc. Due to events being rare or null in one or both treatment arms, relative risk estimates were not calculated.[3]

- section 6.7: the final linear mixed effects model has been specified more comprehensively, with addition of the fixed effects of baseline tumour size and baseline size-by-time interaction. The outcome/response vector has subsequently been adjusted to only contain post-baseline measurements.[4] As an additional post-hoc optimisation, a residual correlation matrix was specified to address possible residual serial autocorrelation not sufficiently accounted for by the random intercept.[5] Furthermore, a post-hoc mixed model for repeated measurements (MMRM) has been fitted.[6,7]

- section 6.10: the SAP states that the *lme4* package in R will be used for the linear mixed effects model. This has been changed to the *nmle* package[8] in order to specify the residual correlation structure.

References

1 European Medicines Agency. ICH E9 (R1) addendum on estimands and sensitivity analysis in clinical trials to the guideline on statistical principles for clinical trials - Step 5, EMA/CHMP/ICH/436221/2017. 2020 https://www.ema.europa.eu/en/documents/scientific-guideline/ich-e9-r1-addendum-estimands-and-sensitivity-analysis-clinical-trials-guideline-statistical-principles-clinical-trials-step-5_en.pdf.

2 Mallinckrodt CH, Kenward MG. Conceptual Considerations regarding Endpoints, Hypotheses, and Analyses for Incomplete Longitudinal Clinical Trial Data. *Drug Inf J* 2009; **43**: 449–58.

3 Junqueira DR, Zorzela L, Golder S, *et al.* CONSORT Harms 2022 statement, explanation, and elaboration: updated guideline for the reporting of harms in randomised trials. *BMJ* 2023; : e073725.

4 Dinh P, Yang P. Handling baselines in repeated measures analyses with missing data at random. *J Biopharm Stat* 2011; **21**: 326–41.

5 Molenberghs G, Verbeke G. A Model for Longitudinal Data. In: Linear Mixed Models for Longitudinal Data. New York, NY: Springer New York, 2000: 19–29.

6 Mallinckrodt CH, Lane PW, Schnell D, Peng Y, Mancuso JP. Recommendations for the Primary Analysis of Continuous Endpoints in Longitudinal Clinical Trials. *Drug Inf J* 2008; **42**: 303–19.

7 Molenberghs G, Thijs H, Jansen I, *et al.* Analyzing incomplete longitudinal clinical trial data. *Biostatistics* 2004; **5**: 445–64.

8 Pinheiro JC, Bates DM, Team RC. nlme: Linear and Nonlinear Mixed Effects Models. 2023. https://cran.r-project.org/package=nlme.
