# Supplementary material for: Lanreotide versus placebo for tumour reduction in patients with a 68Ga-DOTATATE PET-positive, clinically non-functioning pituitary macroadenoma (GALANT study): a randomised, multicentre, phase 3 trial with blinded outcome assessment
Source: Lancet Reg Health Eur. 2024 May 13;42:100923. doi: 10.1016/j.lanepe.2024.100923 (PMC11281922; doi:10.1016/j.lanepe.2024.100923)
Supplement: Data Management Plan GALANT [file mmc4.pdf]

Data management plan – GALANT study

The following study info provides relevant information regarding data management requirements:

Study number (ABR/METC)	ABR NL52821.018.15<br>METC 2015_103
Study (acronym or short title)	GALANT
Sponsor (Verrichter/initiator/coördinerende hoofdonderzoeker)	AMC, PI prof. E. Fliers
Department	Endocrinology & Metabolism
Mono/Multicenter & (inter)national	Multicentre, national
(non)WMO	☒ WMO compliant<br>☐ not WMO compliant
Summary of the project	The GALANT study is a multicentre, randomised, double-blind, placebo-controlled, parallel-group, phase 3 trial in patients with a ^{68}Ga-DOTATATE PET-positive non-functioning pituitary macroadenoma, investigating the effect of lanreotide versus placebo on tumour size. Forty-four patients with a PET-positive non-functioning pituitary macroadenoma are randomised in a 1:1 ratio between 4-weekly injections of lanreotide 120mg or placebo (saline). The primary outcome measure is the absolute change in cranio-caudal adenoma diameter after 72 weeks of treatment. Secondary outcomes are change in tumour volume, time to tumour progression, and change in quality of life based on SF-36 component scores. Safety is assessed on the basis of the number, type and severity of adverse events during the study. The study is conducted in an outpatient setting and eligible patients are referred by endocrinologists at academic and non-academic hospitals in the Netherlands for inclusion at one of the participating centres (Amsterdam University Medical Centres (locations AMC and VUMC) and Leiden University Medical Centre). The study-related ^{68}Ga-DOTATATE PET/CT is performed at either the AMC or the Netherlands Cancer Institute in Amsterdam, an imaging-only participating centre.
Co-ordinating PI (Hoofdonderzoeker)	Prof. Eric Fliers, e.fliers@amsterdamumc.nl
Responsible for completion of the data management plan	Research coordinator Tessel Boertien, t.m.boertien@amterdamumc.nl

Funding by	Ipsen Farmaceutica BV
Consulted data management expert	Beer Franken, privacy functionary, currently not employed anymore at the AMC<br><br>Rudy Scholte, head of Data management AMC, r.a.scholte@amsteramumc.nl, consulted i.a. November 2016
Data management plan version / date	V3, 21-02-2023
PI signature for approval of this DMP	

Phase 1: Study preparation

Privacy and security safeguards

1.1

☐ The data set is anonymous and cannot be linked to any subject

☒ The data set is encoded; a meaningless unique code (Subject ID) is used, and subjects can be identified through a subject identification log

☐ The data set is directly identifiable

For any selected option: explain why this is necessary: Encoded instead of anonymous, needed for i.a. source data validation and communication of results to participating patients

1.2

☒ Data are de-identified within the study database (see 1.1)

☐ Additional de-identification steps are taken, *specify: …*

1.3

☐ A Data Protection Impact Assessment (DPIA) has been performed, *specify document: …*

☒ The data acquisition has been registered at the DPO, *specify registration number of the record of processing activities (Verwerkingsregister):* AMC2015-22. (DPIA not necessary for studies approved/initiated before 25 May 2018)

1.4

☒ The study has been (pre)registered or a concept/design paper has been published, *specify registration number(s):* Registration: Netherlands Trial Registry NL5136, and EudraCT 2015-001234-22

Publication of study design: doi 10.1136/bmjopen-2020-038250

1.5

☒ An informed consent procedure has been set up that describes the data set, time span of data retention, information on sharing data or making data available for future research

1.6

☒ A central location for all digital study documents and (references) to data exists; *specify:* G:\diva\Endocrinologie\Onderzoek\2015_103-GALANT at location AMC

☒ A central location for all hard copy study documents exists; *specify:* K2-276-2 (at dept. of Endocrinology & Metabolism of location AMC), here the study TMF and ISSF are stored

Data acquisition

General

1.7

Describe the data acquisition per type of data

☒ Reuse of existing data, e.g., patient characteristics from EPIC, or data from other research; *specify:* age, height & weight, medical & pituitary specific history, medication use, intoxications, lab results performed for clinical practice during the study, MRI reports performed for clinical practice during the sudy. No data from other research was used.

☒ Use of measured data, e.g., lab data; *specify:* 68-Ga-DOTATATE PET-CT scans (including coregistration and SUV measurement); MRI tumour size and volume measurements

☒ Data collection, e.g., clinical data in eCRFs, questionnaires; *specify:* study interviews focussed on changes in medication use and adverse events (source data in electronic patient file); paper SF-36 questionnaires; measurement of weight, blood pressure and heart rate at study visits

1.8

Describe the terminology standards, classifications or existing data definitions that have been applied in the data set: …

1.9

☒ All acquired data, either reused, measured, or manually collected, are described in a data dictionary or code book

→ all this information is stored in the Castor EDC eCRF, of which a complete PDF is available in the digital TMF.

1.10

Data acquisition type	Description	Type	Format	Size estimate	Sensitivity	Terminology standard
1. Trial documents<br>2. Participant documents<br>3. Collected data<br>4. Analysis data<br>5. Coded MRI scans + measurements	1. TMF/ISF<br>2. ISSF1<br>3. ISSF2-3 (i.a. CRF and SF-36 scans, imported MRI/PET CD's)<br>4. Analysis folder<br>5. MRI ITK-SNAP folder	1. PDFs, word doc's<br>2. PDFs, Word doc's<br>3. PDFs, DICOM files<br>4. SPSS, R and excel files<br>5. MRI DICOM and ITK-SNAP files	1. .pdf, .docx, .xlsx<br>2. .pdf, .docx<br>3. .pdf, .dicom<br>4 .sav, .sps, .R, .csv, .nqt<br>5. .dicom, .itksnap, .nii	1. ~2GB<br>2. ~100MB<br>3. ~7GB<br>4. ~500MB<br>5. ~ 32GB	1. Low/non-sensitive data<br>2. Highly sensitive personal data<br>3. Medium sensitive data (encoded)<br>4. Medium sensitive data (encoded)<br>5. Medium sensitive data (encoded)	NA

Reuse of existing data ☐ Not applicable

1.11

Specify the source that is used to acquire the existing data: EPIC (electronic patient file), not from other/previous research

1.12

☒ The reuse of existing data for this study is covered by the subject's informed consent

1.13

☐ The party that delivers encoded data remains responsible for the subject identification log for their own subjects – **N.A.**

Measured data ☐ Not applicable

1.14

Specify the device that generates the measured data: not perfectly clear what is meant by 'device'…

**1.15**	☐ No user training is required; the device that generates the data is self-explanatory<br>☐ No separate user training is required; users are already acquainted with and trained in using the device for the type of measurements that are required for this study<br>☒ Users are trained in using the device /performing the lab measurements in the way needed for this study this is documented
Data collection	☐ Not applicable
**1.16**	☒ An electronic CRF is used to collect all or a part of the data, a copy of the blank CRF pages is kept as back-up<br>☒ A paper CRF is used to collect all or a part of the data: paper CRFs were used for 1) logging of injection administration by the Endocrinology nurses at the Endocrine Unit of the AMC, 2) the LUMC visits performed by the research nurse there, 3) the centralised MRI measurements by 2 independent outcome assessors, 4) for other study data at the start of the study before implementation of Castor electronic CRF. All data collected through paper CRFs was subsequently entered in the electronic CRF.
**1.17**	*Specify the name of the data collection system:* Castor EDC<br>☒ Licensing and a Processing agreement have been arranged
**1.18**	☒ The developer of the eCRFs and/or questionnaires in the data collection system is already acquainted with the system: TM Boertien<br>☐ The developer of the eCRFs and/or questionnaires in the data collection system is trained in using the system and this is documented
**1.19**	☒ The database was designed before it was built and a data dictionary was created: Database was designed & tested before activation, the data dictionary is implemented in Castor EDC, all variables including field type, field options, checks and warning messages are listed in the blank eCRF form saved as PDF “Castor eCRF GALANT v107.04 (final) - study complete”, located at: G:\diva\Endocrinologie\Onderzoek\2015_103-GALANT\1. TMF&ISF GALANT 2015_103 - digitaal\15. Data Management & Statistiek
**1.20**	☒ Validation checks on completeness, correctness and consistency are incorporated in the data collection system and have been documented: These are implemented in Castor EDC and can be found in the blank eCRF PDF document listed above. All calculations are listed in a separate document “Castor EDC GALANT calculations formula” at the same digital location.
**1.21**	☒ The data collection system has been tested by both the study team and an independent party.<br>☐ The test findings, follow-up of the findings and final approval are documented.<br>A CRF checklist was completed for the paper CRF on 01-09-2015 and approved by the PI E. Fliers. The electronic CRF in Castor EDC has been tested before setting the study to live for the first time by TM Boertien. Errors were corrected directly in the system, not documented separately. Tests were not done by an independent party.
**1.22**	☒ Access to the data collection system is based on individual login with only the necessary access rights<br>☒ Access to the data collection system is managed (under supervision of) and documented by the PI
**1.23**	☒ The data collection system logs the identity of the persons using the system
**1.24**	☒ Procedures for data collection are included in the data collection system<br>☐ Procedures for data collection are documented in a manual, *specify document name and location: …*
**1.25**	☐ No user training is required; the data collection system is self-explanatory<br>☐ No user training is required; users are already familiar with the data collection system (only non-WMO)<br>☒ Users are trained in the data collection system, and this is documented: Documented on the GALANT Study training log (and all users are specified on the Site signature and delegation log), physically located in the TMF (trial master file) at AMC location K2-276-2, and digitally scanned after signing off at: G:\diva\Endocrinologie\Onderzoek\2015_103-GALANT\1. TMF&ISF GALANT 2015_103 - digitaal\7. Trial Site Staff - all sites
**1.26**	☒ For each study-specific data collection, source documentation is available<br>☐ For the following parts of data collection, no source documentation is available, as described in the protocol; *specify: …*
**Data storage (both during study and for archiving)**	
**1.27**	What data (raw files, intermediate files, final files, subject identification log (key file)) is stored on which location during the study?

Location	What data?
On the department’s G-drive (location AMC)<br>- G:\diva\Endocrinologie\Onderzoek\2015_103-GALANT	☒ Raw ☒ Interim<br>☒ Final ☐ Key
On a physical location (location AMC)<br>- K2-276-2, dept. Endocrinology & Metabolism	☐ Raw ☐ Interim<br>☒ Final ☒ Key

	On a data storage facility at Amsterdam UMC or at an Amsterdam partner	☐ Raw ☐ Interim<br>☐ Final ☐ Key
	On an external data storage facility	☐ Raw ☐ Interim<br>☐ Final ☐ Key
**1.28**	☒ The size of the data is <50 gigabyte during data collection and *to be determined* gigabyte when archiving the project<br>☒ Budget is allocated both for storage during the study as well as for archiving upon completion of the study<br>☐ Budget is allocated for data management activities and creating a FAIR dataset	
**1.29**	☒ The study-specific folder with data files is only accessible by study team members<br>☒ Access to study data is managed and documented by the PI	
**Subject identification log**		☐ Not applicable
**1.30**	☒ The subject identification log(s) is/are kept separate from other study related data	
**1.31**	☒ In multicentre studies the site-specific subject identification log(s) is/are kept on site only and will <u>not</u> be shared centrally or with other sites.	

Legal issues/agreements/matters	☐ Not applicable
**1.32**	For collaboration with the research partners or for data transfer or data sharing, written agreements on data management, privacy, data ownership and intellectual properties are made, namely:<br>☒ A collaboration agreement with consortium partners or project members, e.g., consortium agreement or clinical trial agreement, *specify:* agreement with Ipsen Farmaceutica BV, with Eurocept Homecare, and with the participating centres.<br>☐ An agreement with other parties, e.g., data sharing agreement or material transfer agreement, *specify: …*
Additional information on Phase 1: Study preparation (provide item number)	

**Phase 2: Data collection**	
**General**	
**2.1**	☒ A site signature and delegation log of all people involved in the data collection is kept by the PI
**2.2**	☒ *If applicable:* a procedure for deblinding is in place and has been documented
**Reuse of existing data / use of measured data**	☐ Not applicable
**2.3**	☒ The reused existing data and/or the raw, measured data are stored as read-only file and a copy is made for further processing
**Externally acquired data**	☐ Not applicable
**2.4**	☒ Identifiable data is removed or encoded by the external party, prior to sharing the data
**2.5**	☒ Data are transferred in a secure way by SURF Filesender *(or specify which secure transfer method is used: …)*
**Quality control**	
**2.6**	☒ Checks on completeness, correctness and consistency are built into the system (as specified in the *Data Validation and Derivation Plan* – note: this has been directly been into Castor EDC*)*<br>☒ Non-automated checks such as manual checks and data monitoring are performed and are documented
**2.7**	☒ Completion of multicentre data collection is signed off by the local PI per patient and the coordinating PI for completion of data collection
**Change control**	☐ Not applicable
**2.8**	How are changes to the **data** handled?<br>☒ Documented on the paper data collection tool (pCRF or questionnaire)<br>☒ Audit trail or 'track changes' functionality in the applied system<br>☒ Reason for change is documented, e.g., 'Confirm changes' setting in Castor is used<br>☐ Other change control; *specify: …*
**2.9**	How are changes to the **design** of the data collection handled?<br>☒ By creating a new version of the paper data collection tool (pCRF or questionnaire)<br>☒ Audit trail or 'track changes' functionality in the system<br>☒ Other change control procedures: separate Castor version controle log document where all changes to the eCRF are noted for clear overview, located at G:\diva\Endocrinologie\Onderzoek\2015_103-GALANT\1. TMF&ISF GALANT 2015_103 - digitaal\15. Data Management & Statistiek<br>☐ All changes in the design will be documented to ensure an impact assessment of these changes is performed
**Locking a data collection**	☐ Not applicable
**2.10**	*Describe how the data collection system is locked: …*<br>☒ Using the locking functionality in the system<br>☐ Other; *specify: …*
**2.11**	☒ Approval and reason for locking have been documented
**2.12**	☒ The Statistical Analysis Plan is finalized, prior to (deblinding and) analysing the data
Additional information on Phase 2: Data collection (provide item number)	

Phase 3: Processing & statistical analysis	
**Export to the data processing and statistical environment** ☐ Not applicable	
**3.1**	☒ The data are stored in a generic and machine actionable format; *specify:* SPSS .dat format, and in Excel format.<br>☐ Data are also stored in another format; *specify: …*
**3.2**	☒ *Specify the software system including version number, applied for processing and statistical analysis:*<br>Note: later versions may be used if available at time of analysis<br>SPSS, version 28 (IBM, Armonk, NY, USA).<br>R, version 4.2.0 (April 2022; R Foundation for Statistical Computing, Vienna, Austria)
**Performing data processing and statistical analysis** ☐ Not applicable	
**3.3**	☒ The acquired data are stored as read-only file and a new file is created for further processing and statistical analysis
**3.4**	☒ All data processing and analysis is programmed in syntax or script files
**3.5**	☒ Descriptive comments are added to the syntax or script files
**3.6**	☒ Data sets and syntax or script files are placed under version control
**3.7**	☐ Data corrections in this phase are made in the original source<br>☒ Data corrections in this phase are programmed in syntax or script files
**Sharing data for processing or statistical analysis** ☒ Not applicable	
**3.8**	☐ Data processing and analysis by an external party is covered by the informed consent
**3.9**	☐ Data are transferred in a secure way by SURF Filesender or *specify which secure transfer is used: …*
Additional information on Phase 3: Processing & statistical analysis (provide item number)	

Phase 4: Writing & publishing	
**Organizing files**	
**4.1**	☒ For each manuscript a structured subfolder has been created
**Findability of the data set**	
**4.2**	☒ The manuscript will be published in an Open Access journal that provides a PID (e.g., a DOI or URN)<br>☒ The information regarding the data collection can be found through a catalogue or repository; *specify*: to be determined<br>☐ This catalogue or repository creates a PID<br>☐ This catalogue or repository has a CoreTrustSeal (or other certification; *specify: …*)<br>☐ To make my (meta)data findable, we will crosslink any online sources where applicable *(e.g., ORCIDs of researchers, PIDs of related publications or repository references within the project, trial registry numbers, project website, etc.)*<br>☐ I will not publish my metadata and/or data; *explain why not …*
Additional information on Phase 4: Writing & publishing (provide item number)	

Phase 5: Data sharing and archiving		
**5.1**	What will be published? To be determined<br>☐ Metadata (see 5.2)<br>☐ The raw, pre-processed data; *specify location (e.g., at Amsterdam UMC or externally): …*<br>☐ The final data; *specify location: …*<br>☐ Other (e.g. Software); *specify type and location: …*	
**5.2**	What metadata is provided about the **study?**<br>☒ Study protocol<br>☒ Statistical Analysis Plan<br>☒ Data Management Plan	 ☐ (Amsterdam UMC) metadata schema<br>☐ Other; *specify …*
**5.3**	☒ Data reuse of the dataset is covered in the informed consent procedure	

	☒ Information regarding the subset of the data for people who consented to reuse is available<br>☒ Procedures for withdrawal of consent for reuse have been defined<br>☐ A more pseudonymized version of the dataset has been created for reuse in consultation with the DPO<br>☐ For verification purposes, all data are stored internally (see 5.5 for data access procedures).
**5.4**	What metadata is provided about the **data**, including processing and analysis?<br>☒ Documentation on study procedures ☒ Syntaxes or scripts Other; specify: …<br>☒ Data dictionary ☐ Software<br>☐ Data validation and derivation plan ☐ Hardware
**5.5**	☐ The research data will be publicly accessible without any restrictions<br>☒ Conditions for reuse apply; *provide a short description:* to be determined<br>☒ An embargo period applies; *specify reason and duration:* to be determined<br>☐ At the time of the journal article's publication
**5.6**	☐ For data reuse, a Data Sharing Agreement or equivalent will be set up
**Transfer to an external party**	☒ Not applicable
**5.7**	☐ (Copies of) original data and documentation are kept at Amsterdam UMC<br>☐ All data transfers have been documented
**5.8**	☐ Data are transferred in a secure way by SURF Filesender (or *specify which secure transfer is used: …)*
**Digital archiving**	
**5.9**	☒ *Digital data and documentation will be preserved for* 15 *years. This includes:*<br>☒ metadata ☒ raw data files ☒ final data files<br><br>*If any of these boxes is not ticked, explain: …*
**5.10**	*Specify the location of the digital archive:* to be determined
**5.11**	☒ A subject identification log is archived and kept separate from other study related data. This does not conflict with the subject's informed consent
**Paper archiving**	☐ Not applicable
**5.12**	☒ Paper documentation will be preserved for: 15 years
**5.13**	Specify the physical location of the paper archive: archive of the Amsterdam UMC, location AMC.
Additional information on Phase 5: Data sharing & archiving (provide item number)	
