# Supplementary material for: Lanreotide versus placebo for tumour reduction in patients with a 68Ga-DOTATATE PET-positive, clinically non-functioning pituitary macroadenoma (GALANT study): a randomised, multicentre, phase 3 trial with blinded outcome assessment
Source: Lancet Reg Health Eur. 2024 May 13;42:100923. doi: 10.1016/j.lanepe.2024.100923 (PMC11281922; doi:10.1016/j.lanepe.2024.100923)
Supplement: CONSORT 2008 abstract checklist [file mmc6.pdf]

CONSORT 2008 checklist of Items to Include when Reporting a Randomized Trial in a Journal or Conference Abstract

Item	CHECK	Description
Title	✓	Identification of the study as randomized
Authors *	NA	Contact details for the corresponding author
Trial design	✓	Description of the trial design (e.g. parallel, cluster, non-inferiority)
Methods		
Participants	✓	Eligibility criteria for participants and the settings where the data were collected
Interventions	✓	Interventions intended for each group
Objective	✓	Specific objective or hypothesis
Outcome	✓	Clearly defined primary outcome for this report
Randomization	✓	How participants were allocated to interventions
Blinding (masking)	✓	Whether or not participants, care givers, and those assessing the outcomes were blinded to group assignment
Results		
Numbers randomized	✓	Number of participants randomized to each group
Recruitment	NA	Trial status
Numbers analysed	✓	Number of participants analysed in each group
Outcome	✓	For the primary outcome, a result for each group and the estimated effect size and its precision
Harms	✓	Important adverse events or side effects
Conclusions	✓	General interpretation of the results
Trial registration	✓	Registration number and name of trial register
Funding	✓	Source of funding

*This item is specific to conference abstracts.

NA = not applicable

doi:10.1371/journal.pmed.0050020.t001
